# Systematic Structure-Activity Relationship (SAR) Exploration of Diarylmethane Backbone and Discovery of A Highly Potent Novel Uric Acid Transporter 1 (URAT1) Inhibitor

**DOI:** 10.3390/molecules23020252

**Published:** 2018-01-27

**Authors:** Wenqing Cai, Jingwei Wu, Wei Liu, Yafei Xie, Yuqiang Liu, Shuo Zhang, Weiren Xu, Lida Tang, Jianwu Wang, Guilong Zhao

**Affiliations:** 1 School of Chemistry and Chemical Engineering, Shandong University, Jinan 250100, China; caiwenqingsunny@163.com; 2 Tianjin Key Laboratory of Molecular Design and Drug Discovery, Tianjin Institute of Pharmaceutical Research, Tianjin 300193, China; wujw@tjipr.com (J.W.); liuw@tjipr.com (W.L.); xieyf@tjipr.com (Y.X.); liuyq@tjipr.com (Y.L.); xuwr@tjipr.com (W.X.); tangld@tjipr.com (L.T.); 3 Shandong Key Laboratory for Special Silicon-Containing Materials, Advanced Materials Institute, Shandong Academy of Sciences, Jinan 250014, China; e50687e@163.com

**Keywords:** gout, hyperuricemia, URAT1 inhibitor, lesinurad, structure-activity relationship (SAR), synthesis

## Abstract

In order to systematically explore and better understand the structure-activity relationship (SAR) of a diarylmethane backbone in the design of potent uric acid transporter 1 (URAT1) inhibitors, 33 compounds (**1a**–**1x** and **1ha**–**1hi**) were designed and synthesized, and their in vitro URAT1 inhibitory activities (IC_50_) were determined. The three-round systematic SAR exploration led to the discovery of a highly potent novel URAT1 inhibitor, **1h**, which was 200- and 8-fold more potent than parent lesinurad and benzbromarone, respectively (IC_50_ = 0.035 μM against human URAT1 for **1h** vs. 7.18 μM and 0.28 μM for lesinurad and benzbromarone, respectively). Compound **1h** is the most potent URAT1 inhibitor discovered in our laboratories so far and also comparable to the most potent ones currently under development in clinical trials. The present study demonstrates that the diarylmethane backbone represents a very promising molecular scaffold for the design of potent URAT1 inhibitors.

## 1. Introduction

Gout is the most common inflammatory arthritis caused by the deposition of monosodium urate (MSU) in articular and periarticular tissues and characterized by recurrent joint swelling, redness, warmth and severe pain [1]. If left untreated or poorly managed, tophaceous gout will ultimately lead to permanent joint destruction, bone erosion and kidney impairment, dramatically affecting patients’ quality of life and even threatening their lives [2]. In addition, more and more evidence has been accumulated to demonstrate that hyperuricemia is likely an independent risk factor for hypertension, chronic kidney disease (CKD) and congestive heart failure (CHF) among others [3,4]. First identified by Egyptians in 2640 BC, gout is among the earliest diseases recognized as a clinical entity [5]. The prevalence and incidence of hyperuricemia and gout have been rising worldwide [6,7], and gout and its comorbidities present significant burdens on both the individual and community [8,9]. Hyperuricemia is the necessary condition of MSU formation and deposition, which is defined as elevated serum uric acid (sUA) levels above the saturation point of MSU in body fluid at physiological pH (7.4) and temperature, i.e., 6.8 mg/dL (404 μmol/L) [10]. Uric acid is a weak organic acid (pKa = 5.75), and therefore greater than 98% is in the ionized form, i.e., urate anion, at physiological pH (7.4); due to the presence of sodium ion at high concentrations in the extracellular compartment, urate largely presents as MSU [11].

Uric acid is the end-product of purine metabolism in humans due to the loss of uricase during the course of evolution; uricase is an enzyme capable of oxidizing highly insoluble uric acid to allantoin, which is 5–10 times more water-soluble than uric acid and can, therefore, be much more readily eliminated by the kidney [12]. Hyperuricemia is caused by over-production and/or under-excretion of urate, and among the patients with hyperuricemia, greater than 90% of them are urate under-excretors, whereas the remaining less than 10% are over-producers [13].

The treatment of gout can be divided into the anti-inflammatory treatment of acute gout attack and management of chronic gout. The standard therapy for acute gout is traditionally directed toward reducing inflammation with anti-inflammatory agents such as colchicine, nonsteroidal anti-inflammatory drugs (NSAIDs), corticosteroids and interleukin-1β blockers, which can effectively reduce gout flares but are also associated with a variety of adverse effects [14,15]. The mainstay of treatment of chronic gout is to reduce sUA <6 mg/dL (360 μmol/L) and even <5 mg/dL (300 μmol/L) for severe gout by urate-lowering therapy (ULT) with xanthine oxidase inhibitors (XOIs) such as allopurinol, febuxostat and topiroxostat, uricosuric agents such as probenecid, sulfinpyrazone, benzbromarone and lesinurad, and uricase such as pegloticase [15,16]. Reducing the sUA below the saturation point of MSU in body fluid can prevent the formation of new MSU crystals and lead to the dissolution of existing crystals. XOIs are used as first-line ULT, which are, nonetheless, associated with many severe adverse effects and low response rate; uricosuric agents are usually used as second-line therapy when patients are refractory to XOIs alone or contraindicated for them [13]. Uricases are only indicated for patients with severe gout or high sUA burden, such as those with tophaceous deformities, tumor lysis syndrome or Lesch-Nyhan syndrome [13].

The production of uric acid is balanced by its elimination, mainly in the urine. Approximately one-third of urate is excreted via the gastrointestinal tract, while the remaining two-thirds via the kidney [12]. In humans, sUA levels are controlled by a complex system of transporters expressed in the renal proximal tubule. Thus, uric acid is freely filtered at the glomerulus, and greater than 90% of the urate filtered in the kidney is reabsorbed back into the bloodstream with the remaining less than 10% being excreted in urine; this reabsorption process is mainly mediated by uric acid transporter 1 (URAT1, SLC22A12), also known as urate transporter 1 or urate-anion exchanger. URAT1, first identified in 2002, is a 12-transmembrane protein and prominently expressed on the apical side of epithelial cells of proximal tubules in the renal cortex [12,17]. In light of the fact that more than 90% of the patients with hyperuricemia are urate under-excretors and URAT1 is responsible for most of the uric acid reabsorption (about 90%), URAT1 inhibitors were believed to be a very efficacious and promising class of uricosuric agents for the treatment of hyperuricemia and gout [18,19].

**Figure 1:**
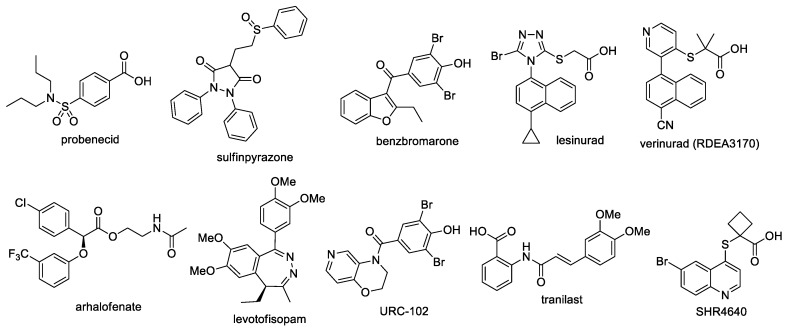
Structures of approved URAT1 inhibitors and selected URAT1 inhibitors under development in clinical trials.

**Figure 2:**
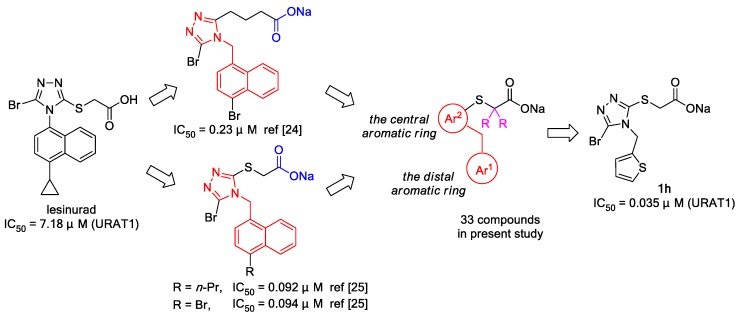
Design of URAT1 inhibitors in present study and discovery of **1h**.

**Scheme 1:**
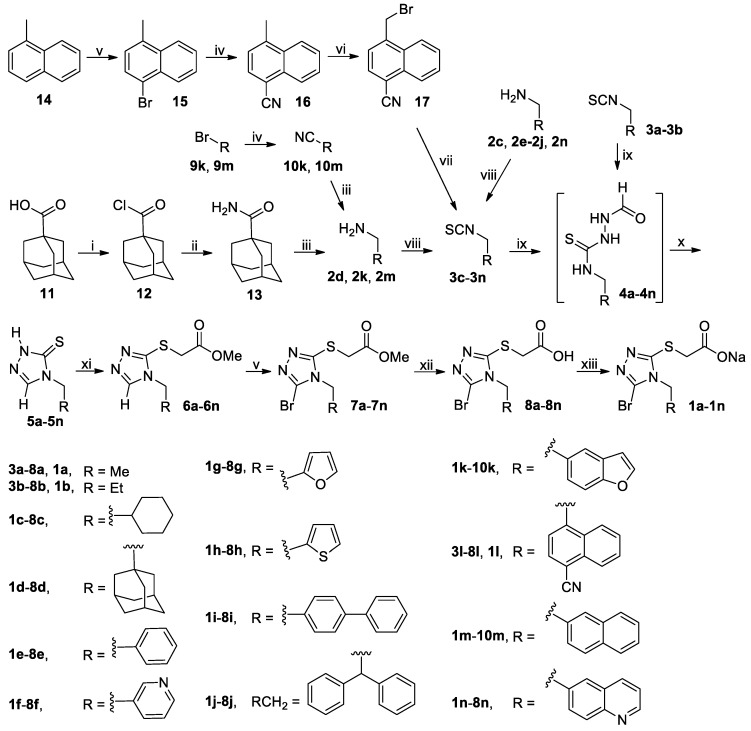
Synthetic route to **1a**–**1n**. *Reagents and conditions*: (i) SOCl_2_, DMF (cat.), dried CH_2_Cl_2_, 0 °C-reflux; (ii) NH_3_·H_2_O, THF, 0 °C-rt; (iii) LiAlH_4_, dried THF, 0 °C-reflux; (iv) CuCN, DMF, 130 °C (**15** and **9m**) or reflux (**9k**), N_2_; (v) for **14**: NBS, MeCN, 30–40 °C; for **6a**–**6c** and **6e**–**6n**: NBS, MeCN, rt; for **6d**: DBDMH, CH_2_Cl_2_, rt; (vi) NBS, BPO, CCl_4_, reflux, N_2_; (vii) KSCN, DMF, 140 °C, N_2_; (viii) CSCl_2_, DIPEA, dried CH_2_Cl_2_, 0 °C-rt; (ix) HCONHNH_2_, THF, rt ; (x) aq K_2_CO_3_, THF (**4a**–**4b**) or DMF (**4c**–**4n**), 50 °C; (xi) BrCH_2_CO_2_Me, K_2_CO_3_, acetone (**5a**–**5b**) or DMF (**5c**–**5n**), rt; (xii) aq LiOH, MeOH, rt; (xiii) aq NaOH, MeOH, rt.

**Scheme 2:**
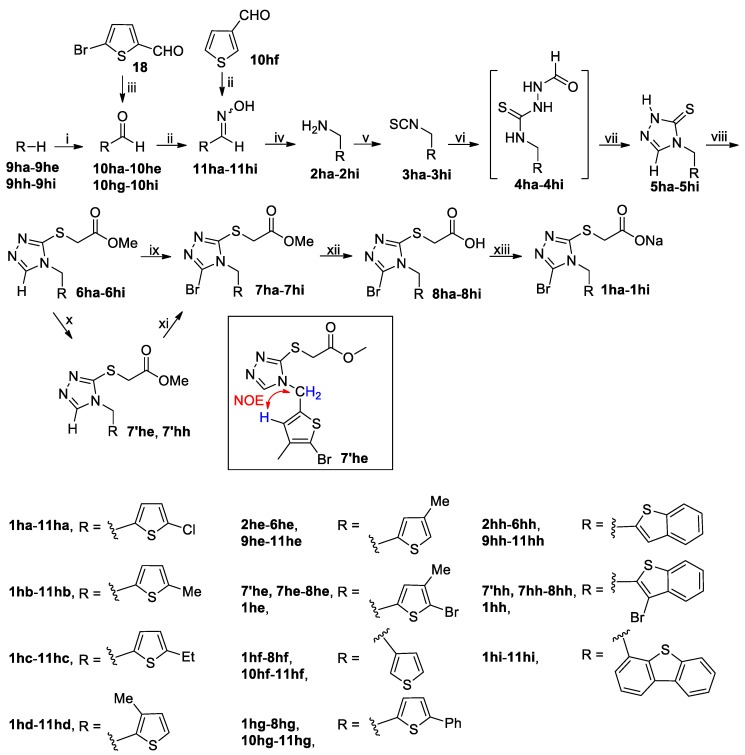
Synthetic route to **1ha**–**1hi**. *Reagents and conditions*: (i) for **9ha**–**9hd**: POCl_3_, dried DMF, 100 °C; for **9he**, **9hh** and **9hi**: *n*-BuLi, dried THF, –78 °C (**9he** and **9hh**) or −78 °C-rt (**9hi**), then dried DMF, −78 °C-rt; (ii) NH_2_OH·HCl, K_2_CO_3_, 90%EtOH, 0 °C-rt; (iii) PhB(OH)_2_, Pd(PPh_3_)_4_, K_2_CO_3_, dioxane/H_2_O (9/1 by *v*/*v*), N_2_, reflux; (iv) LiAlH_4_, dried THF, 0 °C-reflux; (v) CSCl_2_, DIPEA, dried CH_2_Cl_2_, 0 °C-rt; (vi) HCONHNH_2_, THF, rt ; (vii) aq K_2_CO_3_, DMF, 50 °C; (viii) BrCH_2_CO_2_Me, K_2_CO_3_, DMF, rt; (ix) for **6ha**–**6hd** and **6hf**–**6hg**: NBS , MeCN, 0 °C; for **6hi**: DBDMH, CH_2_Cl_2_, rt; (x) for **6he** and **6hh**: NBS, MeCN, 0 °C; (xi) NBS , MeCN, rt; (xii) aq LiOH, MeOH, rt; (xiii) aq NaOH, MeOH, rt.

**Scheme 3:**
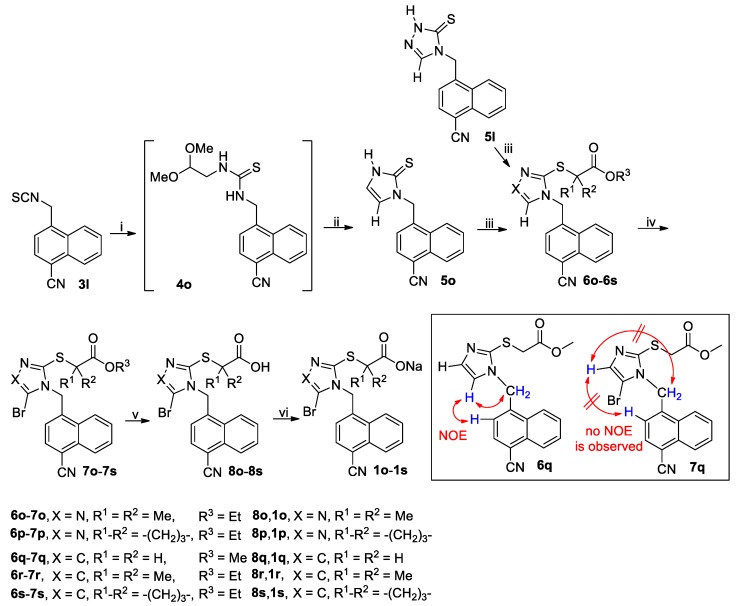
Synthetic route to **1o**–**1s**. *Reagents and conditions*: (i) NH_2_CH_2_CH(OMe)_2_, THF, rt; (ii) 25%H_2_SO_4_/AcOH (8/2 by *v*/*v*), reflux; (iii) for **6q**: BrCH_2_CO_2_Me, K_2_CO_3_, DMF, rt; for **6o** and **6r**: (CH_3_)_2_CBrCO_2_Et, K_2_CO_3_, DMF, 60 °C (**6o**) or rt (**6r**); for **6p** and **6s**, ethyl 1-bromocyclobutanecarboxylate, K_2_CO_3_, DMF, 80 °C (**6p**) or rt (**6s**); (iv) NBS, MeCN, 60 °C (**6o**), 45 °C (**6p**) or rt (**6q**–**6s**) ; (v) aq LiOH, MeOH, rt; (vi) aq NaOH, MeOH, rt.

**Scheme 4:**
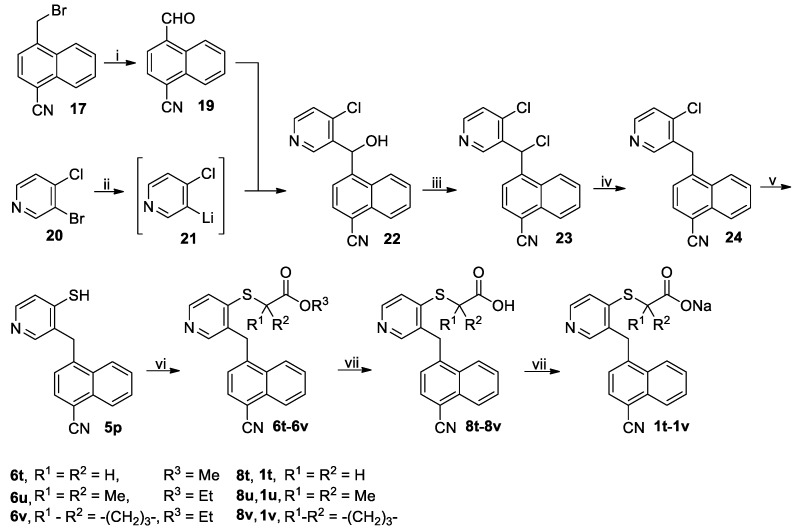
Synthetic route to **1t**–**1v**. *Reagents and conditions*: (i) (a) hexamethylenetetramine, 50%AcOH, reflux; (b) *con.* HCl, 50%AcOH, reflux; (ii) *n*-BuLi, dried THF, −78 °C, N_2_, then **19**, −78 °C-rt; (iii) SOCl_2_, dried CH_2_Cl_2_, 0 °C-rt; (iv) Zn, AcOH, rt; (v) Na_2_S·9H_2_O, DMF, 110 °C, N_2_; (vi) BrCH_2_CO_2_Me (**6t**), (CH_3_)_2_CBrCO_2_Et (**6u**) or ethyl 1-bromocyclobutanecarboxylate (**6v**), K_2_CO_3_, DMF, rt; (vii) aq LiOH, MeOH, rt; (viii) aq NaOH, MeOH, rt.

**Scheme 5:**
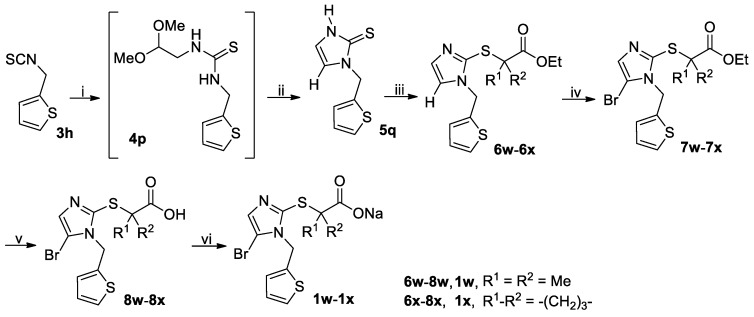
Synthetic route to **1w**–**1x**. *Reagents and conditions*: (i) NH_2_CH_2_CH(OMe)_2_, THF, rt; (ii) 25%H_2_SO_4_/AcOH (8/2 by *v*/*v*), reflux; (iii) for **6w**: (CH_3_)_2_CBrCO_2_Et, K_2_CO_3_, DMF, 60 °C; for **6x**: ethyl 1-bromocyclobutanecarboxylate, K_2_CO_3_, DMF, 40 °C; (iv) NBS, MeCN, 0 °C; (v) aq LiOH, MeOH, rt; (vi) aq NaOH, MeOH, rt.

Three uricosuric agents, probenecid, sulfinpyrazone and benzbromarone, were used in the clinic for a long time before the identification of URAT1 in 2002, and their uricosuric effect was found to arise from the URAT1 inhibitory activity (Figure 1) [17]. However, these agents suffer from a variety of adverse effects or drawbacks. Probenecid is associated with low efficacy, short half-life and significant drug-drug interaction (DDI) resulting from its dual inhibition against URAT1 and other organic anion transporters (OATs), such as OAT1 and OAT3 [12,20]. Sulfinpyrazone is also a very weak URAT1 inhibitor with a short half-life, and associated with severe gastrointestinal toxicity [21]. Benzbromarone is a potent URAT1 inhibitor, but notorious for severe hepatotoxicity, which resulted in its withdrawal from Europe in 2003 and non-approval in the US [22]. Lesinurad is a novel URAT1 inhibitor developed by Ardea Biosciences, now a subsidiary of AstraZeneca, and was approved by the U.S. Food and Drug Administration (FDA) in 2015 and by the European Medicines Agency (EMA) in 2016 for the treatment of hyperuricemia associated with gout in combination with an XOI. Lesinurad is the first URAT1 inhibitor approved after the identification of URAT1 in 2002; however, lesinurad suffers from low efficacy and a narrow therapeutic window [23]. Recently, considerable attention has been paid to the burden of gout on the individual and community as well as the discovery of URAT1 inhibitors on identification of URAT1 as a druggable therapeutic target, with lesinurad being approved and a number of others under development in clinical trials (Figure 1) [24,25,26,27,28,29]. In the effort to discover potent URAT1 inhibitors based on the structure of lesinurad in our laboratories, earlier studies successfully delivered a number of highly potent URAT1 inhibitors, and the IC_50_ values of some of them are even lower by almost two orders of magnitude than lesinurad (Figure 2) [30,31,32,33,34]. These highly potent URAT1 inhibitors share a common structural feature, i.e., a carboxylic group (in blue) connected to a flexible naphthyltriazolylmethane backbone (in red) by a linker. Encouraged by the earlier results, we moved forward to expand this specific naphthyltriazolylmethane backbone to a general diarylmethane backbone and carry out a systematic structure-activity relationship (SAR) study on the general backbone as well as the substituents on the α-position of the carboxylic acid group, with an expectation to better understand the SAR of this unique flexible structure and discover highly potent URAT1 inhibitors as promising candidate drugs (Scheme 1, Scheme 2, Scheme 3, Scheme 4 and Scheme 5). The SAR study delivered a highly potent URAT1 inhibitor **1h** with a new backbone, i.e., thiophenyltrizolylmethane, and an IC_50_ of 0.035 μM, which is the most potent URAT1 inhibitor discovered in our laboratories so far and also comparable to the most potent ones currently under development in clinical trials [12].

## 2. Results and Discussion

### 2.1. Chemistry

The synthetic route to target compounds **1a**–**1n** is shown in Scheme 1. 1-Adamantanecarboxylic acid **11** was treated with thionyl chloride in the presence of DMF as catalyst in dried CH_2_Cl_2_ at 0 °C to reflux temperature to afford the corresponding acyl chloride **12**, which was subsequently treated with aqueous ammonia in THF at 0 °C to room temperature to smoothly produce amide **13**. Reduction of amide **13** with LiAlH_4_ in dried THF at 0 °C to reflux temperature yielded corresponding methylamine **2d**. 5-Bromobenzo[b]furan **9k** and 2-bromonaphthalene **9m** reacted with CuCN in DMF at high temperatures (reflux for **9k** and 130 °C for **9m**) in N_2_ atmosphere to furnish **10k** and **10m**, respectively, which were then reduced with LiAlH_4_ in dried THF at 0 °C to reflux temperature to afford corresponding methylamines **2k** and **2m**, respectively. 1-Methylnaphthalene **14** was brominated with *N*-bromosuccinimide (NBS) in acetonitrile at 30–40 °C to afford 1-bromo-4-methylnaphthalene **15**, which was treated with CuCN in DMF in N_2_ atmosphere at 130 °C to give rise to cyanonaphthalene **16**. Methylnaphthalene **16** was smoothly converted to bromomethylnaphthalene **17** by treatment with NBS in the presence of benzoyl peroxide (BPO) in CCl_4_ at reflux in N_2_ atmosphere, which was subsequently treated with 3 eq of KSCN in DMF at 140 °C in N_2_ atmosphere to afford isothiocyanate **3l**. It is worth noting that the high reaction temperature is critically important for the selective formation of isothiocyanate **3l** over its potential isomer thiocyanate [35]. Methylamines **2c**–**2k** and **2m**–**2n** were treated with thiophosgene in the presence of diisopropylethylamine (DIPEA) as acid scavenger in dried CH_2_Cl_2_ at 0 °C to room temperature to yield corresponding isothiocyanates **3c**–**3k** and **3m**–**3n**, respectively. Isothiocyanates **3a**–**3n** reacted first with formic hydrazide in THF at room temperature to furnish the adducts **4a**–**4n**, which were in turn directly treated with aqueous K_2_CO_3_ in THF (**4a**–**4b**) or DMF (**4c**–**4n**) at 50 °C to effect the cyclization to give the triazole thiones **5a**–**5n** without further purification and characterization. Selective *S*-alkylation of thiones **5a**–**5n** with methyl bromoacetate in the presence of K_2_CO_3_ in acetone (**5a**–**5b**) or DMF (**5c**–**5n**) at room temperature gave esters **6a**–**6n**. Treatment of **6a**–**6c** and **6e**–**6n** with NBS in acetonitrile at room temperature smoothly effected selective bromination at 5-position of the triazole ring to successfully yield **7a**–**7c** and **7e**–**7n**, respectively; however, after considerable experimentation, the bromination of **6d** to **7d** could only be brought about with a more powerful brominating reagent 1,3-dibromo-5,5-dimethylhydantoin (DBDMH) in CH_2_Cl_2_ at room temperature presumably due to the steric hindrance posed by the bulky adamantane ring [36]. Alkaline hydrolysis of esters **7a**–**7n** with aqueous LiOH in MeOH at room temperature afforded corresponding carboxylic acids **8a**–**8n**, which were converted to sodium salts thereof for the sake of improving the aqueous solubility to facilitate the operation in in vitro assay. 

The synthetic route to target compounds **1ha**–**1hi** is summarized in Scheme 2. Standard Vilsmeier formylation at the α-position of thiophenes **9ha**–**9hc** with POCl_3_/DMF at 100 °C smoothly produced corresponding aldehydes **10ha**–**10hc** [37]; however, under the same reaction condition, 3-methylthiophene **9hd** gave an inseparable mixture of 3-methylthiophene-2-carbaldehyde **10hd** and 4-methylthiophene-2-carbaldehyde **10he** in a ratio of 85/15 as indicated by ^1^H-NMR because of the presence of two α-positions in **9hd** [38]. The minor isomer **10he** was removed from **10hd** by recrystallization of the mixture of their corresponding oximes **11hd** and **11he** in the next step. Compounds **9he**, **9hh** and **9hi** were first treated with *n*-BuLi in dried THF at −78 °C (**9he** and **9hh**) or at −78 °C to room temperature (**9hi**) to afford the corresponding aryl lithiums, which were in turn trapped with dried DMF at −78 °C to room temperature to produce aldehydes **10he**, **10hh** and **10hi**, respectively. It is worth noting that, as with in the case of the conversion of **9hd** to **10hd** and **10he** as described above, **9he** (identical to **9hd** actually) also gave an inseparable mixture of **10hd** and **10he** in a ratio of 19/81 under the second formylation condition [38], and the minor isomer **10hd** was also removed from **10he** by recrystallization of the mixture of corresponding oximes **11hd** and **11he** in the next step. Standard Suzuki coupling of **18** and PhB(OH)_2_ in the presence of Pd(PPh_3_)_4_ and K_2_CO_3_ in dioxane/H_2_O (9/1 by *v*/*v*) in N_2_ atmosphere at reflux smoothly afforded **10hg**. Aldehydes **10ha**–**10hi** were converted to corresponding oximes **11ha**–**11hi** each as a *Z*/*E* mixture by treatment with hydroxylamine hydrochloride in the presence of K_2_CO_3_ in 90% ethanol at 0 °C to room temperature. Oximes **11ha**–**11hi** were reduced to corresponding methylamines **2ha**–**2hi** with LiAlH_4_ in dried THF at 0 °C to reflux temperature. Following the same procedure for the synthesis of **1c**–**1n** from **2c**–**2n** in Scheme 1, **2ha**–**2hi** were converted to targets compounds **1ha**–**1hi**. However, there are some exceptions in the bromination of **6ha**–**6hi** that should be noted: (1) just as with the bromination of **6d** with a bulky adamantane ring, the bromination of **6hi** also needed the powerful brominating reagent DBDMH because of the presence of bulky dibenzothiophene ring; (2) unlike bromination of **6a**–**6c** and **6e**–**6n** with NBS that proceeded at room temperature (Scheme 1), the bromination of compounds **6ha**–**6hd** and **6hf**–**6hg** with NBS required to be carried out at 0 °C to suppress the potential bromination of the thiophene ring because thiophene ring is an aromatic ring more electron-rich than most of the counterparts in Scheme 1; (3) treatment of **6he** and **6hh** with 1 eq of NBS gave intermediates **7′he** and **7′hh**, respectively, even at 0 °C, where the bromine atom was introduced to the thiophene ring instead of the desired triazole ring because the unoccupied α-position in 4-methyl-2-thiophene ring and β-position in benzothiophene ring are more electron-rich and therefore more nucleophilic than the 5-position in triazole ring. Further treatment of **7′he** and **7′hh** with 1 eq of NBS produced **7he** and **7hh**, respectively, where the newly introduced bromine atom was at the desired 5-position of triazole ring. The exact position of the bromine atom in **7′he** was unambiguously elucidated by Nuclear Overhauser Effect (NOE) as shown in Scheme 2.

The synthetic route to **1o**–**1s** is depicted in Scheme 3. Isothiocyanate **3l** first reacted with 2-aminoacetaldehyde dimethyl acetal in THF at room temperature to give an adduct **4o**, which in turn underwent cyclization to afford imidazole thione **5o** by treatment in refluxing 25%H_2_SO_4_/AcOH (8/2 by *v*/*v*) [39]. Selective *S*-alkylation of imidazole thione **5o** with methyl bromoacetate, ethyl 2-bromo-2-methylpropionate and ethyl 1-bromocyclobutanecarboxylate in the presence of K_2_CO_3_ in DMF at room temperature afforded **6q**, **6r** and **6s**, respectively. Likewise, selective *S*-alkylation of triazole thione **5l** with ethyl 2-bromo-2-methylpropionate and ethyl 1-bromocyclobutanecarboxylate in the presence of K_2_CO_3_ at elevated temperatures (60 °C for **6o** and 80 °C for **6p**) yielded **6o** and **6p**, respectively. Compounds **6o**–**6s** were converted to target compounds **1o**–**1s** following the procedure used for the synthesis of **1a**–**1n** from **6a**–**6n** as shown in Scheme 1. Noteworthy is that the brominations of **6o** and **6p** were carried out at higher temperatures instead of room temperature, i.e., 60 °C for **6o** and 45 °C for **6p**. In the bromination of imidazole ring in **6q**–**6s**, there are two potential positions to be brominated, and the exact position at which the bromine atom was introduced was unequivocally determined by NOE as depicted in Scheme 3, which is also consistent with the reported results in similar situation [40]. The exact position of the bromine atom attached to the imidazole ring in **7q**–**7s** was identical to that in their triazole counterparts **1a**–**1n** and **1ha**–**1hi**, which is critically important in SAR study when to compare the biological activity of a pair of compounds with only the central rings being different while other positions including the substituents being completely identical.

The synthetic route to target compounds **1t**–**1v** is illustrated in Scheme 4. 4-Cyano-1-naphthaldehyde **19** was prepared from (4-cyano-1-naphthyl)methyl bromide **17** by standard Sommelet reaction [41]. Thus, **17** reacted first with hexamethylenetetramine in refluxing 50% aqueous acetic acid, and the quaternary ammonium intermediate thus formed was further hydrolyzed with hydrochloric acid to give rise to **19**. Treatment of 3-bromo-4-chloropyridine **20** with *n*-BuLi in dried THF at −78 °C furnished **21**, a reactive aryl lithium intermediate resulting from the selective Br-Li exchange [42], which was in turn trapped with aldehyde **19** to give **22**. Treatment of benzylic alcohol **22** with thionyl chloride in dried CH_2_Cl_2_ at 0 °C to room temperature gave benzylic chloride **23**, which was subsequently reduced with zinc powder in acetic acid at room temperature to smoothly produce **24** with a desired naphthylpyridinylmethane backbone [43]. Initial attempts to directly reduce benzylic alcohol **22** to **24** with Et_3_SiH/BF_3_·Et_2_O [44], NaBH_4_/AlCl_3_ [45] or 57%HI/AcOH [46] were unsuccessful because no reaction occurred for the first reagent, a side reaction of AlCl_3_-mediated dechlorination predominated for the second one and the reaction proceeded considerably slowly for the third one. Treatment of chloropyridine **24** with Na_2_S·9H_2_O in DMF at 110 °C in N_2_ atmosphere furnished pyridine thiol **5p**. Selective *S*-alkylation of thiol **5p** with methyl bromoacetate, ethyl 2-bromo-2-methylpropionate and ethyl 1-bromocyclobutanecarboxylate in the presence of K_2_CO_3_ in DMF at room temperature gave **6t**, **6u** and **6v**, respectively. Alkaline hydrolysis of **6t**–**6v** with aqueous LiOH in methanol at room temperature afforded corresponding acids **8t**–**8v**, which were then converted to sodium salts thereof **1t**–**1v** as described above.

The synthetic route to target compounds **1w**–**1x** is presented in Scheme 5. Imidazole thione **5q** was prepared from isothiocyanate **3h** following the procedure described above for the synthesis of **5o** from **3l**. Treatment of thione **5q** with ethyl 2-bromo-2-methylpropionate and ethyl 1-bromocyclobutanecarboxylate in the presence of K_2_CO_3_ in DMF at elevated temperatures (60 °C for **6w** and 40 °C for **6x**) produced **6w** and **6x**, respectively. Compounds **6w**–**6x** were converted to target compounds **1w**–**1x** following the procedure used for the synthesis of **1q**–**1s** from **6q**–**6s** shown in Scheme 3 except that the bromination of **6w**–**6x** with NBS was carried out at 0 °C in order to suppress the potential bromination of thiophene ring.

### 2.2. In Vitro URAT1 Inhibitory Activity

The results of in vitro *inhi*bitory assay of 33 synthesized compounds, i.e., **1a**–**1x** and **1ha**–**1hi**, as well as lesinurad as positive control against human URAT1 (Supplementary Material) are summarized in Table 1.

As shown in Figure 2, the diarylmethane backbone consists of two aromatic rings, i.e., the distal and central aromatic rings, corresponding to the modifications of the naphthalene and triazole rings in the original naphthyltriazolylmethane backbone, respectively. The SAR exploration of the diarylmethane backbone in present study started with the distal aromatic ring. This round of SAR study was divided into two phases: the first phase was mainly focused on the discovery of an optimal framework of the distal ring (**1a**–**1n**), and the second one was the fine-tuning of the substituents around the optimal framework identified in the first phase to further find its most preferred substituent (**1ha**–**1hi**). Thus, as shown from the results of **1a**–**1n**, the optimal framework for the distal ring is 2-thiophene ring with **1h** being the most potent URAT1 inhibitor among **1a**–**1n** (IC_50_ = 0.035 μM). It is also obvious that, with only a few exceptions (such as **1f** and **1a**), most compounds in **1a**–**1n** are more potent than lesinurad, but there is a trend that compounds with two aromatic rings in the R substituent (**1i**–**1n**) are generally more potent than those with only one aromatic or lower (cyclo)alkyl groups (**1a**–**1h**). Noteworthy is that although this trend didn’t deliver the most potent compound (**1h**) in this phase, it still warrants further study in the future. With 2-thiophene ring being identified as the optimal framework for the distal aromatic ring, we progressed to the second phase, i.e., fine-tuning of the substituents around 2-thiophene ring. Thus, among **1ha**–**1hi** with a 2-thiophene ring and its close analogues, no compound was found to be more potent than **1h**, indicating that the framework of 2-thiophene cannot tolerate any substituent. It is interesting that 3-thiophene ring, a close analogue of 2-thiophene ring, led to the complete loss of URAT1 inhibitory activity (**1hf**). Compound **1hh** with a 3-bromobenzo[b]thiophene ring exhibited highly potent URAT1 inhibitory activity (IC_50_ = 0.12 μM), which, however, is still less potent than **1h**.

In order to accelerate the whole process of SAR exploration, we carried out the SAR exploration of the central aromatic ring as well as the substituents on the α-position of the carboxylic acid group *at the same time* we performed the first round of SAR study. In the second round, we kept the distal aromatic ring as 4-cyano-1-naphthalene ring and designed 9 compounds (**1o**–**1v**). Thus, it is clear that for all the three central aromatic rings, i.e., 1,2,4-triazole, imidazole and pyridine, the activity was uniformly enhanced dramatically when the size of α-substituents of the carboxylic acid group increased (**1p** > **1o** > **1l**; **1r** > **1s** > **1q**; **1v** > **1u** > **1t**), indicating that the *gem*-dimethyl and cyclobutane are optimal substituents at the α-position of the carboxylic acid group. Among the three central rings, the imidazole ring seemed the optimal one (**1r** > **1u** > **1o**; **1s** > **1p** > **1v**).

With in hand the optimal distal ring being 2-thiophene from the first-round SAR study and the optimal central ring and the α-substituent of the carboxylic acid group being imidazole ring and *gem*-dimethyl/cyclobutane, respectively, from the second-round, we advanced to the third round by merging the results from the first two rounds of SAR study and designed compounds **1w** and **1x** to see if there is an additive effect. It turned out that the URAT1 inhibitory activity of **1w** was weak but the **1x** was highly potent, which was, however, still less potent than **1h**.

In summary, **1h** displayed an IC_50_ of 0.035 μM, which is 200- and 8-fold more potent than the parent lesinurad and benzbromarone, respectively, making it the most potent URAT1 inhibitor discovered in our laboratories so far and also comparable to the most potent ones currently under development in clinical trials [18]. The present study strongly suggested that diarylmethane backbone is a considerably promising molecular scaffold for the design of potent URAT1 inhibitors.

## 3. Experimental Section

### 3.1. General

Melting points were measured with an RY-2 microscopic melting point apparatus (Tianjin Tianguang Optical Instrument Ltd., Tianjin, China) and are uncorrected. ^1^H-NMR and ^13^C-NMR spectra were recorded on a Bruker AV400 NMR spectrometer (Bruker BioSpin AG, Faellanden, Switzerland) using DMSO-*d*_6_, CDCl_3_ and MeOH-*d*_4_ as solvent and known chemical shifts of residual proton signals of deuterated solvents (for ^1^H-NMR) or carbon signals of deuterated solvents (for ^13^C-NMR) as internal standard. High-resolution mass spectra (HRMS) were recorded with an Agilent Q-TOF 6510 mass spectrometer (Agilent Technologies, Santa Clara, CA, USA) using the direct injection method and electrospray ionization (ESI) technique in negative mode (ESI–). HPLC purities are determined on a reverse-phase C_18_ column (Waters Atlantis T3, 5 μm, 4.6 × 150 mm; column temperature, 35 °C) at 225 nm using an Agilent 1260 VWD HPLC system with flow rate of 1.0 mL/min. Mobile phase A was MeCN/MeOH (90/10 by *v*/*v*), and mobile phase B was 10 mM aqueous (NH_4_)_2_HPO_4_ (adjusted to pH of 4.3 ± 0.03 with H_3_PO_4_). The gradient system started from A/B (25%/75%) at 0 min to A/B (40%/60%) at 30 min, A/B (50%/50%) at 45 min, A/B (70%/30%) at 60 min, A/B (70%/30%) at 70 min, A/B (25%/75%) at 71 min, and finally A/B (25%/75%) at 75 min.

Starting materials **11**, **9k**, **9m**, **14**, **2c**, **2e**–**2j**, **2n**, **3a**–**3b**, **9ha**–**9hd**, **9hh**–**9hi**, **18**, **10hf** and **20** were commercially available. All the dried solvents were prepared by standard methods. 

### 3.2. Chemistry

#### 3.2.1. General Procedure for the Synthesis of Substituted Methylamines **2d**, **2k** and **2m**

To a stirred mixture of 1-adamantanecarboxylic acid (**11**, 36.05 g, 200 mmol) and DMF (5 drops) in CH_2_Cl_2_ (360 mL) cooled in an ice-water bath was added thionyl chloride (26.17 g, 220 mmol) in one portion. The resulting mixture was refluxed until the evolution of HCl gas ceased (typically within 2 h). The solvent and excess thionyl chloride were then removed on a rotary evaporator, and the crude acid chloride **12** was dissolved in THF (100 mL). The resulting solution was then slowly added dropwise to stirred concentrated aqueous ammonia (400 mL) cooled in an ice-water bath. After addition, the resulting white slurry was stirred at room temperature for another 2 h, and the white precipitates were collected via vacuum filtration, washed with cold *n*-hexane and dried in vacuo to afford 1-adamantanecarboxamide (**13**). White solid; 34.06 g (95%); m.p. 177–179 °C (literature value, 185.5–191.7 °C [48]). ^1^H-NMR (DMSO-*d*_6_, 400 MHz) δ: 6.89 (brs, 1H), 6.62 (brs, 1H), 1.93 (s, 3H), 1.73–1.74 (m, 6H), 1.60–1.68 (m, 6H).

A mixture of 1-methylnaphthalene (**14**, 28.44 g, 200 mmol) and NBS (39.16 g, 220 mmol) in MeCN (300 mL) was stirred at 30-40°C until completion of the reaction as indicated by TLC analysis (typically within 12 h). On cooling to room temperature, the reaction mixture was poured into ice-water (600 mL), and the resulting mixture was extracted with CH_2_Cl_2_ (500 mL × 3). The combined extracts were washed successively with 5% aqueous Na_2_S_2_O_3_ (200 mL), saturated aqueous Na_2_CO_3_ (100 mL × 3) and 5% brine (200 mL), dried (Na_2_SO_4_) and evaporated on a rotary evaporator to give a residue, which was purified by column chromatography to afford 1-bromo-4-methylnaphthalene (**15**)**.** Colorless oil, 42.01 g (98%). ^1^H-NMR (DMSO-*d*_6_, 400 MHz) δ: 8.14–8.16 (m, 1H), 8.07–8.09 (m, 1H), 7.76 (d, *J =* 7.6 Hz, 1H), 7.66–7.70 (m, 2H), 7.30 (d, *J =* 7.6 Hz, 1H), 2.63 (s, 3H). 

A mixture of **15**, **9k** or **9m** (190 mmol) and CuCN (49.00 g, 570 mmol) in DMF (500 mL) were stirred at 130 °C (for **15** and **9m**) or reflux (for **9k**) in N_2_ atmosphere for 12 h, when TLC analysis indicated completion of reaction. On cooling to room temperature, the reaction mixture was diluted with CH_2_Cl_2_ (1000 mL), and the resulting mixture was further stirred for 1 h and filtered off. The filtrate was washed with 5% brine (500 mL × 5), dried (Na_2_SO_4_) and evaporated on a rotary evaporator, which was purified by column chromatography to afford **16**, **10k** or **10m**.

*4-Methyl-1-naphthonitrile* (**16**): White solid; 24.78 g (78%); m.p. 54–55 °C (literature value, 53–54 °C [49]). ^1^H-NMR (DMSO-*d*_6_, 400 MHz) δ: 8.20 (d, *J =* 8.0 Hz, 1H), 8.11 (d, *J =* 8.0 Hz, 1H), 8.04 (d, *J =* 7.2 Hz, 1H), 7.72–7.82 (m, 2H), 7.53 (d, *J =* 7.2 Hz, 1H), 2.74 (s, 3H).

*Benzo[b]furan-5-carbonitrile* (**10k**): White solid; 23.12 g (85%); m.p. 81–83 °C (literature value, 81–82 °C [50]). ^1^H-NMR (DMSO-*d*_6_, 400 MHz) δ: 8.22 (d, *J =* 1.2 Hz, 1H), 8.19 (d, *J =* 2.0 Hz, 1H), 7.81 (d, *J =* 8.4 Hz, 1H), 7.73–7.75 (m, 1H), 7.08 (d, *J =* 1.6 Hz, 1H).

*2-Naphthonitrile* (**10m**): White solid; 20.96 g (72%); m.p. 63–65 °C (literature value, 65–66 °C [51]). ^1^H-NMR (DMSO-*d*_6_, 400 MHz) δ: 8.57 (s, 1H), 8.03–8.11 (m, 3H), 7.65–7.78 (m, 3H).

To a stirred solution of **13**, **10k** or **10m** (130 mmol) in dried THF (150 mL) cooled in an ice-water bath was added portionwise LiAlH_4_ (6.41 g, 169 mmol). Thereafter the reaction mixture was stirred at room temperature for 1 h and then at reflux for another 5 h, when the reaction completed as indicated by TLC analysis. On cooling to room temperature, the reaction mixture was carefully poured into ice-water (500 mL) while stirring, and the resulting mixture was diluted with CH_2_Cl_2_ (300 mL), stirred for 0.5 h and filtered off through Celite. The organic phase was separated from the filtrate, and the aqueous phase was back-extracted with CH_2_Cl_2_ (200 mL × 2). The combined organic phases were washed with saturated brine (200 mL), dried (Na_2_SO_4_) and evaporated on a rotary evaporator to give a residue, which was purified by column chromatography through a short silica gel column to afford **2d**, **2k** or **2m**. These amines were used directly in the next step without further purification and characterization.

#### 3.2.2. General Procedure for the Synthesis of Substituted Methylamines **2ha**–**2hi**

To a stirred solution of 5-bromothiophene-2-carbaldehyde (**18**, 38.22 g, 200 mmol) and phenylboronic acid (26.82 g, 220 mmol) in dioxane/H_2_O (9/1 by *v*/*v*, 600 mL in total), K_2_CO_3_ (110.56 g, 800 mmol) and Pd(PPh_3_)_4_ (11.56 g, 10 mmol) were added. The reaction mixture was heated at reflux for 10 h in N_2_ atmosphere. On cooling to room temperature, the reaction mixture was filtered off. The organic phase was evaporated on a rotary evaporator, and the residue thus obtained was extracted with CH_2_Cl_2_ (200 mL × 3). The combined extracts were washed with 5% brine (200 mL), dried (Na_2_SO_4_) and evaporated on a rotary evaporator to give a residue, which was purified by column chromatography to afford 5-phenylthiophene-2-carbaldehyde (**10hg**). White solid; 36.90 g (98%); m.p. 89–91 °C (literature value, 92–93 °C [52]). ^1^H-NMR (DMSO-*d*_6_, 400 MHz) δ: 9.91 (s, 1H), 8.04 (d, *J =* 4.0 Hz, 1H), 7.79–7.81 (m, 2H), 7.74 (d, *J =* 4.0 Hz, 1H), 7.41–7.50 (m, 3H).

To a stirred solution of **9ha**–**9hd** (200 mmol) in dried DMF (43.86 g, 600 mmol) cooled in an ice-water bath was added dropwise POCl_3_ (46.00 g, 300 mmol). The resulting mixture was stirred at this temperature for 30 min and then at 100 °C for another 5 h. After cooling to room temperature, the reaction mixture was poured carefully into ice-water (300 mL). The mixture thus obtained was extracted with CH_2_Cl_2_ (300 mL × 3), and the combined extracts were washed successively with 5% brine (200 mL), 10% aqueous K_2_CO_3_ (200 mL) and 5% brine (200 mL), dried (Na_2_SO_4_) and evaporated on a rotary evaporator to afford a residue, which was purified by column chromatography to yield **10ha**–**10hd.**

*5-Chlorothiophene-2-carbaldehyde* (**10ha**): Colorless oil; 22.00 g (75%). ^1^H-NMR (DMSO-*d*_6_, 400 MHz) δ: 9.82 (s, 1H), 7.95 (d, *J =* 4.0 Hz, 1H), 7.39 (d, *J =* 4.0 Hz, 1H). The ^1^H-NMR data were in good agreement with those reported [53].

*5-Methylthiophene-2-carbaldehyde* (**10hb**): Colorless oil; 21.20 g (84%). ^1^H-NMR (DMSO-*d*_6_, 400 MHz) δ: 9.80 (s, 1H), 7.84 (d, *J =* 4.0 Hz, 1H), 7.04–7.05 (m, 1H), 2.54 (s, 3H). The ^1^H-NMR data were in good agreement with those reported [53].

*5-Ethylthiophene-2-carbaldehyde* (**10hc**): Colorless oil; 22.72 g (81%). ^1^H-NMR (DMSO-*d*_6_, 400 MHz), δ: 9.81 (s, 1H), 7.85 (d, *J =* 3.6 Hz, 1H), 7.06–7.07 (m, 1H), 2.88 (q, *J =* 7.5 Hz, 2H), 1.25 (t, *J =* 7.4 Hz, 3H). 

*3-Methylthiophene-2-carbaldehyde* (**10hd**): Colorless oil; 22.46 g (89%). An inseparable mixture of regioisomers as indicated by ^1^H-NMR. The ^1^H-NMR data were in good agreement with those reported [38].

To a stirred solution of **9he**, **9hh** or **9hi** (200 mmol) in dried THF (200 mL) held at −78 °C in N_2_ atmosphere was added dropwise 1.6 M *n*-BuLi in *n*-hexane (125 mL, 200 mmol) via syringe, and the resulting solution was stirred at −78 °C for 1 h ( for **9he** and **9hh**) or returned to room temperature for 5 h (for **9hi**) before dropwise addition of dried DMF (21.93 g, 300 mmol) via syringe. Thereafter the reaction mixture was stirred at −78 °C for 0.5 h and at room temperature for another 1 h. The reaction mixture was poured into ice-water (600 mL) while stirring and the resulting mixture was extracted with CH_2_Cl_2_ (300 mL × 3). The combined extracts were washed with 5% brine (500 mL), dried (Na_2_SO_4_) and evaporated on a rotary evaporator to give a residue, which was purified by column chromatography to afford the pure product **10he**, **10hh** or **10hi.**

*4-Methylthiophene-2-carbaldehyde* (**10he**): Colorless oil; 23.97 g (95%). An inseparable mixture of regioisomers as indicated by ^1^H-NMR. The ^1^H-NMR data were in good agreement with those reported [38].

*Benzo[b]thiophene-2-carbaldehyde* (**10hh**): Colorless oil; 29.80 g (92%). ^1^H-NMR (DMSO-*d*_6_, 400 MHz) δ: 10.14 (s, 1H), 8.43 (s, 1H), 8.09–8.12 (m, 2H), 7.56–7.60 (m, 1H), 7.48–7.52 (m, 1H). The ^1^H-NMR data were in good agreement with those reported [54].

*Bibenzo[b,d]thiophene-4-carbaldehyde* (**10hi**): White solid; 36.08 g (85%); m.p. 117–119 °C. ^1^H-NMR (CDCl_3_, 400 MHz) δ: 10.27 (s, 1H), 8.40 (d, *J =* 7.6 Hz, 1H), 8.17–8.22 (m, 1H), 7.93–7.97 (m, 2H), 7.64 (t, *J =* 7.6 Hz, 1H), 7.47–7.54 (m, 2H). The ^1^H-NMR data were in good agreement with those reported [55].

To a stirred solution of **10ha**–**10hi** (145 mmol) and NH_2_OH·HCl (20.15 g, 290 mmol) in 90% EtOH (300 mL) was added dropwise an aqueous solution prepared by dissolving K_2_CO_3_ (24.05 g, 174 mmol) in a minimal amount of water. After addition, the mixture was stirred at room temperature until the reaction completed as indicated by TLC analysis (typically within 5 h). The reaction mixture was concentrated to about one-third of its original volume on a rotary evaporator and the residue was poured into ice-water (400 mL). The resulting mixture was adjusted to pH 6–7 with diluted hydrochloric acid and extracted with CH_2_Cl_2_ (200 mL × 3). The combined extracts were washed with 5% brine (200 mL), dried (Na_2_SO_4_) and evaporated on a rotary evaporator to give a residue, which was purified by recrystallization from *n*-hexane/EtOAc (5/1 by *v*/*v*) to give **11ha**–**11hi**. Oximes **11ha**–**11hi** were used directly in the next step without further characterization. 

The synthesis of **2ha**–**2hi** from **11ha**–**11hi** by the reduction with LiAlH_4_ was carried out using the identical operation to that for the synthesis of **2d**, **2k** and **2m** from **13**, **10k** and **10m**, respectively.

#### 3.2.3. General Procedure for the Synthesis of Isothiocyanates **3c**–**3n** and **3ha**–**3hi**

A suspension of **16** (23.40 g, 140 mmol), BPO (5.09 g, 21 mmol) and NBS (29.90 g, 168 mmol) in CCl_4_ (400 mL) was stirred at reflux in N_2_ atmosphere until the completion of reaction as indicated by TLC analysis (typically within 12 h; once the reaction commenced, 2.55 g of BPO was added every 5 h until the reaction completed). The reaction mixture was cooled to room temperature and filtered off, and the filtrate was successively washed with saturated aqueous NaHCO_3_ (200 mL × 5) and 5% brine (200 mL), dried (Na_2_SO_4_) and evaporated on a rotary evaporator to afford a residue, which was purified by column chromatography to yield (4-cyano-1-naphthyl)methyl bromide (**17**). White solid; 31.35 g (91%); m.p. 113–115 °C. ^1^H-NMR (DMSO-*d*_6_, 400 MHz) δ: 8.34–8.37 (m, 1H), 8.13–8.21 (m, 2H), 7.82–7.89 (m, 3H), 5.27 (s, 2H).

To a stirred solution of **17** (30.76 g, 125 mmol) in DMF (300 mL) was added KSCN (24.30 g, 250 mmol), and the mixture thus obtained was stirred for 4 h at 140 °C in N_2_ atmosphere until the completion of reaction as indicated by TLC analysis. On cooling to room temperature, the reaction mixture was poured into ice-water (600 mL) while stirring. The resulting mixture was exacted with CH_2_Cl_2_ (200 mL × 3). The combined extracts were washed with 5% brine (300 mL), dried (Na_2_SO_4_) and evaporated on a rotary evaporator to give a residue, which was purified by column chromatography to afford (4-cyano-1-naphthyl)methyl isothiocyanate (**3l**). White solid; 20.47 g (73%); m.p. 113–115 °C. ^1^H-NMR (DMSO-*d*_6_, 400 MHz) δ: 8.18–8.22 (m, 3H), 7.81–7.89 (m, 2H), 7.75 (d, *J =* 7.6 Hz, 1H), 5.56 (s, 2H).

To a stirred solution of thiophosgene (15.18 g, 132 mmol) in dried CH_2_Cl_2_ (150 mL) cooled in an ice-water bath was added dropwise a solution prepared by dissolving **2c**–**2k**, **2m**–**2n** or **2ha**–**2hi** (120 mmol) and DIPEA (46.53 g, 360 mmol) in dried CH_2_Cl_2_ (150 mL). The resulting mixture was stirred for 1 h in an ice-water bath and for another 1 h at room temperature. The reaction mixture was then poured into ice-water (300 mL) while stirring. The organic phase was separated, and the aqueous phase was back-extracted with CH_2_Cl_2_ (200 mL × 2). The combined organic phases were washed successively with 5% hydrochloric acid (100 mL × 2; for **3f** and **3n**, the washing with 5% hydrochloric acid is omitted) and saturated brine (300 mL), dried (Na_2_SO_4_) and evaporated on a rotary evaporator to give a residue, which was purified by column chromatography to afford **3c**–**3k**, **3m**–**3n** or **3ha**–**3hi**.

*Cyclohexylmethyl isothiocyanate* (**3c**): Colorless oil; 18.26 g (98%). ^1^H-NMR (DMSO-*d*_6_, 400 MHz) δ: 3.51 (d, *J =* 6.4 Hz, 2H), 1.60–1.70 (m, 6H), 1.17–1.27 (m, 2H), 1.08–1.14 (m, 1H), 0.93–1.03 (m, 2H). The ^1^H-NMR data were in good agreement with those reported [56].

*1-Adamantylmethyl isothiocyanate* (**3d**): White solid; 22.89 g (92%); m.p. 76–77 °C. ^1^H-NMR (DMSO-*d*_6_, 400 MHz) δ: 3.33 (s, 2H), 1.98 (s, 3H), 1.67–1.70 (m, 3H), 1.57–1.60 (m, 3H), 1.50–1.51 (m, 6H).

*Benzyl isothiocyanate* (**3e**): Colorless oil; 15.94 g (89%). ^1^H-NMR (DMSO-*d*_6_, 400 MHz) δ: 7.34–7.44 (m, 5H), 4.93 (s, 2H). The ^1^H-NMR data were in good agreement with those reported [57].

*3-Pyridinylmethyl isothiocyanate* (**3f**): Colorless oil; 10.81 g (ca 61%). ^1^H-NMR (DMSO-*d*_6_, 400 MHz) δ: 8.59 (d, *J =* 1.6 Hz, 1H), 8.56 (d, *J =* 1.2 Hz and 4.8 Hz, 1H), 7.82 (d, *J =* 7.6 Hz, 1H), 7.45 (dd, *J =* 4.8 Hz and 8.0 Hz, 1H), 4.99 (s, 2H). The ^1^H-NMR data were in good agreement with those reported [56].

*2-Furanylmethyl isothiocyanate* (**3g**): Colorless oil; 15.87g (95%). ^1^H-NMR (DMSO-*d*_6_, 400 MHz) δ: 7.71 (s, 1H), 6.46–6.47 (m, 2H), 4.95 (s, 2H). The ^1^H-NMR data were in good agreement with those reported [56].

*2-Thiophenylmethyl isothiocyanate* (**3h**): Colorless oil; 16.39 g (88%). ^1^H-NMR (DMSO-*d*_6_, 400 MHz) δ: 7.56 (dd, *J =* 1.2 Hz and 5.2 Hz, 1H), 7.14 (d, *J =* 3.2 Hz, 1H), 7.03 (dd, *J =* 3.6 Hz and 5.2 Hz, 1H), 5.12 (s, 2H). 

*Biphenyl-4-methyl isothiocyanate* (**3i**): White solid; 23.25 g (86%); m.p. 66–67 °C. ^1^H-NMR (DMSO-*d*_6_, 400 MHz) δ: 7.66–7.72 (m, 4H), 7.45–7.48 (m, 4H), 7.35–7.39 (m, 1H), 4.97 (s, 2H). The ^1^H-NMR data were in good agreement with those reported [56].

*Diphenylmethyl isothiocyanate* (**3j**): Colorless oil; 22.17 g (82%). ^1^H-NMR (DMSO-*d*_6_, 400 MHz) δ: 7.31–7.43 (m, 10H), 6.50 (s, 1H). The ^1^H-NMR data were in good agreement with those reported [58].

*Benzo[b]furan-5-methyl isothiocyanate* (**3k**): Colorless oil; 20.21 g (89%). ^1^H-NMR (DMSO-*d*_6_, 400 MHz) δ: 8.03 (d, *J =* 2.4 Hz, 1H), 7.68 (d, *J =* 1.2 Hz, 1H), 7.63 (d, *J =* 8.4 Hz, 1H), 7.33 (dd, *J =* 2.0 Hz and 8.4 Hz, 1H), 7.00 (d, *J =* 0.8 Hz and 2.4 Hz, 1H), 5.00 (s, 2H). 

*2-Naphthylmethyl isothiocyanate* (**3m**): White solid; 20.33 g (85%); m.p. 62–63 °C. ^1^H-NMR (DMSO-*d*_6_, 400 MHz) δ: 7.90–7.98 (m, 4H), 7.49–7.57 (m, 3H), 5.11 (s, 2H).

*Quinoline-6-methyl isothiocyanate* (**3n**): White solid; 18.74 g (78%); m.p. 186–188 °C. ^1^H-NMR (DMSO-*d*_6_, 400 MHz) δ: 9.22 (dd, *J =* 1.2 Hz and 8.8 Hz, 1H), 9.03 (d, *J =* 8.4 Hz, 1H), 8.38 (d, *J =* 8.8 Hz, 1H), 8.27 (s, 1H), 8.03 (dd, *J =* 1.6 Hz and 8.8 Hz, 1H), 7.98 (dd, *J =* 5.2 Hz and 8.4 Hz, 1H), 5.28 (s, 2H).

*(5-Chloro-2-thiophenyl)methyl isothiocyanate* (**3ha**): Colorless oil; 19.80 g (87%). ^1^H-NMR (DMSO-*d*_6_, 400 MHz) δ: 7.03–7.05 (m, 2H), 5.06 (s, 2H). 

*(5-Methyl-2-thiophenyl)methyl isothiocyanate* (**3hb**): Colorless oil; 16.66 g (82%). ^1^H-NMR (DMSO-*d*_6_, 400 MHz) δ: 6.92 (d, *J =* 3.6 Hz, 1H), 6.69–6.70 (m, 1H), 5.01 (s, 2H), 2.43 (s, 3H). 

*(5-Ethyl-2-thiophenyl)methyl isothiocyanate* (**3hc**): Colorless oil; 18.69 g (92%). ^1^H-NMR (DMSO-*d*_6_, 400 MHz) δ: 6.93 (d, *J =* 3.6 Hz, 1H), 6.73 (d, *J =* 3.6 Hz, 1H), 5.02 (s, 2H), 2.78 (q, *J =* 7.5 Hz, 2H), 1.22 (t, *J =* 7.6 Hz, 3H). 

*(3-Methyl-2-thiophenyl)methyl isothiocyanate* (**3hd**): Colorless oil; 11.98 g (59%). ^1^H-NMR (DMSO-*d*_6_, 400 MHz) δ: 7.43 (d, *J =* 5.2 Hz, 1H), 6.91 (d, *J =* 4.8 Hz, 1H), 5.03 (s, 2H), 2.20 (s, 3H). 

*(4-Methyl-2-thiophenyl)methyl isothiocyanate* (**3he**): Colorless oil; 10.56 g (52%). ^1^H-NMR (DMSO-*d*_6_, 400 MHz) δ: 7.12 (s, 1H), 6.95 (s, 1H), 5.05 (s, 2H), 2.18 (s, 3H). 

*(3-Thiophenyl)methyl isothiocyanate* (**3hf**) : Colorless oil; 15.46 g (83%). ^1^H-NMR (DMSO-*d*_6_, 400 MHz) δ: 7.58–7.60 (m, 1H), 7.53 (d, *J =* 2.0 Hz, 1H), 7.13 (dd, *J =* 0.8 Hz and 5.2 Hz, 1H), 4.90 (s, 2H). *(5-Phenyl-2-thiophenyl)methyl isothiocyanate* (**3hg**): White solid; 26.37 g (95%); m.p. 63–65 °C. ^1^H-NMR (DMSO-*d*_6_, 400 MHz) δ: 7.63–7.65 (m, 2H), 7.40–7.44 (m, 3H), 7.30–7.34 (m, 1H), 7.15 (d, *J =* 4.0 Hz, 1H), 5.14 (s, 2H).

*(Benzo[b]thiophen-2-yl)methyl isothiocyanate* (**3hh**): White solid; 23.65 g (96%); m.p. 62–64 °C. ^1^H-NMR (DMSO-*d*_6_, 400 MHz) δ: 7.96–7.98 (m, 1H), 7.84–7.86 (m, 1H), 7.46 (s, 1H), 7.35–7.41 (m, 2H), 5.25 (s, 2H).

*(Dibenzo[b,d]thiophen-4-yl)methyl isothiocyanate* (**3hi**): White solid; 28.19 g (92%); m.p. 81–83 °C. ^1^H-NMR (DMSO-*d*_6_, 400 MHz) δ: 8.39–8.42 (m, 2H), 8.09–8.11 (m, 1H), 7.53–7.60 (m, 4H), 5.24 (s, 2H).

#### 3.2.4. General Procedure for the Synthesis of Thiones **5a**–**5n** and **5ha**–**5hi**

A mixture of isothiocyanate **3a**–**3n** or **3ha**–**3hi** (90 mmol) and formic hydrazide (6.49 g, 108 mmol) in THF (150 mL) was stirred at room temperature until the reaction completed as indicated by TLC analysis, when a white slurry formed. The crystals were collected by vacuum filtration and dried in vacuo at room temperature to give the intermediate **4a**–**4n** or **4ha**–**4hi**, which was used directly in the next step without further purification and characterization. Thus, **4a**–**4n** or **4ha**–**4hi** was dissolved in THF (150 mL; for **4a**–**4b**) or DMF (150 mL; for **4c**–**4n** and **4ha**–**4hi**) followed by addition of aqueous K_2_CO_3_ prepared by dissolving K_2_CO_3_ (12.44 g, 90 mmol) in a minimal amount of water, and the solution thus obtained was stirred at 50 °C until completion of the reaction as indicated by TLC analysis (typically within 5 h). On cooling to room temperature, for **4a**–**4b**: the reaction mixture was directly concentrated on a rotary evaporator to remove THF, and the aqueous residue was adjusted to pH 5–6 and further evaporated to dryness to give a residue, which was purified by column chromatography to afford **5a**–**5b**; for **4c**–**4n** or **4ha**–**4hi**: the reaction mixture was poured into ice-water (300 mL), and the resulting mixture was adjusted to pH 5–6 with diluted hydrochloric acid to give a white slurry. The white crystals were collected by vacuum filtration, washed with water and dried in vacuo to give the crude product **5c**–**5n** or **5ha**–**5hi** as a white solid, which was triturated with EtOAc/*n*-hexane to yield the pure product **5c**–**5n** or **5ha**–**5hi**.

*4-Ethyl-2,3-dihydro-4H-1,2,4-triazoline-3-thione* (**5a**): White solid; 8.08 g (78%); m.p. 169–171 °C. ^1^H-NMR (DMSO-*d*_6_, 400 MHz) δ: 13.61 (brs, 1H), 8.38 (s, 1H), 3.42 (s, 3H). ^13^C-NMR (DMSO-*d*_6_, 100 MHz) δ: 166.36, 142.60, 31.27.

*4-n-Propyl-2,3-dihydro-4H-1,2,4-triazoline-3-thione* (**5b**): White solid; 8.49 g (73%); m.p. 90–91 °C. ^1^H-NMR (DMSO-*d*_6_, 400 MHz) δ: 13.63 (brs, 1H), 8.45 (s, 1H), 3.91 (q, *J =* 7.2 Hz, 2H), 1.26 (t, *J =* 7.2 Hz, 3H). ^13^C-NMR (DMSO-*d*_6_, 100 MHz) δ: 165.57, 141.53, 39.32, 13.92.

*4-(Cyclohexylmethyl)-2,3-dihydro-4H-1,2,4-triazoline-3-thione* (**5c**): White solid; 11.90 g (67%); m.p. 138–140 °C. ^1^H-NMR (DMSO-*d*_6_, 400 MHz) δ: 13.64 (brs, 1H), 8.42 (d, *J =* 1.2 Hz, 1H), 3.75 (d, *J =* 7.2 Hz, 1H), 1.79–1.88 (m, 1H), 1.59–1.68 (m, 3H), 1.50–1.53 (m, 2H), 1.10–1.20 (m, 3H), 0.90–0.99 (m, 2H). ^13^C-NMR (DMSO-*d*_6_, 100 MHz) δ: 166.15, 142.36, 49.58, 36.14, 29.69, 25.75, 24.97.

*4-(1-Adamantylmethyl)-2,3-dihydro-4H-1,2,4-triazoline-3-thione* (**5d**): White solid; 18.40 g (82%); m.p. 223–225 °C. ^1^H-NMR (DMSO-*d*_6_, 400 MHz) δ: 13.65 (brs, 1H), 8.34 (s, 1H), 3.68 (s, 2H), 1.93 (s, 3H), 1.62–1.65 (m, 3H), 1.47–1.56 (m, 9H). ^13^C-NMR (DMSO-*d*_6_, 100 MHz) δ: 167.29, 142.86, 54.42, 39.85, 36.14, 34.59, 27.48.

*4-Benzyl-2,3-dihydro-4H-1,2,4-triazoline-3-thione* (**5e**): White solid; 13.08 g (76%); m.p. 121–123 °C. ^1^H-NMR (DMSO-*d*_6_, 400 MHz) δ: 13.76 (brs, 1H), 8.54 (s, 1H), 7.29–7.38 (m, 5H), 5.16 (s, 2H). ^13^C-NMR (DMSO-*d*_6_, 100 MHz) δ: 166.28, 142.04, 135.99, 128.60, 127.85, 127.75, 46.88.

*4-(3-Pyridinylmethyl)-2,3-dihydro-4H-1,2,4-triazoline-3-thione* (**5f**): White solid; 12.46 g (72%); m.p. 211–213 °C. ^1^H-NMR (DMSO-*d*_6_, 400 MHz) δ: 13.80 (brs, 1H), 8.62 (d, *J =* 2.0 H, 1Hz), 8.60 (s, 1H), 8.51–8.52 (m, 1H), 7.75–7.78 (m, 1H), 7.37–7.40 (m, 1H), 5.20 (s, 2H). ^13^C-NMR (DMSO-*d*_6_, 100 MHz) δ: 166.22, 149.19, 149.10, 142.02, 135.66, 131.61, 123.62, 44.70.

*4-(2-Furanylmethyl)-2,3-dihydro-4H-1,2,4-triazoline-3-thione* (**5g**): White solid; 12.72 g (78%); m.p. 133–135 °C. ^1^H-NMR (DMSO-*d*_6_, 400 MHz) δ: 13.76 (brs, 1H), 8.45 (s, 1H), 7.64 (d, *J =* 1.6 Hz, 1H), 6.43–6.46 (m, 2H), 5.17 (s, 2H). ^13^C-NMR (DMSO-*d*_6_, 100 MHz) δ: 166.04, 148.30, 143.35, 141.74, 110.75, 109.46, 40.41.

*4-(2-Thiophenylmethyl)-2,3-dihydro-4H-1,2,4-triazoline-3-thione* (**5h**): White solid; 13.50 g (76%); m.p. 108–110 °C. ^1^H-NMR (DMSO-*d*_6_, 400 MHz) δ: 13.74 (brs, 1H), 8.54 (s, 1H), 7.47 (dd, *J =* 1.2 Hz and 4.8 Hz, 1H), 7.20 (d, *J =* 3.2 Hz, 1H), 6.99–7.01 (m, 1H), 5.33 (s, 2H). ^13^C-NMR (DMSO-*d*_6_, 100 MHz) δ: 165.84, 141.62, 137.60, 127.85, 126.91, 126.81, 41.73.

*4-(Biphenyl-4-methyl)-2,3-dihydro-4H-1,2,4-triazoline-3-thione* (**5i**): White solid; 18.53 g (77%); m.p. 178–180 °C. ^1^H-NMR (DMSO-*d*_6_, 400 MHz) δ: 13.79 (brs, 1H), 8.59 (d, *J =* 1.2 Hz, 1H), 7.63–7.66 (m, 4H), 7.43–7.47 (m, 4H), 7.36 (t, *J =* 7.2 Hz, 1H), 5.21 (s, 2H). ^13^C-NMR (DMSO-*d*_6_, 100 MHz) δ: 166.29, 142.09, 139.77, 139.59, 135.16, 128.89, 128.40, 127.51, 126.91, 126.63, 46.61.

*4-(Diphenylmethyl)-2,3-dihydro-4H-1,2,4-triazoline-3-thione* (**5j**): White solid; 18.04 g (75%); m.p. 166–168 °C. ^1^H-NMR (DMSO-*d*_6_, 400 MHz) δ: 13.94 (brs, 1H), 8.50 (s, 1H), 7.35–7.43 (m, 6H), 7.18–7.19 (m, 4H), 7.08 (s, 1H). ^13^C-NMR (DMSO-*d*_6_, 100 MHz) δ: 166.38, 140.77, 138.00, 128.84, 128.15, 127.99, 61.08.

*4-(Benzo[b]furan-5-methyl)-2,3-dihydro-4H-1,2,4-triazoline-3-thione* (**5k**): White solid; 14.78 g (71%); m.p. 151–153 °C. ^1^H-NMR (DMSO-*d*_6_, 400 MHz) δ: 13.75 (brs, 1H), 8.54 (s, 1H), 7.99 (d, *J =* 2.0 Hz, 1H), 7.66 (d, *J =* 1.2 Hz, 1H), 7.58 (d, *J =* 8.4 Hz, 1H), 7.35–7.38 (m, 1H), 6.96 (d, *J =* 2.8 Hz, 1H), 5.24 (s, 2H). ^13^C-NMR (DMSO-*d*_6_, 100 MHz) δ: 166.17, 153.86, 146.64, 142.00, 130.77, 127.39, 124.46, 120.93, 111.41, 106.71, 47.00.

*4-((4-Cyano-1-naphthyl)methyl]-2,3-dihydro-4H-1,2,4-triazoline-3-thione* (**5l**): White solid; 15.64 g (72%); m.p. 218–220 °C. ^1^H-NMR (DMSO-*d*_6_, 400 MHz) δ: 13.94 (brs, 1H), 8.46 (s, 1H), 8.36 (d, *J =* 8.4 Hz, 1H), 8.19 (d, *J =* 8.0 Hz, 1H), 8.16 (d, *J =* 7.2 Hz, 1H), 7.79–7.88 (m, 2H), 7.25 (d, *J =* 7.6 Hz, 1H), 5.74 (s, 2H). ^13^C-NMR (DMSO-*d*_6_, 100 MHz) δ: 166.49, 142.16, 137.78, 132.88, 131.56, 129.83, 129.12, 128.35, 125.09, 124.64, 124.30, 117.36, 109.18, 45.06.

*4-(2-Naphthylmethyl)-2,3-dihydro-4H-1,2,4-triazoline-3-thione* (**5m**): White solid; 14.77 g (68%); m.p. 165–167 °C. ^1^H-NMR (DMSO-*d*_6_, 400 MHz) δ: 13.80 (brs, 1H), 8.59 (d, *J =* 1.2 Hz, 1H), 7.88–7.92 (m, 3H), 7.81 (s, 1H), 7.49–7.54 (m, 3H), 5.33 (s, 2H). ^13^C-NMR (DMSO-*d*_6_, 100 MHz) δ: 166.35, 142.16, 133.51, 132.67, 132.39, 128.36, 127.72, 127.55, 126.53, 126.45, 126.25, 125.63, 47.12.

*4-(Quinoline-6-methyl)-2,3-dihydro-4H-1,2,4-triazoline-3-thione* (**5n**): White solid; 16.36 g (75%); m.p. 272–275 °C. ^1^H-NMR (DMSO-*d*_6_, 400 MHz) δ: 13.82 (brs, 1H), 8.89 (dd, *J =* 1.6 Hz and 4.4 Hz, 1H), 8.62 (s, 1H), 8.35 (d, *J =* 8.4 Hz, 1H), 8.01 (d, *J =* 8.8 Hz, 1H), 7.85 (s, 1H), 7.75 (dd, *J =* 2.0 Hz and 8.8 Hz, 1H), 7.51–7.55 (m, 1H), 5.37 (s, 2H). ^13^C-NMR (DMSO-*d*_6_, 100 MHz) δ: 166.36, 150.76, 147.16, 142.19, 135.97, 134.17, 129.41, 129.15, 127.55, 126.69, 121.81, 46.85.

*4-((5-Chloro-2-thiophenyl)methyl]-2,3-dihydro-4H-1,2,4-triazoline-3-thione* (**5ha**): White solid; 15.02 g (72%); m.p. 176–178 °C. ^1^H-NMR (DMSO-*d*_6_, 400 MHz) δ: 13.79 (brs, 1H), 8.55 (s, 1H), 7.08 (d, *J =* 3.6 Hz, 1H), 7.01 (d, *J =* 3.6 Hz, 1H), 5.25 (s, 2H). ^13^C-NMR (DMSO-*d*_6_, 100 MHz) δ: 165.77, 141.54, 136.87, 128.71, 128.02, 126.34, 42.01.

*4-((5-Methyl-2-thiophenyl)methyl]-2,3-dihydro-4H-1,2,4-triazoline-3-thione* (**5hb**): White solid; 13.31 g (70%); m.p. 131–133 °C. ^1^H-NMR (DMSO-*d*_6_, 400 MHz) δ: 13.72 (brs, 1H), 8.50 (s, 1H), 6.98 (d, *J =* 3.2 Hz, 1H), 6.66–6.67 (m, 1H), 5.23 (s, 2H), 2.37 (s, 3H). ^13^C-NMR (DMSO-*d*_6_, 100 MHz) δ: 165.81, 141.56, 140.31, 135.16, 127.87, 125.00, 42.01, 14.87.

*4-((5-Ethyl-2-thiophenyl)methyl]-2,3-dihydro-4H-1,2,4-triazoline-3-thione* (**5hc**): White solid; 15.21 g (75%); m.p. 64–66 °C. ^1^H-NMR (DMSO-*d*_6_, 400 MHz) δ: 13.73 (brs, 1H), 8.51 (s, 1H), 7.00 (d, *J =* 3.6 Hz, 1H), 6.70 (d, *J =* 3.2 Hz, 1H), 5.25 (s, 2H), 2.73 (q, *J =* 7.5 Hz, 2H), 1.18 (t, *J =* 7.6 Hz, 3H). ^13^C-NMR (DMSO-*d*_6_, 100 MHz) δ: 165.84, 147.80, 141.56, 134.82, 127.65, 123.22, 42.06, 22.75, 15.72.

*4-((3-Methyl-2-thiophenyl)methyl]-2,3-dihydro-4H-1,2,4-triazoline-3-thione* (**5hd**): White solid; 14.83 g (78%); m.p. 113–115 °C. ^1^H-NMR (DMSO-*d*_6_, 400 MHz) δ: 13.74 (brs, 1H), 8.41 (s, 1H), 7.38 (d, *J =* 5.2 Hz, 1H), 6.86 (d, *J =* 5.2 Hz, 1H), 5.26 (s, 2H), 2.28 (s, 3H). ^13^C-NMR (DMSO-*d*_6_, 100 MHz) δ: 165.87, 141.59, 136.18, 130.91, 129.96, 125.12, 40.26, 13.64.

*4-((4-Methyl-2-thiophenyl)methyl]-2,3-dihydro-4H-1,2,4-triazoline-3-thione* (**5he**): White solid; 14.45 g (76%); m.p. 98–100 °C. ^1^H-NMR (DMSO-*d*_6_, 400 MHz) δ: 13.73 (brs, 1H), 8.52 (s, 1H), 7.03 (s, 1H), 6.99 (s, 1H), 5.26 (s, 2H), 2.15 (s, 3H). ^13^C-NMR (DMSO-*d*_6_, 100 MHz) δ: 165.86, 141.61, 137.39, 136.83, 129.84, 121.71, 41.90, 15.26.

*4-((3-Thiophenyl)methyl]-2,3-dihydro-4H-1,2,4-triazoline-3-thione* (**5hf**): White solid; 12.55 g (70%); m.p. 109–110 °C. ^1^H-NMR (DMSO-*d*_6_, 400 MHz) δ: 13.73 (brs, 1H), 8.51 (s, 1H), 7.53 (dd, *J =* 3.2 Hz and 4.8 Hz, 1H), 7.47 (d, *J =* 1.6 Hz, 1H), 7.16 (dd, *J =* 0.8 Hz and 4.8 Hz, 1H), 5.13 (s, 2H). ^13^C-NMR (DMSO-*d*_6_, 100 MHz) δ: 166.01, 141.86, 136.42, 127.40, 127.06, 124.18, 42.41.

*4-((5-Phenyl-2-thiophenyl)methyl]-2,3-dihydro-4H-1,2,4-triazoline-3-thione* (**5hg**): White solid; 16.73 g (68%); m.p. 160–162 °C. ^1^H-NMR (DMSO-*d*_6_, 400 MHz) δ: 13.78 (brs, 1H), 8.58 (s, 1H), 7.58–7.60 (m, 2H), 7.37–7.41 (m, 3H), 7.30 (t, *J =* 7.4 Hz, 1H), 7.20 (d, *J =* 3.6 Hz, 1H), 5.34 (s, 2H). ^13^C-NMR (DMSO-*d*_6_, 100 MHz) δ: 165.88, 143.95, 141.64, 137.14, 133.38, 129.18, 129.09, 127.78, 125.27, 123.27, 42.01.

*4-((Benzo[b]thiophen-2-yl)methyl]-2,3-dihydro-4H-1,2,4-triazoline-3-thione* (**5hh**): White solid; 15.80 g (71%); m.p. 185–188 °C. ^1^H-NMR (DMSO-*d*_6_, 400 MHz) δ: 13.82 (brs, 1H), 8.60 (s, 1H), 7.90–7.92 (m, 1H), 7.81–7.83 (m, 1H), 7.45 (s, 1H), 7.31–7.38 (m, 2H), 5.45 (s, 2H). ^13^C-NMR (DMSO-*d*_6_, 100 MHz) δ: 166.19, 141.85, 139.50, 138.90, 138.87, 124.79, 124.66, 124.18, 123.80, 122.56, 42.84.

*4-((Dibenzo[b,d]thiophen-4-yl)methyl]-2,3-dihydro-4H-1,2,4-triazoline-3-thione* (**5hi**): White solid; 18.20 g (68%); m.p. 240–242 °C. ^1^H-NMR (DMSO-*d*_6_, 400 MHz) δ: 13.87 (brs, 1H), 8.46 (s, 1H), 8.35–8.40 (m, 2H), 8.05–8.08 (m, 1H), 7.51–7.56 (m, 3H), 7.29 (d, *J =* 7.2 Hz, 1H), 5.42 (s, 2H). ^13^C-NMR (DMSO-*d*_6_, 100 MHz) δ: 166.71, 142.14, 138.15, 136.79, 135.78, 134.94, 129.79, 127.32, 126.14, 125.13, 124.95, 123.00, 122.20, 121.67, 46.32. 

#### 3.2.5. General Procedure for the Synthesis of Thiones **5o** and **5q**

A solution of isothiocyanate **3l** or **3h** (90 mmol) and NH_2_CH_2_CH(OMe)_2_ (11.36 g, 108 mmol) in THF (150 mL) was stirred at room temperature until the reaction completed as indicated by TLC analysis. The reaction mixture was evaporated on a rotary evaporator to give the crude intermediate **4o** or **4p**, which was used directly in the next step without further purification. Thus, the crude **4o** or **4p** was suspended in 25% aqueous H_2_SO_4_ containing 20% acetic acid (by *v*/*v*) and stirred at reflux for 3 h when TLC analysis indicated the reaction was completed. On cooling to room temperature, the reaction mixture was poured into ice-water (450 mL) and the white crystals were collected by vacuum filtration, washed with water and dried in vacuo to give the crude product **5o** or **5q**, which was triturated with EtOAc/*n*-hexane to yield the pure product **5o** or **5q**.

*1-((4-Cyano-1-naphthyl)methyl)-2,3-dihydro-1H-imidazoline-2-thione* (**5o**): White solid; 17.43 g (73%); m.p. 287–289 °C. ^1^H-NMR (DMSO-*d*_6_, 400 MHz) δ: 12.33 (brs, 1H), 8.40 (d, *J =* 8.4 Hz, 1H), 8.14–8.18 (m, 2H), 7.85 (t, *J =* 7.4 Hz, 1H), 7.78 (t, *J =* 7.6 Hz, 1H), 7.18 (d, *J =* 7.2 Hz, 1H), 6.97–7.01 (m, 2H), 5.73 (s, 2H). ^13^C-NMR (DMSO-*d*_6_, 100 MHz) δ: 161.66, 139.23, 132.89, 131.55, 130.03, 129.04, 128.17, 124.97, 124.62, 124.49, 118.38, 117.47, 115.13, 108.77, 46.95.

*1-((2-Thiophenyl)methyl)-2,3-dihydro-1H-imidazoline-2-thione* (**5q**): White solid; 12.37 g (70%); m.p. 124–126 °C. ^1^H-NMR (DMSO-*d*_6_, 400 MHz) δ: 12.12 (brs, 1H), 7.43 (d, *J =* 5.2 Hz, 1H), 7.16 (d, *J =* 3.2 Hz, 1H), 7.08–7.09 (m, 1H), 6.98 (dd, *J =* 3.6 Hz and 5.2 Hz, 1H), 6.86–6.87 (m, 1H), 5.31 (s, 2H). ^13^C-NMR (DMSO-*d*_6_, 100 MHz) δ: 160.94, 138.85, 127.27, 126.68, 126.32, 117.81, 114.64, 43.57.

#### 3.2.6. Procedure for the Synthesis of (4-Cyano-1-naphthyl)(4-mercapto-3-pyridinyl)methane (**5p**)

A solution of **17** (49.22 g, 200 mmol) and hexamethylenetetramine (56.07 g, 400 mmol) in 50% acetic acid was stirred at reflux for 6 h followed by addition of concentrated hydrochloric acid (10 mL). The reflux was continued for another 0.5 h, when the reaction completed as indicated by TLC analysis. On cooling to room temperature, the reaction mixture was concentrated to half its original volume and poured into ice-water (300 mL) while stirring. The resulting mixture was extracted with CH_2_Cl_2_ (200 mL × 3), and the combined extracts were washed successively with saturated aqueous NaHCO_3_ (200 mL × 3) and 5% brine (300 mL), dried (Na_2_SO_4_) and evaporated on a rotary evaporator to give a residue, which was purified by column chromatography to afford 4-cyano-1-naphthaldehyde (**19**). Yellow solid; 30.45 g (78%); m.p. 138–141 °C (literature value, 143–144 °C [59]). ^1^H-NMR (DMSO-*d*_6_, 400 MHz) 10.54 (s, 1H), 9.17–9.21 (m, 1H), 8.41 (d, *J =* 7.2 Hz, 1H), 8.29 (d, *J =* 7.2 Hz, 1H), 8.23–8.27 (m, 1H), 7.89–7.93 (m, 2H). 

To a stirred solution of 3-bromo-4-chloropyridine (**20**, 28.86 g, 150 mmol ) in dried THF (350 mL) held at –78 °C in N_2_ atmosphere was added dropwise 1.6M *n*-BuLi in *n*-hexane (93.75 mL, 150 mmol) via syringe. After addition, the resulting mixture was stirred at this temperature for another 0.5 h, followed by addition of a solution of **19** (29.28 g, 150 mmol) in dried THF (150 mL) in a dropwise manner via syringe. The reaction mixture was slowly warmed to room temperature and stirred at room temperature for another 1 h. The reaction mixture was slowly poured into ice-water (600 mL). The mixture thus obtained was extracted with CH_2_Cl_2_ (200 mL × 3), and the combined extracts were washed with 5% brine (200 mL), dried (Na_2_SO_4_) and evaporated on a rotary evaporator to give a residue, which was purified by column chromatography to afford (4-chloro-3-pyridinyl)(4-cyano-1-naphthyl)methanol (**22**). White solid; 29.62 g (67%); m.p. 178–180 °C. ^1^H-NMR (DMSO-*d*_6_, 400 MHz) δ: 8.55 (s, 1H), 8.49 (d, *J =* 5.6 Hz, 1H), 8.21 (d, *J =* 8.4 Hz, 1H), 8.17 (d, *J =* 8.0 Hz, 1H), 8.16 (d, *J =* 7.6 Hz, 1H), 7.79–7.83 (m, 1H), 7.72–7.76 (m, 1H), 7.58 (d, *J =* 5.2 Hz, 1H), 7.57 (d, *J =* 7.6 Hz, 1H), 6.78 (d, *J =* 5.6 Hz, 1H), 6.62 (d, *J =* 6.0 Hz, 1H).

To a stirred solution of **22** (26.53 g, 90 mmol) in dried CH_2_Cl_2_ (300 mL) cooled in an ice-water bath was added thionyl chloride (12.85 g, 108 mmol). After addition, the resulting mixture was stirred at room temperature until the reaction completed as indicated by TLC analysis (typically within 4 h) and poured into ice-water (600 mL) while stirring. The mixture thus obtained was adjusted to pH 7–8 with saturated aqueous NaHCO_3_. The organic phase was separated, and the aqueous phase was back-extracted with CH_2_Cl_2_ (200 mL × 2). The combined organic phases were washed with 5% brine (300 mL), dried (Na_2_SO_4_) and evaporated on a rotary evaporator to give crude (4-chloro-3-pyridinyl)(4-cyano-1-naphthyl)methyl chloride (**23**) as a residue, which was used directly in the next step without further purification.

To a stirred solution of crude **23** prepared above (deemed to be 90 mmol) in acetic acid (300 mL) was added Zn powder (13.08 g, 200 mmol) in a portionwise manner at room temperature, and after addition, the reaction mixture was stirred at room temperature until the reaction completed as indicated by TLC analysis (typically within 5 h). The reaction mixture was diluted with CH_2_Cl_2_ (300 mL) and the resulting mixture was further stirred for 10 min and filtered off through Celite. The filtrate was evaporated on a rotary evaporator to almost dryness, and the residue was dissolved in CH_2_Cl_2_ (200 mL). The solution thus obtained was washed successively saturated aqueous NaHCO_3_ (200 mL) and 5% brine (200 mL), dried (Na_2_SO_4_) and evaporated on a rotary evaporator to give a residue, which was purified by column chromatography to afford (4-chloro-3-pyridinyl)(4-cyano-1-naphthyl)methane (**24**). White solid; 18.48 g (78%); m.p. 155–157 °C. ^1^H-NMR (DMSO-*d*_6_, 400 MHz) δ: 8.48 (d, *J =* 5.6 Hz, 1H), 8.39 (s, 1H), 8.31 (d, *J =* 8.4 Hz, 1H), 8.18 (d, *J =* 8.0 Hz, 1H), 8.08 (d, *J =* 7.6 Hz, 1H), 7.84 (t, *J =* 7.4 Hz, 1H), 7.76–7.80 (m, 1H), 7.63 (d, *J =* 5.2 Hz, 1H), 7.16 (d, *J =* 7.6 Hz, 1H), 4.68 (s, 2H).

A solution of **24** (15.33 g, 55 mmol) and Na_2_S·9H_2_O (39.63 g, 165 mmol) in DMF (200 mL) was stirred at 110 °C in N_2_ atmosphere until the reaction completed as indicated by TLC analysis (typically within 12 h). On cooling to room temperature, the reaction mixture was concentrated on a rotary evaporator to about 50 mL and poured into ice-water (600 mL) while stirring, and the mixture thus obtained was adjusted to pH 5–6 and extracted with CH_2_Cl_2_ (200 mL × 3). The combined organic phases were washed with 5% brine (100 mL), dried (Na_2_SO_4_) and evaporated on a rotary evaporator to give a residue, which was purified by column chromatography to afford the pure compound **5p**. White solid; 10.94 g (72%); m.p. 208–211 °C. ^1^H-NMR (DMSO-*d*_6_, 400 MHz) δ: 12.38 (brs, 1H), 8.08–8.15 (m, 3H), 7.79 (t, *J =* 7.8 Hz, 1H), 7.69 (t, *J =* 7.4 Hz, 1H), 7.56 (d, *J =* 6.4 Hz, 1H), 7.44 (d, *J =* 7.6 Hz, 1H), 7.39 (d, *J =* 6.8 Hz, 1H), 7.29 (s, 1H), 4.60 (s, 2H). ^13^C-NMR (DMSO-*d*_6_, 100 MHz) δ: 188.80, 143.10, 137.95, 132.97, 132.87, 131.75, 131.28, 131.19, 130.04, 128.70, 127.81, 126.05, 125.16, 124.92, 117.80, 107.49, 34.86.

#### 3.2.7. General Procedure for the Synthesis of Esters **6a**–**6x** and **6ha**–**6hi**

A mixture of thiones or thiol **5a**–**5q** or **5ha**–**5hi** (60 mmol), bromoacetates BrCH_2_CO_2_Me, (CH_3_)_2_CBrCO_2_Et or ethyl 1-bromocyclobutanecarboxylate (80 mmol) and K_2_CO_3_ (16.59 g, 120 mmol) in a suitable solvent (200 mL; acetone for **5a**–**5b** or DMF for the others) was stirred at a temperature specified in Scheme 1, Scheme 2, Scheme 3, Scheme 4 and Scheme 5 until the completion of reaction as indicated by TLC analysis (typically within 12 h). On cooling to room temperature (if necessary), for **5a**–**5b**: the reaction mixture was filtered off and the filtrate was evaporated on a rotary evaporator to give a residue, which was purified by column chromatography to afford **6a**–**6b**; for the others: the reaction mixture was poured into ice-water (300 mL), and the resulting mixture was extracted with CH_2_Cl_2_ (100 mL × 3). The combined extracts were washed with 5% brine (100 mL × 5), dried (Na_2_SO_4_) and evaporated on a rotary evaporator to afford the crude product as a residue, which was purified by column chromatography to yield the pure product **6c**–**6x** or **6ha**–**6hi**.

*Methyl 2-((4-ethyl-4H-1,2,4-triazol-3-yl)thio)acetate* (**6a**): White solid; 9.32 g (83%); m.p. 67–69 °C. ^1^H-NMR (DMSO-*d*_6_, 400 MHz) δ: 8.53 (s, 1H), 4.03 (s, 2H), 3.63 (s, 3H), 3.58 (s, 3H). ^13^C-NMR (DMSO-*d*_6_, 100 MHz) δ: 168.85, 148.31, 146.26, 52.42, 34.56, 30.73.

*Methyl 2-((4-n-propyl-4H-1,2,4-triazol-3-yl)thio)acetate* (**6b**): Colorless oil; 9.42 g (78%). ^1^H-NMR (DMSO-*d*_6_, 400 MHz) δ: 8.60 (s, 1H), 4.06 (s, 2H), 3.96 (q, *J =* 7.3 Hz, 2H), 3.63 (s, 3H), 1.31 (t, *J =* 7.2 Hz, 3H). ^13^C-NMR (DMSO-*d*_6_, 100 MHz) δ: 168.78, 147.56, 145.03, 52.41, 39.37, 34.50, 15.19.

*Methyl 2-((4-(cyclohexylmethyl)-4H-1,2,4-triazol-3-yl)thio)acetate* (**6c**): White solid; 13.25 g (82%); m.p. 56–58 °C. ^1^H-NMR (DMSO-*d*_6_, 400 MHz) δ: 8.55 (s, 1H), 4.07 (s, 2H), 3.79 (d, *J =* 7.2 Hz, 2H), 3.63 (s, 3H), 1.59–1.72 (m, 4H), 1.47–1.50 (m, 2H), 1.09–1.21 (m, 3H), 0.87–0.96 (m, 2H). ^13^C-NMR (DMSO-*d*_6_, 100 MHz) δ: 168.67, 148.12, 145.87, 52.35, 49.80, 37.67, 34.50, 29.63, 25.64, 24.94.

*Methyl 2-((4-(1-adamantylmethyl)-4H-1,2,4-triazol-3-yl)thio)acetate* (**6d**): White solid; 15.62 g (81%); m.p. 214–216 °C. ^1^H-NMR (DMSO-*d*_6_, 400 MHz) δ: 8.48 (s, 1H), 4.07 (s, 2H), 3.65 (s, 2H), 3.62 (s, 3H), 1.94 (s, 3H), 1.63–1.66 (m, 3H), 1.52–1.55 (m, 3H), 1.42 (s, 6H). ^13^C-NMR (CDCl_3_, 100 MHz) δ: 168.96, 150.42, 145.75, 56.82, 52.99, 40.22, 36.49, 35.79, 34.72, 28.06.

*Methyl 2-((4-benzyl-4H-1,2,4-triazol-3-yl)thio)acetate* (**6e**): White solid; 12.01 g (76%); m.p. 82–84 °C. ^1^H-NMR (DMSO-*d*_6_, 400 MHz) δ: 8.71 (s, 1H), 7.32–7.40 (m, 3H), 7.22–7.23 (m, 2H), 5.21 (s, 2H), 4.03 (s, 2H), 3.62 (s, 3H). ^13^C-NMR (DMSO-*d*_6_, 100 MHz) δ: 168.63, 148.22, 145.77, 135.59, 128.79, 128.06, 127.41, 52.39, 47.34, 34.39.

*Methyl 2-((4-(3-pyridinylmethyl)-4H-1,2,4-triazol-3-yl)thio)acetate* (**6f**): Colorless oil; 10.78 g (68%). ^1^H-NMR (DMSO-*d*_6_, 400 MHz) δ: 8.74 (s, 1H), 8.53–8.54 (m, 2H), 7.63–7.65 (m, 1H), 7.39–7.42 (m, 1H), 5.27 (s, 2H), 4.05 (s, 2H), 3.61 (s, 3H). ^13^C-NMR (DMSO-*d*_6_, 100 MHz) δ: 168.60, 149.36, 148.89, 148.17, 145.75, 135.39, 131.28, 123.80, 52.41, 45.01, 34.40.

*Methyl 2-((4-(2-furanylmethyl)-4H-1,2,4-triazol-3-yl)thio)acetate* (**6g**): White solid; 11.40 g (75%); m.p. 67–69 °C. ^1^H-NMR (DMSO-*d*_6_, 400 MHz) δ: 8.63 (s, 1H), 7.65 (d, *J =* 1.6 Hz, 1H), 6.48 (d, *J =* 3.2 Hz, 1H), 6.44–6.46 (m, 1H), 5.25 (s, 2H), 4.04 (s, 2H), 3.63 (s, 3H). ^13^C-NMR (DMSO-*d*_6_, 100 MHz) δ: 168.71, 148.16, 148.14, 145.54, 143.72, 110.78, 109.57, 52.41, 40.65, 34.56.

*Methyl 2-((4-(2-thiophenylmethyl)-4H-1,2,4-triazol-3-yl)thio)acetate* (**6h**): Colorless oil; 16.30 g (78%). ^1^H-NMR (DMSO-*d*_6_, 400 MHz) δ: 8.70 (s, 1H), 7.52–7.53 (m, 1H), 7.12–7.13 (m, 1H), 7.01–7.03 (m, 1H), 5.42 (s, 2H), 4.05 (s, 2H), 3.62 (s, 3H). ^13^C-NMR (DMSO-*d*_6_, 100 MHz) δ: 168.66, 147.95, 145.35, 137.68, 127.64, 127.21, 127.02, 52.40, 42.25, 34.61.

*Methyl 2-((4-(biphenyl-4-methyl)-4H-1,2,4-triazol-3-yl)thio)acetate* (**6i**): White solid; 15.48 g (76%); m.p. 152–154 °C. ^1^H-NMR (DMSO-*d*_6_, 400 MHz) δ: 8.76 (s, 1H), 7.64–7.68 (m, 4H), 7.45 (t, *J =* 7.6 Hz, 2H), 7.37 (d, *J =* 7.2 Hz, 1H), 7.31–7.34 (m, 2H), 5.26 (s, 2H), 4.05 (s, 2H), 3.62 (s, 3H). ^13^C-NMR (DMSO-*d*_6_, 100 MHz) δ: 168.65, 148.24, 145.81, 139.92, 139.43, 134.74, 128.91, 128.05, 127.59, 127.07, 126.64, 52.40, 47.04, 34.40.

*Methyl 2-((4-(diphenylmethyl)-4H-1,2,4-triazol-3-yl)thio)acetate* (**6j**): Colorless oil; 16.90 g (83%). ^1^H-NMR (DMSO-*d*_6_, 400 MHz) δ: 8.30 (s, 1H), 7.36–7.44 (m, 6H), 7.18–7.19 (m, 4H), 6.79 (s, 1H), 4.03 (s, 2H), 3.61 (s, 3H). ^13^C-NMR (DMSO-*d*_6_, 100 MHz) δ: 168.64, 148.70, 144.48, 137.65, 128.92, 128.47, 127.99, 62.12, 52.41, 34.58.

*Methyl 2-((4-(benzo[b]furan-5-methyl)-4H-1,2,4-triazol-3-yl)thio)acetate* (**6k**): White solid; 14.38 g (79%); m.p. 82–85 °C. ^1^H-NMR (DMSO-*d*_6_, 400 MHz) δ: 8.74 (s, 1H), 8.01 (d, *J =* 2.4 Hz, 1H), 7.60 (d, *J =* 8.8 Hz, 1H), 7.54 (s, 1H), 7.22–7.24 (m, 1H), 6.96 (d, *J =* 1.2 Hz, 1H), 5.29 (s, 2H), 4.04 (s, 2H), 3.61 (s, 3H). ^13^C-NMR (DMSO-*d*_6_, 100 MHz) δ: 168.66, 153.88, 148.17, 146.83, 145.73, 130.27, 127.53, 124.05, 120.55, 111.59, 106.71, 52.38, 47.46, 34.37.

*Methyl 2-((4-((4-cyano-1-naphthyl)methyl)-4H-1,2,4-triazol-3-yl)thio)acetate* (**6l**): White solid; 16.65 g (82%); m.p. 135–137 °C. ^1^H-NMR (DMSO-*d*_6_, 400 MHz) δ: 8.72 (s, 1H), 8.30 (d, *J =* 8.0 Hz, 1H), 8.20 (d, *J =* 8.0 Hz, 1H), 8.15 (d, *J =* 7.6 Hz, 1H), 7.81–7.90 (m, 2H), 7.00 (d, *J =* 7.2 Hz, 1H), 5.87 (s, 2H), 4.07 (s, 2H), 3.61 (s, 3H). ^13^C-NMR (DMSO-*d*_6_, 100 MHz) δ: 168.59, 148.78, 146.17, 137.70, 132.92, 131.49, 129.58, 129.27, 128.47, 125.08, 124.17, 123.82, 117.29, 109.30, 52.45, 45.32, 34.53.

*Methyl 2-((4-(2-naphthylmethyl)-4H-1,2,4-triazol-3-yl)thio)acetate* (**6m**): White solid; 15.04 g (80%); m.p. 101–103 °C. ^1^H-NMR (DMSO-*d*_6_, 400 MHz) δ: 8.78 (s, 1H), 7.88–7.94 (m, 3H), 7.73 (s, 1H), 7.51–7.55 (m, 2H), 7.37–7.39 (m, 1H), 5.39 (s, 2H), 4.03 (s, 2H), 3.60 (s, 3H). ^13^C-NMR (DMSO-*d*_6_, 100 MHz) δ: 168.63, 148.34, 145.90, 133.06, 132.67, 132.42, 128.58, 127.74, 127.58, 126.57, 126.40, 126.27, 125.16, 52.36, 47.55, 34.38.

*Methyl 2-((4-(quinoline-6-methyl)-4H-1,2,4-triazol-3-yl)thio)acetate* (**6n**): White solid; 14.33 g (76%); m.p. 141–143 °C. ^1^H-NMR (DMSO-*d*_6_, 400 MHz) δ: 8.90 (dd, *J =* 1.6 Hz and 4.4 Hz, 1H), 8.80 (s, 1H), 8.35 (d, *J =* 8.0 Hz, 1H), 8.03 (d, *J =* 8.4 Hz, 1H), 7.77 (d, *J =* 0.8 Hz, 1H), 7.62–7.65 (m, 1H), 7.54 (dd, *J =* 4.4 Hz and 8.4 Hz, 1H), 5.44 (s, 2H), 4.04 (s, 2H), 3.59 (s, 3H). ^13^C-NMR (DMSO-*d*_6_, 100 MHz) δ: 168.63, 150.93, 148.38, 147.20, 145.95, 136.00, 133.77, 129.65, 128.72, 127.61, 126.46, 121.94, 52.38, 47.26, 34.43.

*Ethyl 2-((4-((4-cyano-1-naphthyl)methyl)-4H-1,2,4-triazol-3-yl)thio)-2-methylpropionate* (**6o**): White solid; 14.38 g (63%); m.p. 110–112 °C. ^1^H-NMR (DMSO-*d*_6_, 400 MHz) δ: 8.81 (s, 1H), 8.29 (d, *J =* 8.4 Hz, 1H), 8.20 (d, *J =* 8.4 Hz, 1H), 8.12 (d, *J =* 7.6 Hz, 1H), 7.82–7.91 (m, 2H), 6.85 (d, *J =* 7.6 Hz, 1H), 5.86 (s, 2H), 4.06 (q, *J =* 7.1 Hz, 2H), 1.52 (s, 6H), 1.10 (t, *J =* 7.2 Hz, 3H). ^13^C-NMR (DMSO-*d*_6_, 100 MHz) δ: 172.09, 146.45, 138.21, 132.96, 131.44, 129.39, 129.28, 129.24, 128.46, 125.11, 123.97, 123.36, 117.22, 109.28, 61.36, 53.28, 45.41, 25.84, 13.67.

*Ethyl 1-((4-((4-cyano-1-naphthyl)methyl)-4H-1,2,4-triazol-3-yl)thio)cyclobutanecarboxylate* (**6p**): White solid; 15.01 g (58%); m.p. 141–143 °C. ^1^H-NMR (DMSO-*d*_6_, 400 MHz) δ: 8.79 (s, 1H), 8.29 (d, *J =* 8.4 Hz, 1H), 8.20 (d, *J =* 8.0 Hz, 1H), 8.14 (d, *J =* 7.2 Hz, 1H), 7.82–7.91 (m, 2H), 6.89 (d, *J =* 7.6 Hz, 1H), 5.85 (s, 2H), 4.05 (q, *J =* 7.1 Hz, 2H), 2.57–2.65 (m, 2H), 2.24–2.31 (m, 2H), 2.03–2.12 (m, 1H), 1.83–1.91 (m, 1H), 1.09 (t, *J =* 7.0 Hz, 3H). ^13^C-NMR (DMSO-*d*_6_, 100 MHz) δ: 171.64, 146.97, 146.25, 138.08, 132.98, 131.47, 129.44, 129.33, 128.51, 125.15, 123.99, 123.47, 117.26, 109.30, 61.28, 54.07, 45.37, 31.76, 15.55, 13.72.

*Methyl 2-((1-((4-cyano-1-naphthyl)methyl)-1H-imidazol-2-yl)thio)acetate* (**6q**): White solid; 14.58 g (72%); m.p. 116–118 °C. ^1^H-NMR (DMSO-*d*_6_, 400 MHz) δ: 8.32 (d, *J =* 8.0 Hz, 1H), 8.19 (d, *J =* 8.0 Hz, 1H), 8.13 (d, *J =* 7.6 Hz, 1H), 7.81–7.89 (m, 2H), 7.34 (s, 1H), 7.07 (d, *J =* 1.2 Hz, 1H), 6.81 (d, *J =* 7.6 Hz, 1H), 5.83 (s, 2H), 3.91 (s, 2H), 3.58 (s, 3H). ^13^C-NMR (DMSO-*d*_6_, 100 MHz) δ: 169.09, 139.81, 139.44, 133.02, 131.45, 129.58, 129.51, 129.18, 128.32, 125.04, 124.17, 123.08, 123.06, 117.40, 108.86, 52.26, 47.09, 35.26.

*Ethyl 2-((1-((4-cyano-1-naphthyl)methyl)-1H-imidazol-2-yl)thio)-2-methylpropionate* (**6r**): White solid; 17.76 g (78%); m.p. 108–110 °C. ^1^H-NMR (DMSO-*d*_6_, 400 MHz) δ: 8.28 (d, *J =* 8.4 Hz, 1H), 8.18 (d, *J =* 8.0 Hz, 1H), 8.10 (d, *J =* 7.2 Hz, 1H), 7.81–7.89 (m, 2H), 7.46 (d, *J =* 1.2 Hz, 1H), 7.19 (d, *J =* 0.4 Hz, 1H), 6.67 (d, *J =* 7.6 Hz, 1H), 5.86 (s, 2H), 4.04 (q, *J =* 7.2 Hz, 2H), 1.46 (s, 6H), 1.11 (t, *J =* 7.0 Hz, 3H). ^13^C-NMR (DMSO-*d*_6_, 100 MHz) δ: 172.56, 139.92, 137.14, 133.05, 131.38, 130.41, 129.38, 129.21, 128.35, 125.06, 124.11, 123.97, 122.69, 117.36, 108.80, 61.08, 52.84, 47.33, 25.62, 13.72.

*Ethyl 1-((1-((4-cyano-1-naphthyl)methyl)-1H-imidazol-2-yl)thio)cyclobutanecarboxylate* (**6s**): White solid; 17.85 g (76%); m.p. 135–137 °C. ^1^H-NMR (DMSO-*d*_6_, 400 MHz) δ: 8.28 (d, *J =* 8.4 Hz, 1H), 8.19 (d, *J =* 7.6 Hz, 1H), 8.10–8.12 (d, *J* = 7.6 Hz, 1H), 7.81–7.90 (m, 2H), 7.45 (s, 1H), 7.16 (s, 1H), 6.71 (d, *J =* 7.6 Hz, 1H), 5.84 (s, 2H), 4.03 (q, *J =* 7.1 Hz, 2H), 2.53–2.56 (m, 2H), 2.23–2.29 (m, 2H), 1.98–2.07 (m, 1H), 1.76–1.84 (m, 1H), 1.09 (t, *J =* 7.2 Hz, 3H). ^13^C-NMR (DMSO-*d*_6_, 100 MHz) δ: 172.24, 139.80, 137.62, 133.05, 131.40, 130.32, 129.41, 129.22, 128.37, 125.07, 123.95, 123.88, 122.79, 117.37, 108.83, 61.02, 54.17, 47.28, 31.33, 15.32, 13.75.

*Methyl 2-((3-((4-cyano-1-naphthyl)methyl)pyridin-4-yl)thio)acetate* (**6t**): Colorless oil; 15.05 g (72%). ^1^H-NMR (DMSO-*d*_6_, 400 MHz) δ: 8.37 (d, *J =* 5.2 Hz, 1H), 8.28 (d, *J =* 8.4 Hz, 1H), 8.17 (d, *J =* 8.4 Hz, 1H), 8.12 (s, 1H), 8.07 (d, *J =* 7.2 Hz, 1H), 7.84 (t, *J =* 7.4 Hz, 1H), 7.76 (t, *J =* 7.6 Hz, 1H), 7.34 (d, *J =* 5.2 H, 1Hz), 7.15 (d, *J =* 7.6 Hz, 1H), 4.56 (s, 2H), 4.12 (s, 2H), 3.65 (s, 3H). ^13^C-NMR (DMSO-*d*_6_, 100 MHz) δ: 168.93, 149.63, 147.96, 146.71, 141.46, 132.96, 131.69, 131.07, 130.96, 128.92, 128.07, 125.40, 125.05, 124.84, 119.59, 117.64, 107.90, 52.50, 33.09, 32.16.

*Ethyl 2-((3-((4-cyano-1-naphthyl)methyl)pyridin-4-yl)thio)-2-methylpropionate* (**6u**): Colorless oil; 15.93 g (68%). ^1^H-NMR (DMSO-*d*_6_, 400 MHz) δ: 8.45 (d, *J =* 5.2 Hz, 1H), 8.30 (s, 1H), 8.27 (d, *J =* 8.4 Hz, 1H), 8.17 (d, *J =* 8.0 Hz, 1H), 8.06 (d, *J =* 7.6 Hz, 1H), 7.84 (t, *J =* 7.4 Hz, 1H), 7.78 (t, *J =* 7.2 Hz, 1H), 7.35 (d, *J =* 5.2 Hz, 1H), 7.05 (d, *J =* 3.6 Hz, 1H), 4.64 (s, 2H), 4.12 (q, *J =* 7.1 Hz, 2H), 1.51 (s, 6H), 1.13 (t, *J =* 7.2 Hz, 3H). ^13^C-NMR (DMSO-*d*_6_, 100 MHz) δ: 172.82, 150.91, 147.99, 143.04, 142.39, 135.60, 132.96, 131.66, 130.93, 128.94, 128.06, 126.75, 125.55, 125.05, 124.81, 117.61, 107.83, 61.39, 50.98, 33.44, 25.84, 13.75.

*Ethyl 1-((3-((4-cyano-1-naphthyl)methyl)pyridin-4-yl)thio)cyclobutanecarboxylate* (**6v**): White solid; 17.87 g (74%); m.p. 123–125 °C. ^1^H-NMR (DMSO-*d*_6_, 400 MHz) δ: 8.36 (d, *J =* 5.2 Hz, 1H), 8.27 (d, *J =* 8.0 Hz, 1H), 8.17 (d, *J =* 8.0 Hz, 1H), 8.15 (s, 1H), 8.07 (d, *J =* 7.6 Hz, 1H), 7.84 (t, *J =* 7.6 Hz, 1H), 7.77 (t, *J =* 7.6 Hz, 1H), 7.12 (d, *J =* 7.6 Hz, 1H), 7.06 (d, *J =* 5.6 Hz, 1H), 4.12 (q, *J =* 7.1 Hz, 2H), 2.79–2.86 (m, 2H), 2.20–2.27 (m, 2H), 2.06–2.17 (m, 1H), 1.90–2.00 (m, 1H), 1.09 (t, *J =* 7.2 Hz, 3H). ^13^C-NMR (DMSO-*d*_6_, 100 MHz) δ: 172.31, 150.24, 147.90, 145.83, 141.56, 132.94, 131.68, 131.52, 131.04, 128.93, 128.04, 125.33, 125.06, 124.78, 120.76, 117.62, 107.94, 61.47, 50.89, 33.10, 31.74, 16.20, 13.77.

*Ethyl 2-((1-((2-thiophenyl)methyl)-1H-imidazol-2-yl)thio)-2-methylpropionate* (**6w**): Oil; 16.78 g (89%). ^1^H-NMR (DMSO-*d*_6_, 400 MHz) δ: 7.44–7.46 (m, 2H), 7.05–7.06 (m, 2H), 6.96–6.98 (m, 1H), 5.40 (s, 2H), 4.03 (q, *J =* 7.2 Hz, 2H), 1.46 (s, 6H), 1.12 (t, *J =* 7.2 Hz, 3H). ^13^C-NMR (DMSO-*d*_6_, 100 MHz) δ: 172.56, 139.55, 136.03, 130.07, 126.97, 126.73, 126.27, 123.13, 61.00, 52.80, 44.26, 25.56, 13.70.

*Ethyl 1-((1-((2-thiophenyl)methyl)-1H-imidazol-2-yl)thio)cyclobutanecarboxylate* (**6x**): Oil; 15.86 g (82%). ^1^H-NMR (DMSO-*d*_6_, 400 MHz) δ: 7.46 (dd, *J =* 1.2 Hz and 5.2 Hz, 1H), 7.06 (d, *J =* 3.6 Hz, 1H), 7.03 (d, *J =* 0.8 Hz, 1H), 6.96–6.99 (m, 1H), 5.38 (s, 2H), 4.05 (q, *J =* 7.1 Hz, 2H), 2.51–2.56 (m, 2H), 2.24–2.31 (m, 2H), 2.04–2.11 (m, 1H), 1.78–1.85 (m, 1H), 1.11 (t, *J =* 7.2 Hz, 3H). ^13^C-NMR (DMSO-*d*_6_, 100 MHz) δ: 172.31, 139.47, 136.49, 129.99, 126.98, 126.75, 126.30, 122.89, 60.94, 54.26, 44.21, 31.21, 15.27, 13.75.

*Methyl 2-((4-((5-chloro-2-thiophenyl)methyl)-4H-1,2,4-triazol-3-yl)thio)acetate* (**6ha**): Oil; 14.22 g (78%). ^1^H-NMR (DMSO-*d*_6_, 400 MHz) δ: 8.69 (s, 1H), 7.04 (d, *J =* 4.0 Hz, 1H), 7.01 (d, *J =* 4.0 Hz, 1H), 5.36 (s, 2H), 4.07 (s, 2H), 3.63 (s, 3H). ^13^C-NMR (DMSO-*d*_6_, 100 MHz) δ: 168.62, 147.94, 145.31, 136.85, 128.88, 127.79, 126.87, 52.41, 42.32, 34.57.

*Methyl 2-((4-((5-methyl-2-thiophenyl)methyl)-4H-1,2,4-triazol-3-yl)thio)acetate* (**6hb**): Oil; 12.75 g (75%). ^1^H-NMR (DMSO-*d*_6_, 400 MHz) δ: 8.66 (s, 1H), 6.91 (d, *J =* 3.2 Hz, 1H), 6.68–6.69 (m, 1H), 5.31 (s, 2H), 4.05 (s, 2H), 3.63 (s, 3H), 2.38 (s, 3H). ^13^C-NMR (DMSO-*d*_6_, 100 MHz) δ: 168.67, 147.88, 145.27, 140.56, 135.17, 127.67, 125.31, 52.40, 42.45, 34.58, 14.87.

*Methyl 2-((4-((5-ethyl-2-thiophenyl)methyl)-4H-1,2,4-triazol-3-yl)thio)acetate* (**6hc**): Colorless oil; 12.85 g (72%). ^1^H-NMR (DMSO-*d*_6_, 400 MHz) δ: 8.69 (s, 1H), 6.94 (d, *J =* 3.2 Hz, 1H), 6.71 (d, *J =* 3.2 Hz, 1H), 5.34 (s, 2H), 4.06 (s, 2H), 3.63 (s, 3H), 2.74 (q, *J =* 7.5 Hz, 2H), 1.18 (t, *J =* 7.6 Hz, 3H). ^13^C-NMR (DMSO-*d*_6_, 100 MHz) δ: 168.67, 148.06, 147.92, 145.31, 134.86, 127.47, 123.52, 52.39, 42.53, 34.62, 22.74, 15.64.

*Methyl 2-((4-((3-methyl-2-thiophenyl)methyl)-4H-1,2,4-triazol-3-yl)thio)acetate* (**6hd**): Colorless oil; 13.26 g (78%). ^1^H-NMR (DMSO-*d*_6_, 400 MHz) δ: 8.62 (s, 1H), 7.42 (d, *J =* 5.2 Hz, 1H), 6.88 (d, *J =* 5.2 Hz, 1H), 5.33 (s, 2H), 4.03 (s, 2H), 3.62 (s, 3H), 2.25 (s, 3H). ^13^C-NMR (DMSO-*d*_6_, 100 MHz) δ: 168.67, 147.90, 145.33, 136.17, 130.70, 130.28, 125.29, 52.39, 40.79, 34.65, 13.45.

*Methyl 2-((4-((4-methyl-2-thiophenyl)methyl)-4H-1,2,4-triazol-3-yl)thio)acetate* (**6he**): Colorless oil; 11.39 g (67%). ^1^H-NMR (DMSO-*d*_6_, 400 MHz) δ: 8.68 (s, 1H), 7.08 (s, 1H), 6.92 (s, 1H), 5.34 (s, 2H), 4.05 (s, 2H), 3.62 (s, 3H), 2.15 (s, 3H). ^13^C-NMR (DMSO-*d*_6_, 100 MHz) δ: 168.66, 147.93, 145.35, 137.45, 137.14, 129.60, 121.85, 52.40, 42.40, 34.62, 15.24.

*Methyl 2-((4-((3-thiophenyl)methyl)-4H-1,2,4-triazol-3-yl)thio)acetate* (**6hf**): Oil; 10.99 g (68%). ^1^H-NMR (DMSO-*d*_6_, 400 MHz) δ: 8.68 (s, 1H), 7.55–7.57 (m, 1H), 7.42 (d, *J =* 2.0 Hz, 1H), 7.04–7.05 (m, 1H), 5.19 (s, 2H), 4.04 (s, 2H), 3.62 (s, 3H). ^13^C-NMR (DMSO-*d*_6_, 100 MHz) δ: 168.69, 148.02, 145.56, 136.25, 127.50, 126.96, 124.08, 52.40, 42.83, 34.41.

*Methyl 2-((4-((5-phenyl-2-thiophenyl)methyl)-4H-1,2,4-triazol-3-yl)thio)acetate* (**6hg**): White solid; 17.00 g (82%); m.p. 104–106 °C. ^1^H-NMR (DMSO-*d*_6_, 400 MHz) δ: 8.74 (s, 1H), 7.59–7.61 (m, 2H), 7.38–7.41 (m, 3H), 7.28–7.32 (m, 1H), 7.13 (d, *J =* 4.0 Hz, 1H), 5.43 (s, 2H), 4.07 (s, 2H), 3.63 (s, 3H). ^13^C-NMR (DMSO-*d*_6_, 100 MHz) δ: 168.66, 148.01, 145.39, 144.18, 137.10, 133.20, 129.11, 128.95, 127.89, 125.26, 123.54, 52.41, 42.50, 34.61.

*Methyl 2-((4-((benzo[b]thiophen-2-yl)methyl)-4H-1,2,4-triazol-3-yl)thio)acetate* (**6hh**): White solid; 12.46 g (65%); m.p. 84–86 °C. ^1^H-NMR (DMSO-*d*_6_, 400 MHz) δ: 8.77 (s, 1H), 7.92–7.94 (m, 1H), 7.82–7.84 (m, 1H), 7.34–7.38 (m, 3H), 5.55 (s, 2H), 4.06 (s, 2H), 3.61 (s, 3H) . ^13^C-NMR (DMSO-*d*_6_, 100 MHz) δ: 168.64, 148.27, 145.56, 139.25, 138.90, 138.74, 124.81, 124.68, 123.87, 123.84, 122.56, 52.41, 43.19, 34.63.

*Methyl 2-((4-((dibenzo[b,d]thiophen-4-yl)methyl)-4H-1,2,4-triazol-3-yl)thio)acetate* (**6hi**): White solid; 15.30 g (69%); m.p. 144–145 °C. ^1^H-NMR (DMSO-*d*_6_, 400 MHz) δ: 8.76 (s, 1H), 8.37–8.41 (m, 2H), 8.04–8.07 (m, 1H), 7.53–7.57 (m, 3H), 7.26 (d, *J =* 7.2 Hz, 1H), 5.52 (s, 2H), 4.01 (s, 2H), 3.60 (s, 3H). ^13^C-NMR (DMSO-*d*_6_, 100 MHz) δ: 168.60, 148.76, 146.23, 138.04, 136.78, 135.90, 134.81, 129.46, 127.39, 126.17, 125.22, 124.98, 122.96, 122.23, 121.98, 52.37, 46.76, 34.52.

#### 3.2.8. General Procedure for the Synthesis of Brominated Esters **7a**–**7s**, **7w**–**7x**, **7ha**–**7hd**, **7′he**, **7hf**–**7hg**, **7′hh** and **7hi**

A mixture of a substrate to be brominated **6a**–**6x** or **6ha**–**6hi** (40 mmol) and a brominating reagent NBS or DBDMH (48 mmol) in a suitable solvent MeCN or CH_2_Cl_2_ (100 mL) was stirred at a suitable temperature until the completion of reaction as indicated by TLC analysis (typically within 12 h). The brominating agent, solvent and temperature are specified in Scheme 1, Scheme 2, Scheme 3, Scheme 4 and Scheme 5.

The reaction mixture was poured into ice-water (200 mL), and the resulting mixture was extracted with CH_2_Cl_2_ (100 mL × 3). The combined extracts were washed successively with 5% aqueous Na_2_S_2_O_3_ (100 mL), saturated aqueous Na_2_CO_3_ (100 mL × 5) and 5% brine (100 mL), dried (Na_2_SO_4_) and evaporated on a rotary evaporator to give a residue, which was purified by column chromatography to afford the pure product **7a**–**7s**, **7w**–**7x**, **7ha**–**7hd**, **7′he**, **7hf**–**7hg**, **7′hh** or **7hi**.

*Methyl 2-((5-bromo-4-ethyl-4H-1,2,4-triazol-3-yl)thio)acetate* (**7a**): White solid; 5.54 g (52%); m.p. 86–88 °C. ^1^H-NMR (DMSO-*d*_6_, 400 MHz) δ: 4.04 (s, 2H), 3.64 (s, 3H), 3.54 (s, 3H). ^13^C-NMR (DMSO-*d*_6_, 100 MHz) δ: 168.62, 150.98, 130.88, 52.46, 34.54, 31.90.

*Methyl 2-((5-bromo-4-n-propyl-4H-1,2,4-triazol-3-yl)thio)acetate* (**7b**): White solid; 9.19 g (82%); m.p. 56–58 °C. ^1^H-NMR (DMSO-*d*_6_, 400 MHz) δ: 4.10 (s, 2H), 3.98 (q, *J =* 7.2 Hz, 2H), 3.64 (s, 3H), 1.25 (t, *J =* 7.2 Hz, 3H). ^13^C-NMR (DMSO-*d*_6_, 100 MHz) δ: 168.53, 150.45, 129.71, 52.47, 40.57, 34.41, 14.38.

*Methyl 2-((5-bromo-4-(cyclohexylmethyl)-4H-1,2,4-triazol-3-yl)thio)acetate* (**7c**): Colorless oil; 10.03 g (72%); ^1^H-NMR (DMSO-*d*_6_, 400 MHz) δ: 4.11 (s, 2H), 3.77 (d, *J =* 7.2 Hz, 2H), 3.64 (s, 3H), 1.61–1.78 (m, 4H), 1.49–1.52 (m, 2H), 1.11–1.18 (m, 3H), 0.95–1.04 (m, 2H). ^13^C-NMR (DMSO-*d*_6_, 100 MHz) δ: 168.46, 151.27, 130.47, 52.46, 50.82, 37.40, 34.33, 29.78, 25.53, 24.98.

*Methyl 2-((-4-(1-adamantylmethyl)-5-bromo-4H-1,2,4-triazol-3-yl)thio)acetate* (**7d**): White solid; 10.05 g (69%); m.p. 120–122 °C. ^1^H-NMR (DMSO-*d*_6_, 400 MHz) δ: 4.08 (s, 2H), 3.67 (s, 2H), 3.63 (s, 3H), 1.95 (s, 3H), 1.63–1.66 (m, 3H), 1.55–1.58 (m, 3H), 1.52–1.53 (m, 6H). ^13^C-NMR (DMSO-*d*_6_, 100 MHz) δ: 168.53, 152.52, 131.40, 56.34, 52.45, 40.20, 35.86, 35.43, 35.20, 27.50.

*Methyl 2-((4-benzyl-5-bromo-4H-1,2,4-triazol-3-yl)thio)acetate* (**7e**): White solid; 8.90 g (65%); m.p. 87–89 °C. ^1^H-NMR (DMSO-*d*_6_, 400 MHz) δ: 7.31–7.40 (m, 3H), 7.15–7.17 (m, 2H), 5.21 (s, 2H), 4.08 (s, 2H), 3.63 (s, 3H). ^13^C-NMR (DMSO-*d*_6_, 100 MHz) δ: 168.41, 151.44, 134.43, 130.64, 128.87, 128.13, 126.79, 52.48, 48.11, 34.25.

*Methyl 2-((5-bromo-4-(3-pyridinylmethyl)-4H-1,2,4-triazol-3-yl)thio)acetate* (**7f**): White solid; 7.55 g (55%); m.p. 68–70 °C. ^1^H-NMR (DMSO-*d*_6_, 400 MHz) δ: 8.54–8.56 (m, 1H), 8.51 (d, *J =* 1.6 Hz, 1H), 7.57–7.59 (m, 1H), 7.41–7.44 (m, 1H), 5.28 (s, 2H), 4.11 (s, 2H), 3.63 (s, 3H). ^13^C-NMR (DMSO-*d*_6_, 100 MHz) δ: 168.41, 151.45, 149.16, 148.17, 135.12, 130.56, 130.44, 123.98, 52.51, 45.89, 34.32.

*Methyl 2-((5-bromo-4-(2-furanylmethyl)-4H-1,2,4-triazol-3-yl)thio)acetate* (**7g**): White solid; 8.24 g (62%); m.p. 78–80 °C. ^1^H-NMR (DMSO-*d*_6_, 400 MHz) δ: 7.66 (d, *J =* 1.2 Hz, 1H), 6.50 (d, *J =* 3.2 Hz, 1H), 6.45–6.47 (m, 1H), 5.22 (s, 2H), 4.08 (s, 2H), 3.63 (s, 3H). ^13^C-NMR (DMSO-*d*_6_, 100 MHz) δ: 168.48, 151.22, 147.06, 143.89, 130.33, 110.79, 109.97, 52.48, 41.79, 34.44.

*Methyl 2-((5-bromo-4-(2-thiophenylmethyl)-4H-1,2,4-triazol-3-yl)thio)acetate* (**7h**): White solid; 10.86 g (78%); m.p. 81–83 °C. ^1^H-NMR (DMSO-*d*_6_, 400 MHz) δ: 7.54 (dd, *J =* 1.2 Hz and 4.8 Hz, 1H), 7.13–7.14 (m, 1H), 7.03 (dd, *J =* 3.6 Hz and 4.8 Hz, 1H), 5.38 (s, 2H), 4.10 (s, 2H), 3.63 (s, 3H). ^13^C-NMR (DMSO-*d*_6_, 100 MHz) δ: 168.42, 150.97, 136.35, 130.11, 127.90, 127.25, 127.18, 52.49, 43.46, 34.48.

*Methyl 2-((4-(biphenyl-4-methyl)-5-bromo-4H-1,2,4-triazol-3-yl)thio)acetate* (**7i**): White solid; 10.88 g (65%); m.p. 140–142 °C. ^1^H-NMR (DMSO-*d*_6_, 400 MHz) δ: 7.64–7.69 (m, 4H), 7.44–7.47 (m, 2H), 7.36 (t, *J =* 7.4 Hz, 1H), 7.25 (d, *J =* 8.4 Hz, 2H), 5.26 (s, 2H), 4.11 (s, 2H), 3.64 (s, 3H). ^13^C-NMR (DMSO-*d*_6_, 100 MHz) δ: 168.43, 151.47, 139.99, 139.34, 133.56, 130.66, 128.91, 127.62, 127.42, 127.14, 126.64, 52.50, 47.84, 34.27.

*Methyl 2-((5-bromo-4-(diphenylmethyl)-4H-1,2,4-triazol-3-yl)thio)acetate* (**7j**): Colorless oil; 9.54 g (57%); ^1^H-NMR (DMSO-*d*_6_, 400 MHz) δ: 7.32–7.40 (m, 6H), 7.21–7.25 (m, 4H), 6.93 (s, 1H), 3.95 (s, 2H), 3.55 (s, 3H). 

*Methyl 2-((4-(benzo[b]furan-5-methyl)-5-bromo-4H-1,2,4-triazol-3-yl)thio)acetate* (**7k**): White solid; 10.40 g (68%); m.p. 89–91 °C. ^1^H-NMR (DMSO-*d*_6_, 400 MHz) δ: 8.01 (d, *J =* 2.0 Hz, 1H), 7.61 (d, *J =* 8.4 Hz, 1H), 7.45 (d, *J =* 0.8 Hz, 1H), 7.17 (dd, *J =* 1.6 Hz and 8.8 Hz, 1H), 6.96–6.97 (m, 1H), 5.30 (s, 2H), 4.09 (s, 2H), 3.62 (s, 3H). ^13^C-NMR (DMSO-*d*_6_, 100 MHz) δ: 168.44, 153.87, 151.44, 146.92, 130.60, 129.13, 127.60, 123.38, 119.87, 111.69, 106.73, 52.48, 48.24, 34.25.

*Methyl 2-((5-bromo-4-((4-cyano-1-naphthyl)methyl)-4H-1,2,4-triazol-3-yl)thio)acetate* (**7l**): White solid; 10.85 g (65%); m.p. 120–122 °C. ^1^H-NMR (DMSO-*d*_6_, 400 MHz) δ: 8.37 (d, *J =* 8.0 Hz, 1H), 8.20–8.23 (m, 1H), 8.12 (d, *J =* 7.6 Hz, 1H), 7.86–7.94 (m, 2H), 6.70 (d, *J =* 7.6 Hz, 1H), 5.88 (s, 2H), 4.09 (s, 2H), 3.61 (s, 3H). ^13^C-NMR (DMSO-*d*_6_, 100 MHz) δ: 168.35, 151.95, 136.65, 132.94, 131.44, 131.26, 129.46, 129.26, 128.51, 125.12, 124.11, 121.74, 117.21, 109.24, 52.50, 46.07, 34.42.

*Methyl 2-((5-bromo-4-(2-naphthylmethyl)-4H-1,2,4-triazol-3-yl)thio)acetate* (**7m**): White solid; 9.41 g (60%); m.p. 91–93 °C. ^1^H-NMR (DMSO-*d*_6_, 400 MHz) δ: 7.88–7.96 (m, 3H), 7.64 (s, 1H), 7.51–7.55 (m, 2H), 7.34 (dd, *J =* 1.6 Hz and 8.4 Hz, 1H), 5.39 (s, 2H), 4.09 (s, 2H), 3.61 (s, 3H). ^13^C-NMR (DMSO-*d*_6_, 100 MHz) δ: 168.44, 151.57, 132.63, 132.42, 131.94, 130.74, 128.69, 127.74, 127.58, 126.64, 126.45, 125.63, 124.48, 52.46, 48.33, 34.30.

*Methyl 2-((5-bromo-4-(quinoline-6-methyl)-4H-1,2,4-triazol-3-yl)thio)acetate* (**7n**): White solid; 9.75 g (62%); m.p. 63–66 °C. ^1^H-NMR (DMSO-*d*_6_, 400 MHz) δ: 8.91 (dd, *J =* 1.6 Hz and 4.4 Hz, 1H), 8.36 (d, *J =* 8.8 Hz, 1H), 8.05 (d, *J =* 8.8 Hz, 1H), 7.67 (s, 1H), 7.60 (d, *J =* 2.0 Hz and 8.8 Hz, 1H), 7.53–7.56 (m, 1H), 5.44 (s, 2H), 4.09 (s, 2H), 3.61 (s, 3H). ^13^C-NMR (DMSO-*d*_6_, 100 MHz) δ: 168.45, 151.60, 150.97, 147.18, 135.98, 132.65, 130.80, 129.76, 128.08, 127.63, 125.79, 122.00, 52.48, 48.08, 34.39.

*Ethyl 2-((4-((5-bromo-4-cyano-1-naphthyl)methyl)-4H-1,2,4-triazol-3-yl)thio)-2-methylpropionate* (**7o**): White foam; 11.94 g (65%). ^1^H-NMR (DMSO-*d*_6_, 400 MHz) δ: 8.38–8.40 (m, 1H), 8.20–8.22 (m, 1H), 8.07 (d, *J =* 7.6 Hz, 1H), 7.86–7.94 (m, 2H), 6.50 (d, *J =* 7.2 Hz, 1H), 5.89 (s, 2H), 4.07 (q, *J =* 7.2 Hz, 2H), 1.53 (s, 6H), 1.11 (t, *J =* 7.2 Hz, 3H). ^13^C-NMR (DMSO-*d*_6_, 100 MHz) δ: 171.82, 149.65, 136.92, 132.91, 132.33, 131.37, 129.46, 129.05, 128.53, 125.12, 124.01, 121.42, 117.15, 109.21, 61.51, 53.89, 46.19, 25.83, 13.66.

*Ethyl 1-((5-bromo-4-((4-cyano-1-naphthyl)methyl)-4H-1,2,4-triazol-3-yl)thio)cyclobutanecarboxylate* (**7p**): White solid; 9.22 g (52%); m.p. 131–133 °C. ^1^H-NMR (DMSO-*d*_6_, 400 MHz) δ: 8.38 (d, *J =* 7.6 Hz, 1H), 8.20–8.23 (m, 1H), 8.10 (d, *J =* 7.6 Hz, 1H), 7.86–7.94 (m, 2H), 6.56 (d, *J =* 7.6 Hz, 1H), 5.87 (s, 2H), 4.06 (q, *J =* 7.1 Hz, 2H), 2.59–2.66 (m, 2H), 2.24–2.31 (m, 2H), 2.03–2.10 (m, 1H), 1.84–1.91 (m, 1H), 1.10 (t, *J =* 7.2 Hz, 3H). ^13^C-NMR (DMSO-*d*_6_, 100 MHz) δ: 172.06, 150.94, 137.53, 133.61, 132.67, 132.11, 130.19, 129.82, 129.27, 125.85, 124.70, 122.24, 117.87,109.98, 62.15, 55.09, 46.89, 32.58, 16.34, 14.42.

*Methyl 2-((5-bromo-1-((4-cyano-1-naphthyl)methyl)-1H-imidazol-2-yl)thio)acetate* (**7q**): White solid; 8.33 g (50%); m.p. 105–107 °C. ^1^H-NMR (DMSO-*d*_6_, 400 MHz) δ: 8.43 (d, *J =* 8.0 Hz, 1H), 8.21 (d, *J =* 7.6 Hz, 1H), 8.10 (d, *J =* 7.6 Hz, 1H), 7.85–7.93 (m, 2H), 7.31 (s, 1H), 6.49 (d, *J =* 7.6 Hz, 1H), 5.85 (s, 2H), 3.93 (s, 2H), 3.58 (s, 3H). ^13^C-NMR (DMSO-*d*_6_, 100 MHz) δ: 168.83, 141.67, 138.26, 133.01, 131.40, 130.25, 129.39, 129.20, 128.45, 125.11, 124.07, 121.33, 117.32, 108.87, 104.95, 52.34, 46.30, 35.11.

*Ethyl 2-((5-bromo-1-((4-cyano-1-naphthyl)methyl)-1H-imidazol-2-yl)thio)-2-methylpropionate* (**7r**): White solid; 10.08 g (55%); m.p. 125–127 °C. ^1^H-NMR (DMSO-*d*_6_, 400 MHz) δ: 8.43 (d, *J =* 7.6 Hz, 1H), 8.20 (d, *J =* 7.6 H, 1Hz), 8.07 (d, *J =* 7.6 Hz, 1H), 7.86–7.93 (m, 2H), 7.44 (s, 1H), 6.28 (d, *J =* 7.6 Hz, 1H), 5.89 (s, 2H), 4.05 (q, *J =* 7.1 Hz, 2H), 1.47 (s, 6H), 1.10 (t, *J =* 7.2 Hz, 3H). ^13^C-NMR (DMSO-*d*_6_, 100 MHz) δ: 172.28, 138.97, 138.57, 133.02, 131.33, 131.30, 129.42, 129.01, 128.50, 125.11, 123.97, 121.11, 117.28, 108.85, 106.69, 61.23, 53.31, 46.61, 25.56, 13.69.

*Ethyl 1-((5-bromo-1-((4-cyano-1-naphthyl)methyl)-1H-imidazol-2-yl)thio)cyclobutanecarboxylate* (**7s**): White solid; 9.78 g (52%); m.p. 102–105 °C. ^1^H-NMR (DMSO-*d*_6_, 400 MHz) δ: 8.42 (d, *J =* 8.0 Hz, 1H), 8.21 (d, *J =* 8.0 Hz, 1H), 8.09 (d, *J =* 7.6 Hz, 1H), 7.86–7.93 (m, 2H), 7.40 (s, 1H), 6.34 (d, *J =* 7.2 Hz, 1H), 5.87 (s, 2H), 4.04 (q, *J =* 7.1 Hz, 2H), 2.52–2.57 (m, 2H), 2.21–2.28 (m, 2H), 2.00–2.07 (m, 1H), 1.78–1.84 (m, 1H), 1.09 (t, *J =* 7.0 Hz, 3H). ^13^C-NMR (DMSO-*d*_6_, 100 MHz) δ: 171.92, 139.49, 138.47, 132.99, 131.33, 131.15, 129.42, 129.02, 128.50, 125.11, 123.93, 121.18, 117.27, 108.87, 106.31, 61.14, 54.38, 46.54, 31.39, 15.36, 13.71.

*Ethyl 2-((5-bromo-1-((2-thiophenyl)methyl)-1H-imidazol-2-yl)thio)-2-methylpropionate* (**7w**): Oil; 8.72 g (56%). ^1^H-NMR (DMSO-*d*_6_, 400 MHz) δ: 7.46–7.47 (m, 1H), 7.24 (s, 1H), δ: 7.007–7.013 (m, 1H), 6.97–6.99 (m, 1H), 5.42 (s, 2H), 4.03 (q, *J =* 7.2 Hz, 2H), 1.48 (s, 6H), 1.10 (t, *J =* 7.2 Hz, 3H). ^13^C-NMR (DMSO-*d*_6_, 100 MHz) δ: 172.31, 138.40, 137.84, 130.86, 126.97, 126.71, 126.46, 105.57, 61.15, 53.31, 43.86, 25.47, 13.64.

*Ethyl 1-((5-bromo-1-((2-thiophenyl)methyl)-1H-imidazol-2-yl)thio)cyclobutanecarboxylate* (**7x**): Colorless oil; 8.51 g (53%). ^1^H-NMR (DMSO-*d*_6_, 400 MHz) δ: 7.48 (dd, *J =* 0.8 Hz and 5.2 Hz, 1H), 7.20 (s, 1H), 7.03 (d, *J =* 2.8 Hz, 1H), 6.97–7.00 (m, 1H), 5.40 (s, 2H), 4.05 (q, *J =* 7.2 Hz, 2H), 2.51–2.59 (m, 2H), 2.24–2.31 (m, 2H), 2.06–2.13 (m, 1H), 1.79–1.87 (m, 1H), 1.10 (t, *J =* 7.2 Hz, 3H). ^13^C-NMR (DMSO-*d*_6_, 100 MHz) δ: 172.04, 138.40, 138.31, 130.73, 126.99, 126.80, 126.53, 105.17, 61.10, 54.52, 43.77, 31.26, 15.35, 13.71.

*Methyl 2-((5-bromo-4-((5-chloro-2-thiophenyl)methyl)-4H-1,2,4-triazol-3-yl)thio)acetate* (**7ha**): Colorless oil; 7.96 g (52%). ^1^H-NMR (DMSO-*d*_6_, 400 MHz) δ: 7.05 (d, *J =* 3.6 Hz, 1H), 7.00 (d, *J =* 4.0 Hz, 1H), 5.34 (s, 2H), 4.11 (s, 2H), 3.63 (s, 3H). 

*Methyl 2-((5-bromo-4-((5-methyl-2-thiophenyl)methyl)-4H-1,2,4-triazol-3-yl)thio)acetate* (**7hb**): White solid; 8.40 g (58%); m.p. 57–59 °C. ^1^H-NMR (DMSO-*d*_6_, 400 MHz) δ: 6.93 (d, *J =* 3.6 Hz, 1H), 6.69–6.70 (m, 1H), 5.28 (s, 2H), 4.09 (s, 2H), 3.64 (s, 3H), 2.39 (s, 3H). ^13^C-NMR (DMSO-*d*_6_, 100 MHz) δ: 168.44, 150.91, 140.83, 133.82, 130.03, 127.97, 125.27, 52.49, 43.68, 34.45, 14.85.

*Methyl 2-((5-bromo-4-((5-ethyl-2-thiophenyl)methyl)-4H-1,2,4-triazol-3-yl)thio)acetate* (**7hc**): Colorless oil; 9.03 g (60%). ^1^H-NMR (DMSO-*d*_6_, 400 MHz) δ: 6.95 (d, *J =* 3.6 Hz, 1H), 6.73 (d, *J =* 3.2 Hz, 1H), 5.29 (s, 2H), 4.09 (s, 2H), 3.62 (s, 3H), 2.75 (q, *J =* 7.5 Hz, 2H), 1.18 (t, *J =* 7.4 Hz, 3H). ^13^C-NMR (DMSO-*d*_6_, 100 MHz) δ: 168.44, 150.95, 148.35, 133.46, 130.03, 127.86, 123.49, 52.50, 43.75, 34.51, 22.73, 15.60.

*Methyl 2-((5-bromo-4-((3-methyl-2-thiophenyl)methyl)-4H-1,2,4-triazol-3-yl)thio)acetate* (**7hd**): White solid; 9.13 g (63%); m.p. 79–81 °C. ^1^H-NMR (DMSO-*d*_6_, 400 MHz) δ: 7.43 (d, *J =* 5.2 Hz, 1H), 6.88 (d, *J =* 5.2 Hz, 1H), 5.31 (s, 2H), 4.05 (s, 2H), 3.63 (s, 3H), 2.29 (s, 3H). ^13^C-NMR (DMSO-*d*_6_, 100 MHz) δ: 168.41, 151.00, 135.95, 130.20, 125.37, 52.47, 42.50, 34.49, 13.74.

*Methyl 2-((4-((5-bromo-4-methyl-2-thiophenyl)methyl)-4H-1,2,4-triazol-3-yl)thio)acetate* (**7′he**) White solid; 8.11 g (56%); m.p. 75–77 °C. ^1^H-NMR (DMSO-*d*_6_, 400 MHz) δ: 8.68 (s, 1H), 6.91 (s, 1H), 5.32 (s, 2H), 4.06 (s, 2H), 3.63 (s, 3H), 2.09 (s, 3H). ^13^C-NMR (DMSO-*d*_6_, 100 MHz) δ: 168.63, 147.94, 145.33, 137.37, 137.18, 129.84, 108.88, 52.41, 42.30, 34.59, 14.75.

*Methyl 2-((5-bromo-4-((3-thiophenyl)methyl)-4H-1,2,4-triazol-3-yl)thio)acetate* (**7hf**): Colorless oil; 6.96 g (50%); ^1^H-NMR (DMSO-*d*_6_, 400 MHz) δ: 7.57–7.59 (m, 1H), 7.38 (d, *J =* 1.6 Hz, 1H), 7.01 (dd, *J =* 1.2 Hz and 5.2 Hz, 1H), 5.18 (s, 2H), 4.09 (s, 2H), 3.63 (s, 3H). ^13^C-NMR (DMSO-*d*_6_, 100 MHz) δ: 168.46, 151.12, 134.99, 130.27, 127.69, 126.47, 123.94, 52.49, 44.08, 34.27.

*Methyl 2-((5-bromo-4-((5-phenyl-2-thiophenyl)methyl)-4H-1,2,4-triazol-3-yl)thio)acetate* (**7hg**): White solid; 9.00 g (53%); m.p. 78–80 °C. ^1^H-NMR (DMSO-*d*_6_, 400 MHz) δ: 7.59–7.61 (m, 2H), 7.38–7.41 (m, 3H), 7.31 (t, *J =* 7.4 Hz, 1H), 7.14 (d, *J =* 7.6 Hz, 1H), 5.40 (s, 2H), 4.12 (s, 2H), 3.63 (s, 3H). ^13^C-NMR (DMSO-*d*_6_, 100 MHz) δ: 168.43, 151.03, 144.36, 135.72, 133.05, 130.16, 129.21, 129.12, 127.98, 125.31, 123.49, 52.49, 43.70, 34.49.

*Methyl 2-((4-((3-bromobenzo[b]thiophen-2-yl)methyl)-4H-1,2,4-triazol-3-yl)thio)acetate* (**7′hh**): White solid; 10.36 g (65%); m.p. 94–96 °C. ^1^H-NMR (DMSO-*d*_6_, 400 MHz) δ: 8.77 (s, 1H), 8.03 (d, *J =* 8.0 Hz, 1H), 7.77 (d, *J =* 7.6 Hz, 1H), 7.47–7.56 (m, 2H), 5.60 (s, 2H), 4.04 (s, 2H), 3.60 (s, 3H). ^13^C-NMR (DMSO-*d*_6_, 100 MHz) δ: 169.28, 149.14, 146.39, 137.80, 137.74, 134.48, 127.01, 126.55, 123.96, 123.49, 108.30, 53.12, 43.52, 35.41.

*Methyl 2-((5-bromo-4-((dibenzo[b,d]thiophen-4-yl)methyl)-4H-1,2,4-triazol-3-yl)thio)acetate* (**7hi**): White solid; 9.33 g (52%); m.p. 120–122 °C. ^1^H-NMR (DMSO-*d*_6_, 400 MHz) δ: 8.37–8.42 (m, 2H), 8.06–8.08 (m, 1H), 7.52–7.57 (m, 3H), 7.12 (d, *J =* 7.6 Hz, 1H), 5.49 (s, 2H), 4.04 (s, 2H), 3.60 (s, 3H). ^13^C-NMR (DMSO-*d*_6_, 100 MHz) δ: 168.39, 151.92, 138.04, 136.17, 135.98, 134.75, 131.25, 128.48, 127.49, 125.30, 125.24, 125.07, 123.00, 122.29, 121.97, 52.47, 47.59, 34.40.

#### 3.2.9. General Procedure for the Synthesis of Brominated Esters **7he** and **7hh**

To a stirred solution of **7′he** or **7′hh** (20 mmol) in MeCN (100 mL) at room temperature was added NBS (3.56 g, 20 mmol), and the resulting mixture was stirred until completion of the reaction as indicated by TLC analysis (typically within 12h). The reaction mixture was poured into ice-water (200 mL), and the resulting mixture was extracted with CH_2_Cl_2_ (200 mL × 3). The combined extracts were washed successively with 5% aqueous Na_2_S_2_O_3_ (100 mL), saturated aqueous Na_2_CO_3_ (100 mL × 5) and 5% brine (100 mL), dried (Na_2_SO_4_) and evaporated on a rotary evaporator to give a residue, which was purified by column chromatography to afford the pure product **7he** or **7hh**.

*Methyl 2-((5-bromo-4-((5-bromo-4-methyl-2-thiophenyl)methyl)-4H-1,2,4-triazol-3-yl)thio)acetate* (**7he**): White solid; 5.92 g (62%); m.p. 91–93 °C. ^1^H-NMR (DMSO-*d*_6_, 400 MHz) δ: 6.91 (s, 1H), 5.29 (s, 2H), 4.11 (s, 2H), 3.63 (s, 3H), 2.09 (s, 3H). ^13^C-NMR (DMSO-*d*_6_, 100 MHz) δ: 168.40, 150.98, 137.21, 136.05, 130.12, 129.94, 109.04, 52.49, 43.48, 34.47, 14.73.

*Methyl 2-((5-bromo-4-((3-bromobenzo[b]thiophen-2-yl)methyl)-4H-1,2,4-triazol-3-yl)thio)acetate* (**7hh**): White solid; 5.64 g (66%); m.p. 81–83 °C. ^1^H-NMR (DMSO-*d*_6_, 400 MHz) δ: 8.03 (d, *J =* 0.8 Hz, 1H), 7.79 (d, *J =* 8.0 Hz, 1H), 7.53–7.57 (m, 1H), 7.47–7.51 (m, 1H), 5.56 (s, 2H), 4.09 (s, 2H), 3.62 (s, 3H). ^13^C-NMR (DMSO-*d*_6_, 100 MHz) δ: 168.31, 151.42, 136.97, 136.83, 132.93, 130.58, 126.36, 125.90, 123.24, 122.78, 107.56, 52.48, 44.25, 34.51.

#### 3.2.10 General Procedure for the Synthesis of Acids **8a**–**8x** and **8ha**–**8hi**

To a stirred solution of **6t**–**6v**, **7a**–**7s**, **7w**–**7x** or **7ha**–**7hi** (5 mmol) in MeOH (20 mL) at room temperature was added an aqueous solution of LiOH prepared by dissolving LiOH·H_2_O (0.42 g, 10 mmol) in a minimal amount of water, and the resulting mixture was stirred at room temperature until the reaction completed as indicated by TLC analysis (typically within 5 h). The reaction mixture was poured into ice-water (50 mL), and the resulting mixture was acidified to pH 4-5 (for **6t**-**6v**, **7f** and **7n**) or 1–2 (for others) with concentrated hydrochloric acid before the extraction with CH_2_Cl_2_ (50 mL × 3). The combined extracts were washed with water (50 mL), dried (Na_2_SO_4_) and evaporated on a rotary evaporator to afford the crude product as a residue, which was purified by trituration with EtOAc/*n*-hexane to yield the pure product **8a**–**8x** or **8ha**–**8hi**.

*2-((5-Bromo-4-ethyl-4H-1,2,4-triazol-3-yl)thio)acetic acid* (**8a**): White solid; 0.98 g (78%); m.p. 136–138 °C. ^1^H-NMR (DMSO-*d*_6_, 400 MHz) δ: 12.95 (brs, 1H), 3.95 (s, 2H), 3.53 (s, 3H).

*2-((5-Bromo-4-n-propyl-4H-1,2,4-triazol-3-yl)thio)acetic acid* (**8b**): White solid; 0.10 g (75%); m.p. 143–145 °C. ^1^H-NMR (DMSO-*d*_6_, 400 MHz) δ: 12.94 (brs, 1H), 4.01 (s, 2H), 3.98 (q, *J =* 7.3 Hz, 2H), 1.25 (t, *J =* 7.2 Hz, 3H).

*2-((5-Bromo-4-(cyclohexylmethyl)-4H-1,2,4-triazol-3-yl)thio)acetic acid* (**8c**): White solid; 1.17 g (70%); m.p. 155–157 °C. ^1^H-NMR (DMSO-*d*_6_, 400 MHz) δ: 13.02 (brs, 1H), 4.03 (s, 2H), 3.76 (d, *J =* 7.6 Hz, 2H), 1.61–1.79 (m, 4H), 1.49–1.52 (m, 2H), 1.11–1.20 (m, 3H), 0.95–1.04 (m, 2H).

*2-((4-(1-Adamantylmethyl)-5-bromo-4H-1,2,4-triazol-3-yl)thio)acetic acid* (**8d**): White solid; 1.82 g (94%); m.p. 169–171 °C. ^1^H-NMR (DMSO-*d*_6_, 400 MHz) δ: 12.92 (brs, 1H), 4.00 (s, 2H), 3.66 (s, 2H), 1.95 (s, 3H), 1.63–1.66 (m, 3H), 1.54–1.58 (m, 3H), 1.52–1.53 (m, 6H).

*2-((4-Benzyl-5-bromo-4H-1,2,4-triazol-3-yl)thio)acetic acid* (**8e**): White solid; 2.05 g (78%); m.p. 160–162 °C. ^1^H-NMR (DMSO-*d*_6_, 400 MHz) δ: 12.97 (brs, 1H), 7.31–7.40 (m, 3H), 7.16–7.18 (m, 2H), 5.20 (s, 2H), 4.01 (s, 2H).

*2-((5-Bromo-4-(3-pyridinylmethyl)-4H-1,2,4-triazol-3-yl)thio)acetic acid* (**8f**): White solid; 1.25 g (76%); m.p. 173 °C (dec). ^1^H-NMR (DMSO-*d*_6_, 400 MHz) δ: 13.03 (brs, 1H), 8.54 (d, *J =* 4.0 Hz, 1H), 8.51 (d, *J =* 1.6 Hz, 1H), 7.58 (d, *J =* 8.0 Hz, 1H), 7.39–7.42 (m, 1H), 5.27 (s, 2H), 4.03 (s, 2H).

*2-((5-Bromo-4-(2-furanylmethyl)-4H-1,2,4-triazol-3-yl)thio)acetic acid* (**8g**): White solid; 1.24 g (78%); m.p. 113–116 °C. ^1^H-NMR (DMSO-*d*_6_, 400 MHz) δ: 12.97 (brs, 1H), 7.65 (s, 1H), 6.50 (d, *J =* 3.2 Hz, 1H), 6.45–6.46 (m, 1H), 5.21 (s, 2H), 4.00 (s, 2H).

*2-((5-Bromo-4-(2-thiophenylmethyl)-4H-1,2,4-triazol-3-yl)thio)acetic acid* (**8h**): White solid; 1.40 g (84%); m.p. 113–115 °C. ^1^H-NMR (DMSO-*d*_6_, 400 MHz) δ: 12.98 (brs, 1H), 7.54 (dd, *J =* 1.2 Hz and 5.2 Hz, 1H), 7.14 (d, *J =* 3.2 Hz, 1H), 7.01–7.03 (m, 1H), 5.38 (s, 2H), 4.02 (s, 2H).

*2-((4-(Biphenyl-4-methyl)-5-bromo-4H-1,2,4-triazol-3-yl)thio)acetic acid* (**8i**): White solid; 1.44 g (71%); m.p. 178–179 °C. ^1^H-NMR (DMSO-*d*_6_, 400 MHz) δ: 13.01 (brs, 1H), 7.64–7.69 (m, 4H), 7.45 (t, *J =* 7.6 Hz, 2H), 7.36 (t, *J =* 7.2 Hz, 1H), 7.26 (d, *J =* 8.4 Hz, 2H), 5.25 (s, 2H), 4.02 (s, 2H).

*2-((5-Bromo-4-(diphenylmethyl)-4H-1,2,4-triazol-3-yl)thio)acetic acid* (**8j**): White solid; 1.48 g (73%); m.p. 134–135 °C. ^1^H-NMR (DMSO-*d*_6_, 400 MHz) δ: 12.79 (brs, 1H), 7.32–7.40 (m, 6H), 7.23–7.25 (m, 4H), 6.93 (s, 1H), 3.88 (s, 2H).

*2-((4-(Benzo[b]furan-5-methyl)-5-bromo-4H-1,2,4-triazol-3-yl)thio)acetic acid* (**8k**): White solid; 1.51 g (82%); m.p. 171–173 °C. ^1^H-NMR (DMSO-*d*_6_, 400 MHz) δ: 12.97 (brs, 1H), 8.01 (d, *J =* 2.0 Hz, 1H), 7.61 (d, *J =* 8.4 Hz, 1H), 7.46 (s, 1H), 7.17–7.20 (m, 1H), 6.96 (d, *J =* 1.6 Hz, 1H), 5.29 (s, 2H), 4.02 (s, 2H).

*2-((5-Bromo-4-((4-cyano-1-naphthyl)methyl)-4H-1,2,4-triazol-3-yl)thio)acetic acid* (**8l**): White solid; 1.61 g (80%); m.p. 170–172 °C. ^1^H-NMR (DMSO-*d*_6_, 400 MHz) δ: 12.93 (brs, 1H), 8.37 (d, *J =* 7.6 Hz, 1H), 8.20–8.22 (m, 1H), 8.09 (d, *J =* 7.6 Hz, 1H), 7.85–7.93 (m, 2H), 6.71 (d, *J =* 7.6 Hz, 1H), 5.87 (s, 2H), 4.01 (s, 2H).

*2-((5-Bromo-4-(2-naphthylmethyl)-4H-1,2,4-triazol-3-yl)thio)acetic acid* (**8m**): White solid; 1.48 g (78%); m.p. 172–174 °C. ^1^H-NMR (DMSO-*d*_6_, 400 MHz) δ: 12.97 (brs, 1H), 7.88–7.95 (m, 3H), 7.65 (s, 1H), 7.51–7.55 (m, 2H), 7.35 (dd, *J =* 1.6 Hz and 7.6 Hz, 1H), 5.38 (s, 2H), 4.03 (s, 2H).

*2-((5-Bromo-4-(quinoline-6-methyl)-4H-1,2,4-triazol-3-yl)thio)acetic acid* (**8n**): White solid; 1.37 g (72%); m.p. 203 °C (dec). ^1^H-NMR (DMSO-*d*_6_, 400 MHz) δ: 12.98 (brs, 1H), 8.90–8.91 (m, 1H), 8.35 (d, *J =* 8.0 Hz, 1H), 8.04 (d, *J =* 8.4 Hz, 1H), 7.69 (s, 1H), 7.60–7.63 (m, 1H), 7.54 (dd, *J =* 4.0 Hz and 8.4 Hz, 1H), 5.43 (s, 2H), 4.03 (s, 2H).

*2-((4-((5-Bromo-4-cyano-1-naphthyl)methyl)-4H-1,2,4-triazol-3-yl)thio)-2-methylpropionic acid* (**8o**): White solid; 1.47 g (68%); m.p. 168–170 °C. ^1^H-NMR (DMSO-*d*_6_, 400 MHz) δ: 13.16 (brs, 1H), 8.35 (d, *J =* 7.6 Hz, 1H), 8.20–8.22 (m, 1H), 8.07 (d, *J =* 7.6 Hz, 1H), 7.86–7.94 (m, 2H), 6.53 (d, *J =* 7.6 Hz, 1H), 5.89 (s, 2H), 1.51 (s, 6H).

*1-((5-Bromo-4-((4-cyano-1-naphthyl)methyl)-4H-1,2,4-triazol-3-yl)thio)cyclobutanecarboxylic acid* (**8p**): White solid; 1.44 g (65%); m.p. 173–175 °C. ^1^H-NMR (DMSO-*d*_6_, 400 MHz) δ: 13.08 (brs, 1H), 8.34 (d, *J =* 8.0 Hz, 1H), 8.21 (d, *J =* 7.6 Hz, 1H), 8.08 (d, *J =* 7.6 Hz, 1H), 7.86–7.94 (m, 2H), 6.60 (d, *J =* 7.6 Hz, 1H), 5.86 (s, 2H), 2.57–2.65 (m, 2H), 2.24–2.31 (m, 2H), 1.99–2.07 (m, 1H), 1.86–1.92 (m, 1H).

*2-((5-Bromo-1-((4-cyano-1-naphthyl)methyl)-1H-imidazol-2-yl)thio)acetic acid* (**8q**): White solid; 1.45 g (72%); m.p. 180–182 °C. ^1^H-NMR (DMSO-*d*_6_, 400 MHz) δ: 12.84 (brs, 1H), 8.42 (d, *J =* 8.4 Hz, 1H), 8.20 (d, *J =* 7.6 Hz, 1H), 8.09 (d, *J =* 7.6 Hz, 1H), 7.85–7.93 (m, 2H), 7.30 (s, 1H), 6.51 (d, *J =* 7.6 Hz, 1H), 5.85 (s, 2H), 3.86 (s, 2H).

*2-((5-Bromo-1-((4-cyano-1-naphthyl)methyl)-1H-imidazol-2-yl)thio)-2-methylpropionic acid* (**8r**): White solid; 1.61 g (75%); m.p. 193–195 °C. ^1^H-NMR (DMSO-*d*_6_, 400 MHz) δ: 12.96 (brs, 1H), 8.39 (d, *J =* 7.6 Hz, 1H), 8.19–8.22 (m, 1H), 8.07 (d, *J =* 7.6 Hz, 1H), 7.86–7.93 (m, 2H), 7.43 (s, 1H), 6.33 (d, *J =* 7.6 Hz, 1H), 5.90 (s, 2H), 1.44 (s, 6H).

*1-((5-Bromo-1-((4-cyano-1-naphthyl)methyl)-1H-imidazol-2-yl)thio)cyclobutanecarboxylic acid* (**8s**): White solid; 1.50 g (68%); m.p. 210–213 °C. ^1^H-NMR (DMSO-*d*_6_, 400 MHz) δ: 12.96 (brs, 1H), 8.38 (d, *J =* 8.0 Hz, 1H), 8.21 (d, *J =* 8.4 Hz, 1H), 8.08 (d, *J =* 7.6 Hz, 1H), 7.85–7.93 (m, 2H), 7.40 (s, 1H), 6.39 (d, *J =* 7.6 Hz, 1H), 5.88 (s, 2H), 2.53–2.56 (m, 2H), 2.19–2.26 (m, 2H), 1.95–2.02 (m, 1H), 1.79–1.85 (m, 1H).

*2-((3-((4-Cyano-1-naphthyl)methyl)pyridin-4-yl)thio)acetic acid* (**8t**): White solid; 1.30 g (78%); m.p. 246–249 °C. ^1^H-NMR (DMSO-*d*_6_, 400 MHz) δ: 13.10 (brs, 1H), 8.38 (d, *J =* 5.2 Hz, 1H), 8.28 (d, *J =* 8.4 Hz, 1H), 8.17 (d, *J =* 8.0 Hz, 1H), 8.11 (s, 1H), 8.07 (d, *J =* 7.6 Hz, 1H), 7.84 (t, *J =* 7.4 Hz, 1H), 7.76 (t, *J =* 7.8 Hz, 1H), 7.36 (d, *J =* 5.2 Hz, 1H), 7.17 (d, *J =* 7.6 Hz, 1H), 4.56 (s, 2H), 4.01 (s, 2H).

*2-((3-((4-Cyano-1-naphthyl)methyl)pyridin-4-yl)thio)-2-methylpropionic acid* (**8u**) White soli; 1.30 g (72%); m.p. 175–177 °C. ^1^H-NMR (DMSO-*d*_6_, 400 MHz) δ: 13.11 (brs, 1H), 8.43 (d, *J =* 5.2 Hz, 1H), 8.26 (d, *J =* 8.4 Hz, 1H), 8.24 (s, 1H), 8.17 (d, *J =* 8.4 Hz, 1H), 8.06 (d, *J =* 7.2 Hz, 1H), 7.83 (t, *J =* 7.6 Hz, 1H), 7.77 (t, *J =* 7.6 Hz, 1H), 7.43 (d, *J =* 5.2 Hz, 1H), 7.09 (d, *J =* 7.2 Hz, 1H), 4.64 (s, 2H), 1.50 (s, 6H).

*1-((3-((4-Cyano-1-naphthyl)methyl)pyridin-4-yl)thio)cyclobutanecarboxylic acid* (**8v**): White solid; 1.22 g (65%); m.p. 210–212 °C. ^1^H-NMR (DMSO-*d*_6_, 400 MHz) δ: 13.10 (brs, 1H), 8.34 (d, *J =* 5.6 Hz, 1H), 8.26 (d, *J =* 8.4 Hz, 1H), 8.17 (d, *J =* 8.4 Hz, 1H), 8.10 (s, 1H), 8.08 (d, *J =* 7.6 Hz, 1H), 7.84 (t, *J =* 7.4 Hz, 1H), 7.76 (t, *J =* 7.6 Hz, 1H), 7.17 (d, *J =* 7.6 Hz, 1H), 7.11 (d, *J =* 5.6 Hz, 1H), 4.54 (s, 2H), 2.78–2.86 (m, 2H), 2.18–2.25 (m, 2H), 2.07–2.16 (m, 1H), 1.92–2.00 (m, 1H).

*2-((5-Bromo-1-((2-thiophenyl)methyl)-1H-imidazol-2-yl)thio)-2-methylpropionic acid* (**8w**): White solid; 1.48 g (82%); m.p. 153–155 °C. ^1^H-NMR (DMSO-*d*_6_, 400 MHz) δ: 12.92 (brs, 1H), 7.46–7.47 (m, 1H), 7.22 (s, 1H), 7.03 (d, *J =* 3.2 Hz, 1H), 6.97–6.99 (m, 1H), 5.44 (s, 2H), 1.44 (s, 6H).

*1-((5-Bromo-1-((2-thiophenyl)methyl)-1H-imidazol-2-yl)thio)cyclobutanecarboxylic acid* (**8x**): White solid; 1.40 g (75%); m.p. 164–166 °C. ^1^H-NMR (DMSO-*d*_6_, 400 MHz) δ: 12.96 (brs, 1H), 7.47–7.48 (m, 1H), 7.20 (s, 1H), 7.05 (d, *J =* 7.2 Hz, 1H), 6.99 (dd, *J =* 3.6 Hz and 4.8 Hz, 1H), 5.42 (s, 2H), 2.51–2.56 (m, 2H), 2.20–2.27 (m, 2H), 1.98–2.06 (m, 1H), 1.80–1.86 (m, 1H).

*2-((5-Bromo-4-((5-chloro-2-thiophenyl)methyl)-4H-1,2,4-triazol-3-yl)thio)acetic acid* (**8ha**): White solid; 1.25 g (68%); m.p. 142–143 °C. ^1^H-NMR (DMSO-*d*_6_, 400 MHz) δ: 12.95 (brs, 1H), 7.05 (d, *J =* 4.0 Hz, 1H), 7.01 (d, *J =* 4.0 Hz, 1H), 5.33 (s, 2H), 4.03 (s, 2H).

*2-((5-Bromo-4-((5-methyl-2-thiophenyl)methyl)-4H-1,2,4-triazol-3-yl)thio)acetic acid* (**8hb**): White solid; 1.31 g (75%); m.p. 119–120 °C. ^1^H-NMR (DMSO-*d*_6_, 400 MHz) δ: 12.98 (brs, 1H), 6.93 (d, *J =* 3.2 Hz, 1H), 6.69–6.70 (m, 1H), 5.28 (s, 2H), 4.01 (s, 2H), 2.38 (s, 3H).

*2-((5-Bromo-4-((5-ethyl-2-thiophenyl)methyl)-4H-1,2,4-triazol-3-yl)thio)acetic acid* (**8hc**): White solid; 1.41 g (78%); m.p. 119–120.5 °C. ^1^H-NMR (DMSO-*d*_6_, 400 MHz) δ: 12.98 (brs, 1H), 6.95 (d, *J =* 3.6 Hz, 1H), 6.73 (d, *J =* 7.2 Hz, 1H), 5.29 (s, 2H), 4.02 (s, 2H), 2.75 (q, *J =* 7.5 Hz, 2H), 1.18 (t, *J =* 7.4 Hz, 3H).

*2-((5-Bromo-4-((3-methyl-2-thiophenyl)methyl)-4H-1,2,4-triazol-3-yl)thio)acetic acid* (**8hd**): White solid; 1.45 g (83%); m.p. 111–112 °C. ^1^H-NMR (DMSO-*d*_6_, 400 MHz) δ: 12.97 (brs, 1H), 7.42 (d, *J =* 4.8 Hz, 1H), 6.88 (d, *J =* 5.2 Hz, 1H), 5.30 (s, 2H), 3.98 (s, 2H), 2.29 (s, 3H).

*2-((5-Bromo-4-((5-bromo-4-methyl-2-thiophenyl)methyl)-4H-1,2,4-triazol-3-yl)thio)acetic acid* (**8he**): White solid; 1.82 g (85%); m.p. 144–145 °C. ^1^H-NMR (DMSO-*d*_6_, 400 MHz) δ: 12.99 (brs, 1H), 6.92 (s, 1H), 5.29 (s, 2H), 4.03 (s, 2H), 2.09 (s, 3H).

*2-((5-Bromo-4-((3-thiophenyl)methyl)-4H-1,2,4-triazol-3-yl)thio)acetic acid* (**8hf**): White solid; 1.27 g (76%); m.p. 124–126 °C. ^1^H-NMR (DMSO-*d*_6_, 400 MHz) δ: 12.96 (brs, 1H), 7.56–7.58 (m, 1H), 7.39 (d, *J =* 2.0 Hz, 1H), 7.03 (dd, *J =* 0.8 Hz and 5.2 Hz, 1H), 5.17 (s, 2H), 4.01 (s, 2H).

*2-((5-Bromo-4-((5-phenyl-2-thiophenyl)methyl)-4H-1,2,4-triazol-3-yl)thio)acetic acid* (**8hg**): White solid; 1.48 g (72%); m.p. 126–128 °C. ^1^H-NMR (DMSO-*d*_6_, 400 MHz) δ: 13.02 (brs, 1H), 7.59–7.61 (m, 2H), 7.38–7.41 (m, 3H), 7.31 (t, *J =* 7.2 Hz, 1H), 7.15 (d, *J =* 3.6 Hz, 1H), 5.39 (s, 2H), 4.04 (s, 2H).

*2-((5-Bromo-4-((3-bromobenzo[b]thiophen-2-yl)methyl)-4H-1,2,4-triazol-3-yl)thio)acetic acid* (**8hh**): White solid; 1.81 g (78%); m.p. 153–155 °C. ^1^H-NMR (DMSO-*d*_6_, 400 MHz) δ: 12.98 (brs, 1H), 8.03 (d, *J =* 8.0 Hz, 1H), 7.79 (d, *J =* 7.6 Hz, 1H), 7.53–7.57 (m, 1H), 7.47–7.51 (m, 1H), 5.55 (s, 2H), 4.02 (s, 2H).

*2-((5-Bromo-4-((dibenzo[b,d]thiophen-4-yl)methyl)-4H-1,2,4-triazol-3-yl)thio)acetic acid* (**8hi**): White solid; 1.59 g (73%); m.p. 157 °C. ^1^H-NMR (DMSO-*d*_6_, 400 MHz) δ: 12.94 (brs, 1H), 8.37–8.42 (m, 2H), 8.06–8.08 (m, 1H), 7.52–7.57 (m, 3H), 7.13 (d, *J =* 7.2 Hz, 1H), 5.48 (s, 2H), 3.98 (s, 2H).

#### 3.2.11 General Procedure for the Synthesis of Sodium Salts **1a**–**1x** and **1ha**–**1hi**

To a stirred mixture of **8a**–**8x** or **8ha**–**8hi** (accurately weighted to four decimal places, 2 mmol) in MeOH (10 mL) was added an aqueous solution of NaOH prepared by dissolving NaOH (0.0800 g, 2 mmol) in a minimal amount of water, and the resulting mixture was stirred at room temperature until a clear solution was obtained (typically within 1 h). The reaction mixture was filtered off and the filtrate was evaporated on a rotary evaporator to afford a residue, which was co-evaporated with CH_2_Cl_2_ (10 mL × 3) on a rotary evaporator and further dried in vacuo at room temperature to yield the pure product **1a**–**1x** or **1ha**–**1hi**.

*Sodium 2-((5-bromo-4-ethyl-4H-1,2,4-triazol-3-yl)thio)acetate* (**1a**): White solid; 0.55 g (100%); m.p. 203 °C (dec). ^1^H-NMR (DMSO-*d*_6_, 400 MHz) δ: 3.62 (s, 2H), 3.50 (s, 3H). ^13^C-NMR (DMSO-*d*_6_, 100 MHz) δ: 169.38, 153.59, 129.80, 40.08, 31.71. ESI-HRMS [M − Na]^–^: (*m/z*) calcd. for C_5_H_5_BrN_3_O_2_S: 249.9291 (^79^Br), found: 249.9289; calcd. for C_5_H_5_BrN_3_O_2_S: 251.9271 (^81^Br), found: 251.9263. HPLC purity: 97.78%.

*Sodium 2-((5-bromo-4-n-propyl-4H-1,2,4-triazol-3-yl)thio)acetate* (**1b**): White solid; 0.58 g (100%); m.p. 106–108 °C (dec). ^1^H-NMR (DMSO-*d*_6_, 400 MHz) δ: 3.94 (q, *J =* 7.2 Hz, 2H), 3.66 (s, 2H), 1.23 (t, *J =* 7.2 Hz, 3H). ^13^C-NMR (DMSO-*d*_6_, 100 MHz) δ: 169.46, 153.07, 128.63, 40.29, 40.03, 14.47. ESI-HRMS [M − Na]^–^: (*m/z*) calcd. for C_6_H_7_BrN_3_O_2_S: 263.9448 (^79^Br), found: 263.9446; calcd. for C_6_H_7_BrN_3_O_2_S: 265.9427 (^81^Br), found: 265.9425. HPLC purity: 99.02%.

*Sodium 2-((5-bromo-4-(cyclohexylmethyl)-4H-1,2,4-triazol-3-yl)thio)acetate* (**1c**): White solid; 0.71 g (99%); m.p. 135 °C (dec). ^1^H-NMR (DMSO-*d*_6_, 400 MHz) δ: 3.73 (d, *J =* 7.2 Hz, 2H), 3.68 (s, 2H), 1.61–1.77 (m, 4H), 1.48–1.51 (m, 2H), 1.13–1.17 (m, 3H), 0.98–1.03 (m, 2H). ^13^C-NMR (DMSO-*d*_6_, 100 MHz) δ: 169.41, 153.91, 129.38, 50.62, 40.10, 37.35, 29.88, 25.61, 25.04. ESI-HRMS [M − Na]^–^: (*m/z*) calcd. for C_11_H_15_BrN_3_O_2_S: 332.0074 (^79^Br), found: 332.0241; calcd. for C_11_H_15_BrN_3_O_2_S: 334.0053 (^81^Br), found: 334.0238. HPLC purity: 98.27%.

*Sodium 2-((4-(1-adamantylmethyl)-5-bromo-4H-1,2,4-triazol-3-yl)thio)acetate* (**1d**): White solid; 0.82 g (100%); m.p. 194 °C (dec). ^1^H-NMR (DMSO-*d*_6_, 400 MHz) δ: 3.63 (s, 2H), 1.95 (s, 3H), 1.62–1.65 (m, 3H), 1.53–1.58 (m, 9H). ^13^C-NMR (DMSO-*d*_6_, 100 MHz) δ: 169.35, 155.05, 130.34, 56.18, 40.65, 40.36, 35.94, 35.46, 27.53. ESI-HRMS [M − Na]^–^: (*m/z*) calcd. for C_15_H_19_BrN_3_O_2_S: 384.0387 (^79^Br), found: 384.0393; calcd. for C_15_H_19_BrN_3_O_2_S: 386.0366 (^81^Br), found: 386.0375. HPLC purity: 98.41%.

*Sodium 2-((4-benzyl-5-bromo-4H-1,2,4-triazol-3-yl)thio)acetate* (**1e**): White solid; 0.70 g (100%); m.p. 119–121 °C. ^1^H-NMR (DMSO-*d*_6_, 400 MHz) δ: 7.35–7.39 (m, 2H), 7.32 (d, *J =* 7.2 Hz, 1H), 7.13–7.15 (m, 2H), 5.17 (s, 2H), 3.60 (s, 2H). ^13^C-NMR (DMSO-*d*_6_, 100 MHz) δ: 168.26, 154.27, 134.87, 129.42, 128.81, 127.94, 126.71, 47.87, 40.61. ESI-HRMS [M − Na]^–^: (*m/z*) calcd. for C_11_H_9_BrN_3_O_2_S: 325.9604 (^79^Br), found: 325.9591; calcd. for C_11_H_9_BrN_3_O_2_S: 327.9584 (^81^Br), found: 327.9574. HPLC purity: 99.14%.

*Sodium 2-((5-bromo-4-(3-pyridinylmethyl)-4H-1,2,4-triazol-3-yl)thio)acetate* (**1f**): White solid; 0.68 g (97%); m.p. 109–111 °C. ^1^H-NMR (DMSO-*d*_6_, 400 MHz) δ: 8.53 (m, 1H), 8.48 (d, *J =* 1.6 Hz, 1H) 7.56 (d, *J =* 8.0 Hz, 1H), 7.39–7.42 (m, 1H), 5.25 (s, 2H), 3.65 (s, 2H). ^13^C-NMR (DMSO-*d*_6_, 100 MHz), δ: 169.02, 154.06, 149.31, 148.38, 134.80, 130.69, 129.46, 123.90, 45.75, 40.33. ESI-HRMS [M − Na]^–^: (*m/z*) calcd. for C_10_H_8_BrN_4_O_2_S: 326.9557 (^79^Br), found: 326.9552; calcd. for C_10_H_8_BrN_4_O_2_S: 328.9536 (^81^Br), found: 328.9535. HPLC purity: 98.56%.

*Sodium 2-((5-bromo-4-(2-furanylmethyl)-4H-1,2,4-triazol-3-yl)thio)acetate* (**1g**): White solid; 0.68 g (100%); m.p. 108–111 °C. ^1^H-NMR (DMSO-*d*_6_, 400 MHz) δ: 7.64 (d, *J =* 1.2 Hz, 1H), 6.440–6.444 (m, 2H), 5.19 (s, 2H), 3.62 (s, 2H). ^13^C-NMR (DMSO-*d*_6_, 100 MHz) δ: 169.45, 153.85, 147.56, 143.71, 129.31, 110.80, 109.69, 41.66, 40.40. ESI-HRMS [M − Na]^–^: (*m/z*) calcd. for C_9_H_7_BrN_3_O_3_S: 315.9397 (^79^Br), found: 315.9392; calcd. for C_9_H_7_BrN_3_O_3_S: 317.9377 (^81^Br), found: 317.9370. HPLC purity: 97.68%.

*Sodium 2-((5-bromo-4-(2-thiophenylmethyl)-4H-1,2,4-triazol-3-yl)thio)acetate* (**1h**): White solid; 0.71 g (100%); m.p. 122 °C (dec). ^1^H-NMR (DMSO-*d*_6_, 400 MHz) δ: 7.51–7.52 (m, 1H), 7.11 (d, *J =* 2.8 Hz, 1H), 7.01 (dd, *J =* 3.6 Hz and 5.2 Hz, 1H), 5.36 (s, 2H), 3.65 (s, 2H). ^13^C-NMR (DMSO-*d*_6_, 100 MHz) δ: 169.00, 153.64, 136.88, 129.01, 127.71, 127.14, 127.04, 43.31, 40.67. ESI-HRMS [M − Na]^–^: (*m/z*) calcd. for C_9_H_7_BrN_3_O_2_S_2_: 331.9169 (^79^Br), found: 331.9167; calcd. for C_9_H_7_BrN_3_O_2_S_2_: 333.9148 (^81^Br), found: 333.9141. HPLC purity: 98.85%.

*Sodium 2-((4-(biphenyl-4-methyl)-5-bromo-4H-1,2,4-triazol-3-yl)thio)acetate* (**1i**): White solid; 0.85 g (100%); m.p. 199–201 °C. ^1^H-NMR (DMSO-*d*_6_, 400 MHz) δ: 7.64–7.68 (m, 4H), 7.45 (t, *J =* 7.6 Hz, 2H), 7.35 (t, *J =* 7.2 Hz, 1H), 7.23 (d, *J =* 8.4 Hz, 2H), 5.22 (s, 2H), 3.63 (s, 2H). ^13^C-NMR (DMSO-*d*_6_, 100 MHz) δ: 168.61, 154.23, 139.85, 139.44, 134.00, 129.48, 128.91, 127.58, 127.36, 127.12, 126.65, 47.63, 40.49. ESI-HRMS [M − Na]^–^: (*m/z*) calcd. for C_17_H_13_BrN_3_O_2_S: 401.9917 (^79^Br), found: 401.9913; calcd. for C_17_H_13_BrN_3_O_2_S: 403.9897 (^81^Br), found: 403.9881. HPLC purity: 99.34%.

*Sodium 2-((5-bromo-4-(diphenylmethyl)-4H-1,2,4-triazol-3-yl)thio)acetate* (**1j**): White solid; 0.85 g (100%); m.p. 195 °C (dec). ^1^H-NMR (DMSO-*d*_6_, 400 MHz) δ: 7.31–7.40 (m, 6H), 7.23–7.25 (m, 4H), 6.89 (s, 1H), 3.57 (s, 2H). ^13^C-NMR (DMSO-*d*_6_, 100 MHz) δ: 169.90, 162.42, 137.95, 129.96, 128.55, 128.18, 128.10, 65.42, 38.44. ESI-HRMS [M − Na]^–^: (*m/z*) calcd. for C_17_H_13_BrN_3_O_2_S: 401.9917 (^79^Br), found: 401.9863; calcd. for C_17_H_13_BrN_3_O_2_S: 403.9897 (^81^Br), found: 403.9845. HPLC purity: 98.86%.

*Sodium 2-((4-(benzo[b]furan-5-methyl)-5-bromo-4H-1,2,4-triazol-3-yl)thio)acetate* (**1k**): White solid; 0.78 g (100%); m.p. 92 °C (dec). ^1^H-NMR (DMSO-*d*_6_, 400 MHz) δ: 8.00 (d, *J =* 2.0 Hz, 1H), 7.60 (d, *J =* 8.8 Hz, 1H), 7.42 (d, *J =* 0.8 Hz, 1H), 7.17 (dd, *J =* 1.6 Hz and 8.8 Hz, 1H), 6.965–6.970 (m, 1H), 5.26 (s, 2H), 3.64 (s, 2H). ^13^C-NMR (DMSO-*d*_6_, 100 MHz) δ: 168.51, 154.21, 153.79, 146.83, 129.59, 129.39, 127.56, 123.34, 119.71, 111.61, 106.76, 47.99, 40.49. ESI-HRMS [M − Na]^–^: (*m/z*) calcd. for C_13_H_9_BrN_3_O_3_S: 365.9553 (^79^Br), found: 365.9570; calcd. for C_13_H_9_BrN_3_O_3_S: 367.9533 (^81^Br), found: 367.9547. HPLC purity: 99.67%.

*Sodium 2-((5-bromo-4-((4-cyano-1-naphthyl)methyl)-4H-1,2,4-triazol-3-yl)thio)acetate* (**1l**): White solid; 0.85 g (100%); m.p. 138 °C (dec). ^1^H-NMR (DMSO-*d*_6_, 400 MHz) δ: 8.39 (d, *J =* 8.0 Hz, 1H), 8.20 (d, *J =* 8.0 Hz, 1H), 8.09 (d, *J =* 7.2 Hz, 1H), 7.85–7.93 (m, 2H), 6.64 (d, *J =* 7.6 Hz, 1H), 5.85 (s, 2H), 3.65 (s, 2H). ^13^C-NMR (DMSO-*d*_6_, 100 MHz) δ: 168.82, 154.57, 137.14, 133.01, 131.43, 130.14, 129.43, 129.25, 128.50, 125.12, 124.10, 121.51, 117.30, 109.04, 45.96, 40.54. ESI-HRMS [M − Na]^–^: (*m/z*) calcd. for C_16_H_10_BrN_4_O_2_S: 400.9713 (^79^Br), found: 400.9714; calcd. for C_16_H_10_BrN_4_O_2_S: 402.9693 (^81^Br), found: 402.9677. HPLC purity: 98.53%.

*Sodium 2-((5-bromo-4-(2-naphthylmethyl)-4H-1,2,4-triazol-3-yl)thio)acetate* (**1m**): White solid; 0.78 g (98%); m.p. 118 °C (dec). ^1^H-NMR (DMSO-*d*_6_, 400 MHz) δ: 7.87–7.94 (m, 3H), 7.60 (s, 1H), 7.50–7.54 (m, 2H), 7.32–7.34 (m, 1H), 5.36 (s, 2H), 3.69 (s, 2H). ^13^C-NMR (DMSO-*d*_6_, 100 MHz) δ: 169.36, 153.98, 132.68, 132.40, 132.39, 129.79, 128.69, 127.80, 127.62, 126.65, 126.43, 125.50, 124.56, 48.19, 39.82. ESI-HRMS [M − Na]^–^: (*m/z*) calcd. for C_15_H_11_BrN_3_O_2_S: 375.9761 (^79^Br), found: 375.9777; calcd. for C_15_H_11_BrN_3_O_2_S: 377.9740 (^81^Br), found: 377.9740. HPLC purity: 97.62%.

*Sodium 2-((5-bromo-4-(quinoline-6-methyl)-4H-1,2,4-triazol-3-yl)thio)acetate* (**1n**): White solid; 0.80 g (100%); m.p. 122 °C (dec). ^1^H-NMR (DMSO-*d*_6_, 400 MHz) δ: 8.90 (s, 1H), 8.37 (d, *J =* 8.0 Hz, 1H), 8.04 (d, *J =* 8.4 Hz, 1H), 7.65 (s, 1H), 7.60 (d, *J =* 8.4 Hz, 1H), 7.53 (s, 1H), 5.40 (s, 2H), 3.67 (s, 2H). ^13^C-NMR (DMSO-*d*_6_, 100 MHz) δ: 168.72, 154.28, 150.90, 147.13, 136.04, 133.13, 129.70, 129.62, 128.10, 127.63, 125.59, 121.95, 47.86, 40.45. ESI-HRMS [M − Na]^–^: (*m/z*) calcd. for C_14_H_10_BrN_4_O_2_S: 376.9713 (^79^Br), found: 376.9702; calcd. for C_14_H_10_BrN_4_O_2_S: 378.9693 (^81^Br), found: 378.9676. HPLC purity: 99.14%.

*Sodium 2-((4-((5-bromo-4-cyano-1-naphthyl)methyl)-4H-1,2,4-triazol-3-yl)thio)-2-methylpropionate* (**1o**): White solid; 0.91 g (100%); m.p. 143 °C (dec). ^1^H-NMR (DMSO-*d*_6_, 400 MHz) δ: 8.33 (d, *J =* 8.4 Hz, 1H), 8.10 (d, *J =* 8.4 Hz, 1H), 8.03 (d, *J =* 7.2 Hz, 1H), 7.78 (t, *J =* 7.4 Hz, 1H), 7.70 (t, *J =* 7.6 Hz, 1H), 6.45 (d, *J =* 7.6 Hz, 1H), 5.91 (s, 2H), 1.48 (s, 6H). ^13^H-NMR (DMSO-*d*_6_, 100 MHz) δ: 174.54, 151.47, 137.46, 132.83, 131.80, 131.19, 129.06, 128.93, 128.21, 124.83, 123.80, 121.12, 117.23, 108.92, 56.64, 46.34, 26.83. ESI-HRMS [M − Na]^–^: (*m/z*) calcd. for C_18_H_14_BrN_4_O_2_S: 429.0026 (^79^Br), found: 429.0025; calcd. for C_18_H_14_BrN_4_O_2_S: 431.0006 (^81^Br), found: 431.0006. HPLC purity: 98.86%.

*Sodium 1-((5-bromo-4-((4-cyano-1-naphthyl)methyl)-4H-1,2,4-triazol-3-yl)thio)cyclobutanecarboxylate* (**1p**): White solid; 0.93 g (100%); m.p. 136 °C (dec). ^1^H-NMR (DMSO-*d*_6_, 400 MHz) δ: 8.34 (d, *J =* 8.4 Hz, 1H), 8.15 (d, *J =* 8.0 Hz, 1H), 8.05 (d, *J =* 7.6 Hz, 1H), 7.82–7.86 (m, 1H), 7.76–7.80 (m, 1H), 6.51 (d, *J =* 7.6 Hz, 1H), 5.94 (s, 2H), 2.43–2.49 (m, 2H), 2.22–2.29 (m, 2H), 1.82–1.85 (m, 2H). ^13^C-NMR (DMSO-*d*_6_, 100 MHz) δ: 174.74, 153.07, 137.73, 732.94, 131.34, 131.27, 129.37, 129.10, 128.47, 125.03, 124.13, 121.33, 117.28, 108.88, 60.10, 46.17, 33.07, 15.67. ESI-HRMS [M − Na]^–^: (*m/z*) calcd. for C_19_H_14_BrN4O_2_S: 441.0026 (^79^Br), found: 441.0029; calcd. for C_19_H_14_BrN4O_2_S: 443.0006 (^81^Br), found: 443.0008. HPLC purity: 99.30%.

*Sodium 2-((5-bromo-1-((4-cyano-1-naphthyl)methyl)-1H-imidazol-2-yl)thio)acetate* (**1q**): White solid; 0.85 g (100%); m.p. 161 °C (dec). ^1^H-NMR (DMSO-*d*_6_, 400 MHz) δ: 8.43 (d, *J =* 8.4 Hz, 1H), 8.19 (d, *J =* 8.0 Hz, 1H), 8.09 (d, *J =* 7.6 Hz, 1H), 7.84–7.92 (m, 2H), 7.22 (s, 2H), 6.47 (d, *J =* 7.6 Hz, 1H), 5.87 (s, 2H), 3.53 (s, 2H). ^13^C-NMR (DMSO-*d*_6_, 100 MHz) δ: 169.55, 144.81, 138.85, 133.04, 131.38, 129.64, 129.32, 129.22, 128.38, 125.07, 124.08, 121.24, 117.40, 108.64, 103.57, 46.24, 41.22. ESI-HRMS [M − Na]^–^: (*m/z*) calcd. for C_17_H_11_BrN_3_O_2_S: 399.9761 (^79^Br), found: 399.9757; calcd. for C_17_H_11_BrN_3_O_2_S: 401.9740 (^81^Br), found: 401.9740. HPLC purity: 97.98%.

*Sodium 2-((5-bromo-1-((4-cyano-1-naphthyl)methyl)-1H-imidazol-2-yl)thio)-2-methylpropionate* (**1r**): White solid; 0.90 g (100%); m.p. 243 °C (dec). ^1^H-NMR (DMSO-*d*_6_, 400 MHz) δ: 8.45 (d, *J =* 8.0 Hz, 1H), 8.18 (d, *J =* 7.6 Hz, 1H), 8.07 (d, *J =* 7.6 Hz, 1H), 7.83–7.91 (m, 2H), 7.30 (s, 1H), 6.31 (d, *J =* 7.6 Hz, 1H), 5.96 (s, 2H), 1.36 (s, 6H). ^13^C-NMR (DMSO-*d*_6_, 100 MHz) δ: 175.57, 143.52, 139.40, 132.95, 131.31, 130.19, 129.26, 129.12, 128.37, 125.00, 124.17, 121.16, 117.41, 108.50, 104.79, 59.03, 46.62, 28.10. ESI-HRMS [M − Na]^–^: (*m/z*) calcd. for C_19_H_15_BrN_3_O_2_S: 428.0074 (^79^Br), found: 428.0071; calcd. for C_19_H_15_BrN_3_O_2_S: 430.0053 (^81^Br), found: 430.0053. HPLC purity: 98.26%.

*Sodium 1-((5-bromo-1-((4-cyano-1-naphthyl)methyl)-1H-imidazol-2-yl)thio)cyclobutanecarboxylate* (**1s**): White solid; 0.93 g (100%); m.p. 285 °C (dec). ^1^H-NMR (DMSO-*d*_6_, 400 MHz) δ: 8.41 (d, *J =* 8.4 Hz, 1H), 8.18 (d, *J =* 8.0 Hz, 1H), 8.07 (d, *J =* 7.6 Hz, 1H), 7.82–7.91 (m, 2H), 7.33 (s, 1H), 6.35 (d, *J =* 7.6 Hz, 1H), 5.98 (s, 2H), 2.36–2.42 (m, 2H), 2.16–2.23 (m, 2H), 1.75–1.81 (m, 1H), 1.62–1.68 (m, 1H). ^13^C-NMR (DMSO-*d*_6_, 100 MHz) δ: 175.04, 143.20, 139.38, 132.98, 131.31, 130.38, 129.27, 129.11, 128.36, 125.00, 124.14, 121.17, 117.40, 108.48, 105.12, 59.84, 46.62, 32.83, 15.63. ESI-HRMS [M − Na]^–^: (*m/z*) calcd. for C_20_H_15_BrN_3_O_2_S: 440.0074 (^79^Br), found: 440.0070; calcd. for C_20_H_15_BrN_3_O_2_S: 442.0053 (^81^Br), found: 442.0052. HPLC purity: 99.15%.

*Sodium 2-((3-((4-cyano-1-naphthyl)methyl)pyridin-4-yl)thio)acetate* (**1t**): White solid; 0.71 g (100%); m.p. 268 °C (dec). ^1^H-NMR (DMSO-*d*_6_, 400 MHz) δ: 8.24–8.26 (m, 2H), 8.16 (d, *J =* 8.0 Hz, 1H), 8.06 (d, *J =* 3.6 Hz, 1H), 7.95 (s, 1H), 7.82 (t, *J =* 7.6 Hz, 1H), 7.75 (t, *J =* 7.6 Hz, 1H), 7.32 (d, *J =* 5.2 Hz, 1H), 7.19 (d, *J =* 7.2 Hz, 1H), 4.50 (s, 2H), 3.48 (s, 2H). ^13^C-NMR (DMSO-*d*_6_, 100 MHz) δ: 169.29, 150.24, 148.80, 147.62, 141.78, 133.01, 131.72, 131.18, 130.33, 128.87, 128.06, 125.52, 125.04, 124.91, 119.63, 117.74, 107.80, 37.60, 33.16. ESI-HRMS [M − Na]^–^: (*m/z*) calcd. for C_19_H_13_N_2_O_2_S: 333.0703, found: 333.0728. HPLC purity: 98.84%.

*Sodium 2-((3-((4-cyano-1-naphthyl)methyl)pyridin-4-yl)thio)-2-methylpropionate* (**1u**): White solid; 0.77 g (100%); m.p. 273 °C (dec). ^1^H-NMR (DMSO-*d*_6_, 400 MHz) δ: 8.23–8.25 (m, 2H), 8.16 (d, *J =* 8.0 Hz, 1H), 8.07 (d, *J =* 7.6 Hz, 1H), 7.98 (s, 1H), 7.82 (t, *J =* 7.6 Hz, 1H), 7.72–7.76 (m, 2H), 7.16 (d, *J =* 7.6 Hz, 1H), 4.52 (s, 2H), 1.43 (s, 6H). ^13^C-NMR (DMSO-*d*_6_, 100 MHz) δ: 174.84, 149.42, 148.58, 146.99, 142.44, 133.00, 132.35, 131.70, 131.14, 128.86, 127.99, 125.62, 125.01, 123.85, 117.74, 107.70, 54.17, 33.40, 27.69. ESI-HRMS [M − Na]^–^: (*m/z*) calcd. for C_21_H_17_N_2_O_2_S: 361.1016, found: 361.1025. HPLC purity: 99.07%.

*Sodium 1-((3-((4-cyano-1-naphthyl)methyl)pyridin-4-yl)thio)cyclobutanecarboxylate* (**1v**): White solid; 0.79 g (100%); m.p. 296 °C (dec). ^1^H-NMR (DMSO-*d*_6_, 400 MHz) δ: 8.24 (d, *J =* 8.0 Hz, 1H), 8.15–8.18 (m, 2H), 8.08 (d, *J =* 7.2 Hz, 1H), 7.91 (s, 1H), 7.82 (t, *J =* 7.4 Hz, 1H), 7.75 (t, *J =* 7.6 Hz, 1H), 7.60 (d, *J =* 5.6 Hz, 1H), 7.23 (d, *J =* 7.2 Hz, 1H), 4.46 (s, 2H), 2.69–2.77 (m, 2H), 1.93–2.02 (m, 3H), 1.79–1.86 (m, 1H). ^13^C-NMR (DMSO-*d*_6_, 100 MHz) δ: 174.51, 149.45, 148.94, 146.98, 141.93, 132.99, 131.70, 131.18, 130.38, 128.84, 127.99, 125.66, 125.01, 124.97, 121.42, 117.71, 107.77, 54.07, 33.21, 32.68, 16.08. ESI-HRMS [M − Na]^–^: (*m/z*) calcd. for C_22_H_17_N_2_O_2_S: 373.1016, found: 373.1003. HPLC purity: 98.34%.

*Sodium 2-((5-bromo-1-((2-thiophenyl)methyl)-1H-imidazol-2-yl)thio)-2-methylpropionate* (**1w**): White solid; 0.77 g (100%); m.p. 75 °C (dec). ^1^H-NMR (DMSO-*d*_6_, 400 MHz) δ: 7.43 (d, *J =* 8.8 Hz, 1H), 7.11 (s, 1H), 6.95–6.98 (m, 2H), 5.49 (s, 2H), 1.33 (s, 6H). ^13^C-NMR (MeOH-*d*_4_, 100 MHz) δ: 179.91, 142.27, 140.00, 131.11, 127.92, 127.68, 126.85, 106.91, 58.53, 45.72, 27.90. ESI-HRMS [M − Na]^–^: (*m/z*) calcd. for C_12_H_12_BrN_2_O_2_S_2_: 358.9528 (^79^Br), found: 358.9526; calcd. for C_12_H_12_BrN_2_O_2_S_2_: 360.9509 (^81^Br), found: 360.9504. HPLC purity: 97.64%.

*Sodium 1-((5-bromo-1-((2-thiophenyl)methyl)-1H-imidazol-2-yl)thio)cyclobutanecarboxylate* (**1x**): White solid; 0.79 g (100%); m.p. 210 °C (dec). ^1^H-NMR (DMSO-*d*_6_, 400 MHz) δ: 7.43–7.44 (m, 1H), 7.14 (s, 1H), 7.01 (d, *J =* 7.2 Hz, 1H), 6.95–6.97 (m, 1H), 5.49 (s, 2H), 2.34–2.41 (m, 2H), 2.10–2.17 (m, 2H), 1.73–1.79 (m, 1H), 1.61–1.67 (m, 1H). ^13^C-NMR (MeOH-*d*_4_, 100 MHz) δ: 180.25, 142.07, 140.02, 131.19, 128.03 127.70, 126.91, 107.11, 59.48, 45.72, 33.50, 15.97. ESI-HRMS [M − Na]^–^: (*m/z*) calcd. for C_13_H_12_BrN_2_O_2_S_2_: 370.9529 (^79^Br), found: 370.9527; calcd. for C_13_H_12_BrN_2_O_2_S_2_: 372.9509 (^81^Br), found: 372.9504. HPLC purity: 97.98%.

*Sodium 2-((5-bromo-4-((5-chloro-2-thiophenyl)methyl)-4H-1,2,4-triazol-3-yl)thio)acetate* (**1ha**): White solid; 0.78 g (100%); m.p. 97 °C (dec). ^1^H-NMR (DMSO-*d*_6_, 400 MHz) δ: 7.03 (d, *J =* 3.6 Hz, 1H), 6.99 (d, *J =* 3.6 Hz, 1H), 5.32 (s, 2H), 3.75 (s, 2H). ESI-HRMS [M − Na]^–^: (*m/z*) calcd. for C_9_H_6_BrClN_3_O_2_S_2_: 365.8779 (^79^Br), found: 365.8788; calcd. for C_9_H_6_BrClN_3_O_2_S_2_: 367.8758 (^81^Br), found: 367.8765. HPLC purity: 96.26%.

*Sodium 2-((5-bromo-4-((5-methyl-2-thiophenyl)methyl)-4H-1,2,4-triazol-3-yl)thio)acetate* (**1hb**): White solid; 0.74 g (100%); m.p. 157 °C (dec). ^1^H-NMR (DMSO-*d*_6_, 400 MHz) δ: 6.90 (d, *J =* 3.2 Hz, 1H), 6.67–6.68 (m, 1H), 5.25 (s, 2H), 3.65 (s, 2H), 2.38 (s, 3H). ^13^C-NMR (DMSO-*d*_6_, 100 MHz) δ: 168.83, 153.46, 140.56, 134.32, 128.93, 127.74, 125.20, 43.51, 40.38, 14.86. ESI-HRMS [M − Na]^–^: (*m/z*) calcd. for C_10_H_9_BrN_3_O_2_S_2_: 345.9325 (^79^Br), found: 345.9321; calcd. for C_10_H_9_BrN_3_O_2_S_2_: 347.9305 (^81^Br), found: 347.9295. HPLC purity: 98.80%.

*Sodium 2-((5-bromo-4-((5-ethyl-2-thiophenyl)methyl)-4H-1,2,4-triazol-3-yl)thio)acetate* (**1hc**): White solid; 0.77 g (100%); m.p. 178–180 °C. ^1^H-NMR (DMSO-*d*_6_, 400 MHz) δ: 6.92 (d, *J =* 3.2 Hz, 1H), 6.71 (d, *J =* 3.2 Hz, 1H), 5.26 (s, 2H), 3.66 (s, 2H), 2.74 (q, *J =* 7.5 Hz, 2H), 1.18 (t, *J =* 7.4 Hz, 3H). ^13^C-NMR (DMSO-*d*_6_, 100 MHz) δ: 169.15, 153.59, 148.05, 133.99, 128.95, 127.64, 123.45, 43.57, 40.58, 22.73, 15.66. ESI-HRMS [M − Na]^–^: (*m/z*) calcd. for C_11_H_11_BrN_3_O_2_S_2_: 359.9482 (^79^Br), found: 359.9479; calcd. for C_11_H_11_BrN_3_O_2_S_2_: 361.9461 (^81^Br), found: 361.9462. HPLC purity: 98.06%.

*Sodium 2-((5-bromo-4-((3-methyl-2-thiophenyl)methyl)-4H-1,2,4-triazol-3-yl)thio)acetate* (**1hd**): White solid; 0.74 g (100%); m.p. 158 °C (dec). ^1^H-NMR (DMSO-*d*_6_, 400 MHz) δ: 7.38 (d, *J =* 5.2 Hz, 1H), 6.86 (d, *J =* 5.2 Hz, 1H), 5.29 (s, 2H), 3.62 (s, 2H), 2.30 (s, 3H). ^13^C-NMR (DMSO-*d*_6_, 100 MHz) δ: 168.91, 153.65, 135.54, 130.86, 130.17, 129.12, 125.05, 42.37, 40.68, 13.80. ESI-HRMS [M − Na]^–^: (*m/z*) calcd. for C_10_H_9_BrN_3_O_2_S_2_ : 345.9325 (^79^Br), found: 345.9318; calcd. for C_10_H_9_BrN_3_O_2_S_2_ : 347.9305 (^81^Br), found: 347.9294. HPLC purity: 98.33%.

*Sodium 2-((5-bromo-4-((5-bromo-4-methyl-2-thiophenyl)methyl)-4H-1,2,4-triazol-3-yl)thio)acetate* (**1he**): White solid; 0.90 g (100%); m.p. 87 °C (dec). ^1^H-NMR (DMSO-*d*_6_, 400 MHz) δ: 6.90 (s, 1H), 5.27 (s, 2H), 3.64 (s, 2H), 2.09 (s, 3H). ^13^C-NMR (DMSO-*d*_6_, 100 MHz) δ: 168.82, 153.62, 137.17, 136.60, 129.75, 128.98, 108.83, 43.35, 40.70, 14.77. ESI-HRMS [M − Na]^–^: (*m/z*) calcd. for C_10_H_8_Br_2_N_3_O_2_S_2_: 425.8410, found: 425.8399. HPLC purity: 97.71%.

*Sodium 2-((5-bromo-4-((3-thiophenyl)methyl)-4H-1,2,4-triazol-3-yl)thio)acetate* (**1hf**): White solid; 0.71 g (100%); m.p. 124–126 °C. ^1^H-NMR (DMSO-*d*_6_, 400 MHz) δ: 7.55–7.57 (m, 1H), 7.34 (d, *J =* 1.6 Hz, 1H), 5.15 (s, 2H), 3.66 (s, 2H). ^13^C-NMR (DMSO-*d*_6_, 100 MHz) δ: 169.34, 153.69, 135.44, 129.23, 127.58, 126.57, 123.78, 43.91, 40.15. ESI-HRMS [M − Na]^–^: (*m/z*) calcd. for C_9_H_7_BrN_3_O_2_S_2_: 331.9169 (^79^Br), found: 331.9166; calcd. for C_9_H_7_BrN_3_O_2_S_2_: 333.9148 (^81^Br), found: 333.9140. HPLC purity: 99.40%.

*Sodium 2-((5-bromo-4-((5-phenyl-2-thiophenyl)methyl)-4H-1,2,4-triazol-3-yl)thio)acetate* (**1hg**): White solid; 0.86 g (100%); m.p. 212 °C(dec). ^1^H-NMR (DMSO-*d*_6_, 400 MHz) δ: 7.59–7.60 (m, 2H), 7.37–7.41 (m, 3H), 7.30 (t, *J =* 7.2 Hz, 1H), 7.12 (d, *J =* 3.6 Hz, 1H), 5.37 (s, 2H), 3.67 (s, 2H). ^13^C-NMR (DMSO-*d*_6_, 100 MHz) δ: 168.50, 153.77, 144.13, 136.31, 133.14, 129.12, 129.00, 127.92, 125.32, 123.45, 43.53, 40.85. ESI-HRMS [M − Na]^–^: (*m/z*) calcd. for C_15_H_11_BrN_3_O_2_S_2_: 407.9482 (^79^Br), found: 407.9498; calcd. for C_15_H_11_BrN_3_O_2_S_2_: 409.9461 (^81^Br), found: 409.9443. HPLC purity: 99.12%.

*Sodium 2-((5-bromo-4-((3-bromobenzo[b]thiophen-2-yl)methyl)-4H-1,2,4-triazol-3-yl)thio)acetate* (**1hh**): White solid; 0.97 g (100%); m.p. 99 °C(dec). ^1^H-NMR (DMSO-*d*_6_, 400 MHz) δ: 8.02 (d, *J =* 8.0 Hz, 1H), 7.77 (d, *J =* 8.0 Hz, 1H), 7.52–7.56 (m, 1H), 7.45–7.49 (m, 1H), 5.54 (s, 2H), 3.71 (s, 2H). ^13^C-NMR (DMSO-*d*_6_, 100 MHz) δ: 169.23, 153.82, 137.07, 136.75, 133.59, 129.58, 126.27, 125.90, 123.27, 122.72, 107.03, 44.21, 40.23. ESI-HRMS [M − Na]^–^: (*m/z*) calcd. for C_13_H_8_Br_2_N_3_O_2_S_2_: 461.8410, found: 461.8420. HPLC purity: 97.84%.

*Sodium 2-((5-bromo-4-((dibenzo[b,d]thiophen-4-yl)methyl)-4H-1,2,4-triazol-3-yl)thio)acetate* (**1hi**): White solid; 0.91 g (100%); m.p. 74 °C(dec). ^1^H-NMR (DMSO-*d*_6_, 400 MHz) δ: 8.39–8.41 (m, 1H), 8.36 (d, *J =* 7.6 Hz, 1H), 8.05–8.08 (m, 1H), 7.51–7.57 (m, 3H), 7.01 (d, *J =* 7.6 Hz, 1H), 5.45 (s, 2H), 3.63 (s, 2H). ^13^C-NMR (DMSO-*d*_6_, 100 MHz) δ: 169.01, 154.56, 138.10, 135.94, 134.79, 130.13, 128.99, 127.48, 125.33, 125.07, 124.81, 123.02, 122.31, 121.77, 47.32, 40.37. ESI-HRMS [M − Na]^–^: (*m/z*) calcd. for C_17_H_11_BrN_3_O_2_S_2_: 431.9482 (^79^Br), found: 431.9640; calcd. for C_17_H_11_BrN_3_O_2_S_2_: 433.9461 (^81^Br), found: 433.9673. HPLC purity: 99.35%.

### 3.3. In Vitro URAT1 Inhibitory Assay

The in vitro URAT1 inhibitory activity of the compounds **1a**–**1x** and **1ha**–**1hi** as well as lesinurad (in the form of sodium salt as well) was determined by the inhibition of URAT1-mediated [8-^14^C]uric acid uptake by human embryonic kidney 293 (HEK293) cells stably expressing human URAT1 (HEK293-URAT1 cells). Thus, HEK293-URAT1 cells and mock cells (HEK293A-pcDNA3.1) were subcultured for 2–3 generations in Dulbecco’s Modified Eagle Medium (DMEM; with 10% FBS, 100 IU/mL penicillin, and 50 μg/mL streptomycin at 37 °C, 5% CO_2_, and 95% air). After incubation for 48 h, when the HEK293-URAT1 cells and mock cells were grown to confluence, the cells were treated with 0.25% trypsin for 1–2 min. Thereafter, the digested cells were diluted to a density of 1.5 × 10^5^/mL and seeded onto the 24-well cell culture plates at 1 mL/well for about 48 h. The HEK293-URAT1 cells and mock cells were pre-incubated for 10 min in HBSS (Cl^−^ free) and then incubated in the uptake solution (PBS) containing 5 μM [8-^14^C]uric acid (2 μCi/mL) with or without the tested compounds (at a series of concentrations ranging from 0.001 M to 100 M depending on the specific potency). The HEK293-URAT1 cells were then washed rapidly three times with ice-cold HBSS (Cl^−^ free) and lysed with 0.1 M aqueous NaOH solution. After that, 500-μL aliquots of cell lysates were transferred to scintillation vials and mixed with scintillation solution. The radioactivity in the cells was measured by a liquid scintillation counter (PerkinElmer, Waltham, MA, USA). 

The inhibition rates of the test compounds were calculated according to the following equation,

Inhibition rate = ((U − U_0_)/(U_c_ − U_0_)] × 100% where, U is the radioactivity corresponding to HEK293-URAT1 cell with test compound, U_c_ is the radioactivity corresponding to HEK293-URAT1 cell without test compound and U_0_ is the radioactivity corresponding to mock cell with [8-^14^C]uric acid. The concentrations of the compounds to inhibit 50% [8-^14^C]uric acid uptake by HEK293-URAT1 cells (IC_50_) were calculated with GraphPad Prism software (San Diego, CA, USA). The assays were performed in triplicate, and the IC_50_ values were expressed as mean ± SD.

## 4. Conclusions

In conclusion, a systematic SAR exploration of the diarylmethane backbone in the present study led to the identification of a highly potent URAT1 inhibitor **1h**, which was 200- and 8-fold more potent than parent lesinurad and benzbromarone, respectively (IC_50_ = 0.035 μM against human URAT1 for **1h** vs. 7.18 μM and 0.28 μM for lesinurad and benzbromarone, respectively). Compared to the potent URAT1 inhibitors discovered in earlier work, compound **1h**, possessing a novel thiophenyltriazolylmethane backbone, is the most potent URAT1 inhibitor discovered in our laboratories so far and also comparable to the most potent ones currently under development in clinical trials. The present study strongly suggests that the diarylmethane backbone represents a very promising molecular scaffold for the design of potent URAT1 inhibitors.

## Figures and Tables

**Table 1:** Results of in vitro inhibitory assay of **1a**–**1x** and **1ha**–**1hi** against human URAT1 (IC_50_).

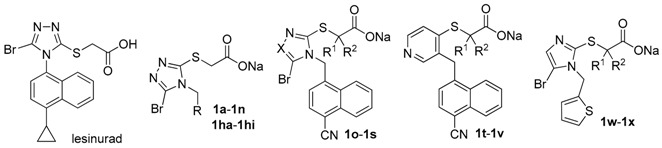					
Compd.	R	X	R^1^	R^2^	IC_50_ (μM) ^a^
lesinurad	-	-	-	-	7.18 ± 1.13 ^b^
benzbromarone	-	-	-	-	0.28 ± 0.07 ^c^
**1a**	Me	-	-	-	18.42 ± 3.36
**1b**	Et	-	-	-	4.83 ± 1.02
**1c**	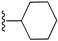	-	-	-	4.60 ± 0.88
**1d**	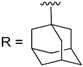	-	-	-	5.11 ± 0.79
**1e**	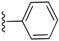	-	-	-	2.98 ± 0.60
**1f**	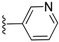	-	-	-	14.19 ± 4.71
**1g**	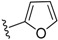	-	-	-	4.54 ± 0.69
**1h**	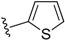	-	-	-	0.035 ± 0.01
**1i**	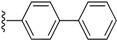	-	-	-	1.16 ± 0.34
**1j** ^d^	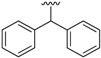	-	-	-	1.15 ± 0.25
**1k**	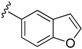	-	-	-	2.61 ± 0.40
**1l**	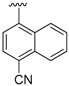	-	-	-	3.80 ± 0.78
**1m**	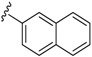	-	-	-	4.66 ± 1.04
**1n**	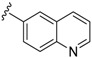	-	-	-	1.36 ± 0.24
**1ha**	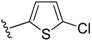	-	-	-	11.23 ± 2.36
**1hb**	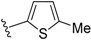	-	-	-	> 50
**1hc**	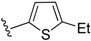	-	-	-	7.98 ± 2.14
**1hd**	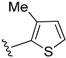	-	-	-	3.04 ± 0.84
**1he**	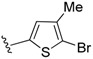	-	-	-	6.10 ± 1.17
**1hf**	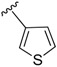	-	-	-	> 50
**1hg**	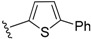	-	-	-	31.68 ± 9.34
**1hh**	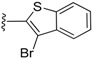	-	-	-	0.12 ± 0.03
**1hi**	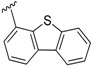	-	-	-	18.47 ± 5.41
**1o**	-	N	Me	Me	1.96 ± 0.34
**1p**	-	N	-(CH_2_)_3_-		0.59 ± 0.17
**1q**	-	C	H	H	5.55 ± 0.98
**1r**	-	C	Me	Me	0.21 ± 0.07
**1s**	-	C	-(CH_2_)_3_-		0.48 ± 0.11
**1t**	-	-	H	H	8.83 ± 1.02
**1u**	-	-	Me	Me	1.39 ± 0.42
**1v**	-	-	-(CH_2_)_3_-		1.15 ± 0.20
**1w**	-	-	Me	Me	17.36 ± 3.98
**1x**	-	-	-(CH_2_)_3_-		0.32 ± 0.07

^a^ All the experiments were performed in triplicate, and the IC_50_ values were expressed as mean ± SD. ^b^ Reported value for lesinurad: IC_50_ = 7.3 μM against human URAT1 [23]. ^c^ Reported value for benzbromarone: IC_50_ = 0.29 μM against human URAT1 [47]. ^d^ For **1j**, the substituent R should be RCH_2_ as indicated in Scheme 1.

## Supplementary Materials

Supplementary materials are available on line. The representative dose-response curves, i.e., concentration-inhibition rate curves, of lesinurad and benzbromarone for the calculation of their IC_50_ values against human URAT1 (Figure S1).
