# Supplementary material for: Systematic Structure-Activity Relationship (SAR) Exploration of Diarylmethane Backbone and Discovery of A Highly Potent Novel Uric Acid Transporter 1 (URAT1) Inhibitor
Source: Molecules. 2018 Jan 27;23(2):252. doi: 10.3390/molecules23020252 (PMC6017028; doi:10.3390/molecules23020252)
Supplement: Supplementary file 1 [file molecules-23-00252-s001.pdf]

Systematic Structure-Activity Relationship (SAR) Exploration of Diarylmethane Backbone and Discovery of a Highly Potent Novel Uric Acid Transporter 1 (URAT1) Inhibitor

Wenqing Cai [1,2,†], Jingwei Wu [2,†], Wei Liu [2], Yafei Xie [2], Yuqiang Liu [2], Shuo Zhang [3], Weiren Xu [2], Lida Tang [2], Jianwu Wang [1,*] and Guilong Zhao [2,*]

1 School of Chemistry and Chemical Engineering, Shandong University, Jinan 250100, China; caiwenqingsunny@163.com
2 Tianjin Key Laboratory of Molecular Design and Drug Discovery, Tianjin Institute of Pharmaceutical Research, Tianjin 300193, China; wujw@tjipr.com (J.W.); liuw@tjipr.com (W.L.); xieyf@tjipr.com (Y.X.); liuyq@tjipr.com (Y.L.); xuwr@tjipr.com (W.X.); tangld@tjipr.com (L.T.)
3 Shandong Key Laboratory for Special Silicon-Containing Materials, Advanced Materials Institute, Shandong Academy of Sciences, Jinan 250014, China; e50687e@163.com
† These authors contributed equally
* Correspondence: jwwang@sdu.edu.cn (J.W.); zhao_guilong@126.com (G.Z.); Tel.: +86-531-8836-2708 (J.W.); +86-22-2300-6869 (G.Z.)

Supplementary Information

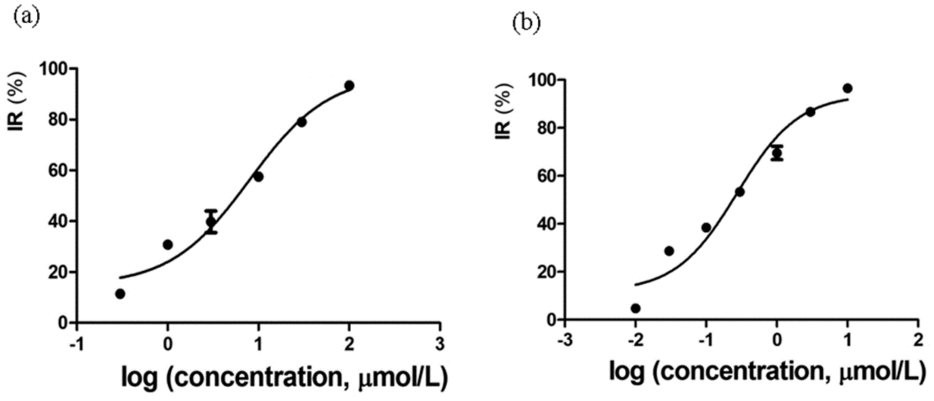

Figure S1. The representative dose-response curves of lesinurad and benzbromarone for the calculation of their IC_{50} values against human URAT1. (a) A representative dose-response curves of lesinurad. (b) A representative dose-response curves of benzbromarone. The detailed experimental procedure is shown in "3.3 In Vitro URAT1 Inhibitory Assay".
